# Qualitative Monitoring of Proto-Peptide Condensation by Differential FTIR Spectroscopy

**DOI:** 10.1021/acsearthspacechem.3c00257

**Published:** 2024-05-06

**Authors:** Keon Rezaeerod, Hanna Heinzmann, Alexis V. Torrence, Jui Patel, Jay G. Forsythe

**Affiliations:** † Department of Chemistry and Biochemistry, College of Charleston, Charleston, South Carolina 29424, United States; ‡ Analytical and Bioanalytical Chemistry, Aalen University, 73430 Aalen, Germany

**Keywords:** FTIR, wet−dry cycling, depsipeptides, prebiotic chemistry, astrobiology

## Abstract

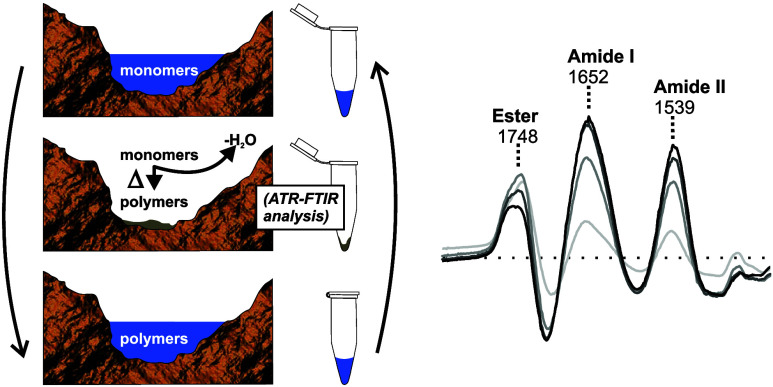

Condensation processes such as wet–dry cycling are thought to have played significant roles in the emergence of proto-peptides. Here, we describe a simple and low-cost method, differential Fourier transform infrared (FTIR) spectroscopy, for qualitative analysis of peptide condensation products in model primordial reactions. We optimize differential FTIR for depsipeptides and apply this method to investigate their polymerization in the presence of extraterrestrial dust simulants.

## Introduction

The polymers of life as we know it form by condensation, or the removal of water between monomer units.^1−7^ Abiotic condensation is generally unfavorable in aqueous solution, as water is both solvent and reaction product. However, when water activity is reduced, the reaction shifts forward toward polymers.^8,9^

Wet–dry cycling, a process that models aqueous pools drying out and rehydrating each day, is a promising approach for abiotic polymer condensation.^4,10−15^ Monomers condense as water is heated and are evaporated from the system, and then water is reintroduced to mix soluble components and break down unstable polymers. Wet–dry cycling has been used to generate multiple classes of model primordial polymers such as polyesters,^11,16^ peptides and depsipeptides,^12,17−21^ nucleic acids and related components,^22,23^ and oligosaccharides.^24^ Other strategies than wet–dry cycling can be used to reduce water activity, including but not limited to eutectic solvents,^25−28^ plausible condensing agents,^29−31^ deliquescence,^14,32^ and interfacial/aerosol chemistry.^33−35^

Various analytical techniques are suitable to confirm the presence of proto-biopolymers and investigate their composition. Nuclear magnetic resonance (NMR) provides both qualitative (e.g., major and minor products) and quantitative information (e.g., reaction yield),^36−38^ but is rather expensive to acquire and maintain. Also, data quality is dependent on sample purity and solubility. Mass spectrometry (MS) elucidates molecular weight distributions, lengths, and compositions.^39−43^ Tandem MS, or MS/MS, can reveal more detailed structural information such as monomer sequences.^44,45^ MS instrumentation and upkeep costs are also high, and isotopically labeled standards are needed for quantitative analysis. Depending on polymer type and size, separation methods such as liquid chromatography (LC),^46^ capillary electrophoresis,^47^ gel electrophoresis,^48^ size exclusion chromatography (SEC),^49,50^ gel permeation chromatography (GPC),^51^ and ion mobility spectrometry (IMS)^52^ can be used for characterization. Coupling a separation technique with MS leads to more comprehensive analysis (LC-MS, IM-MS, etc.),^45,53,54^ yet as dimensionality increases, so do costs and data complexity.

Fourier transform infrared (FTIR) spectroscopy often plays a complementary role to the above techniques as it is a bulk analysis with modest sensitivity. Yet, despite its limitations, FTIR spectroscopy is ubiquitous in laboratories as it is relatively cheap and easy to use. FTIR spectroscopy is highly suitable for the analysis of organic polymers as it establishes the major chemical functional groups.^55^ Another strength of FTIR spectroscopy is the use of attenuated total reflection (ATR) targets, which require little to no sample preparation for solids and liquids.^56^ As origins-of-life and astrobiology research fields continue to grow and diversify, FTIR spectroscopy will remain a key tool for condensation polymers, regardless of institutional type and/or educational level.

Here, we explore the use of differential FTIR spectroscopy for proto-peptide formation during wet–dry cycling. Specifically, monomer control spectra were subtracted from proto-peptide spectra to monitor chemical changes over time. This method was optimized for depsipeptides^12^ and used to evaluate their polymerization in the presence of model extraterrestrial dusts. Differential FTIR is simple, straightforward, and suitable for monitoring polymerization in complex sample matrices relevant to prebiotic chemistry and astrobiology.

## Methods

### Materials

Glycine, l-alanine, l-valine (all ≥98% purity), glycolic acid (70 wt %), and lactic acid (d/l, 85 wt %) were obtained from Sigma-Aldrich. Water was deionized to 18.2 MΩ·cm using a Thermo Barnstead MicroPure system. LC-MS-grade acetonitrile used for mass spectrometry (MS) was obtained from VWR. 2,5-Dihydroxybenzoic acid (99%; TCI America) was used as the MS matrix. LHS-1D Lunar Highlands and JEZ-1 Jezero Crater Delta Martian dust simulants were obtained from Exolith Lab. Briefly, LHS-1D model moon dust is primarily SiO_2_, Al_2_O_3_, and CaO; JEZ-1 is modeled after Jezero Crater on Mars and contains SiO_2_, MgO, FeO, Al_2_O_3_, CaO, etc. Detailed lists and abundances can be found on the vendor Web site.

No unexpected safety hazards were encountered in this study. Inhalation of simulated space dust should be avoided; a respirator is encouraged if ventilation is limited.

### Wet–Dry Cycling

Depsipeptides were formed by mixing hydroxy acid and amino acid solutions of 0.10–0.40 M and subjecting them to cycles of evaporation (cap open) and rehydration to the initial volume of 0.200 mL (cap closed). Cycling was done in 1.5 mL microcentrifuge tubes, but other containers such as ceramic spot plates or scintillation vials are also suitable. Specific wet–dry cycling procedures used in this work were as follows: for lactic acid/glycine samples, 23.5 h of heating at 85 °C (open), then 0.5 h of rehydration/incubation at RT (closed), repeat for *n* cycles; for glycolic acid/valine and lactic acid/alanine samples, 18 h of heating at 85 °C (open), then 6 h rehydration/incubation at 85 °C (closed), repeat for *n* cycles. Lactic acid/glycine cycling experiments were performed twice independently (0–12 cycles), glycolic acid/valine cycling experiments were performed three times independently (0–8 cycles), and lactic acid/alanine cycling experiments were performed twice independently with 1 mg dust (0–4 cycles). No buffers were added to adjust sample pH.

### ATR-FTIR Spectroscopy

All FTIR spectra were acquired on a PerkinElmer Frontier with diamond/ZnSe crystal Universal ATR attachment (single bounce). Products were scraped out of microcentrifuge tubes after the hot/dry evaporation step using a 0.50 mm Micro-Spade tool (tip #4; Electron Microscopy Sciences) and placed directly onto the ATR target. All spectra were background-subtracted to remove CO_2_ from ambient air and acquired in absorbance mode. FTIR settings were as follows: 4000–700 cm^–1^, 8 scans, 2 cm^–1^ resolution, 0.5 cm^–1^ data interval, knob force ∼60 (arbitrary units). Spectra were converted to .csv format in Spectrum IR software and processed using Microsoft Excel and OriginPro. Control samples, normalization, and spectral subtraction strategies are discussed in the Results and Discussion section.

### MALDI Mass Spectrometry for Validation

MS data were obtained on a refurbished Voyager DE-STR (JBI Scientific) matrix-assisted laser desorption/ionization–time-of-flight (MALDI-TOF) instrument equipped with a 337 nm nitrogen gas laser fired at 20 Hz.^57^ All spectra were acquired in positive-ion, reflector TOF mode. Other instrument settings were as follows: +20 kV acceleration voltage; 75% grid voltage; 150 ns extraction delay; *m*/*z* 200–2000. Mass spectra were converted to .txt format in Data Explorer 4.1 (Applied Biosystems) and processed using OriginPro and MSPolyCalc.^58^ MSPolyCalc settings were as follows: [M + H]^+^, [M + Na]^+^, and [M + K]^+^ ions; H α end group; OH omega end group; ±300 ppm mass tolerance; 30–80% similarity threshold, depending on the S/N of each spectrum. Median ester and amide content in depsipeptides was obtained from MSPolyCalc data and reports, which are provided in the Supporting Information as an appendix.

## Results and Discussion

### Differential FTIR Spectroscopy of Wet–Dry Cycled Samples

**Figure 1:**
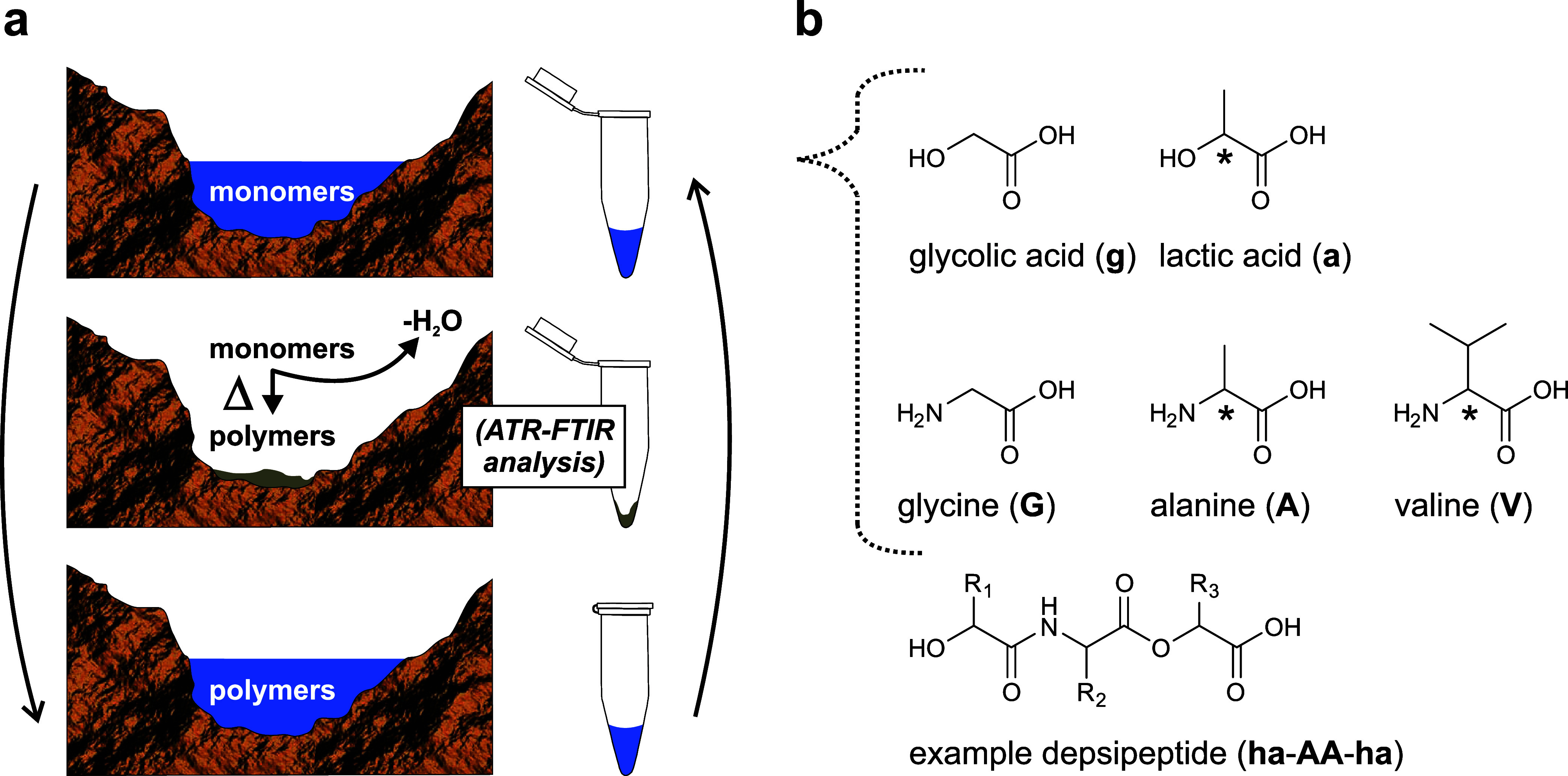
(a) Diagram of a wet–dry cycle. Monomer solutions are heated (open system), removing water and forming polymers. Water is then reintroduced and incubated with sample (closed system; temperature and length of time adjustable) to hydrolyze unstable polymers in solution. Cycles are then repeated as desired. FTIR analysis is performed after the hot/dry step, as material is in a solid and/or gel state and easy to transfer to an ATR target. (b) Monomers and polymers studied in this work; chiral centers are marked with asterisks. Hydroxy acids are abbreviated with lower case letters and amino acids are abbreviated with upper case letters. Depsipeptides are copolymers of hydroxy acids and amino acids.

In this study, two hydroxy acids and three amino acids were used to make depsipeptides by wet–dry cycling (Figure 1). FTIR spectra were acquired after drying stages as samples were amorphous solids and easier to transfer to the ATR target.

**Figure 2:**
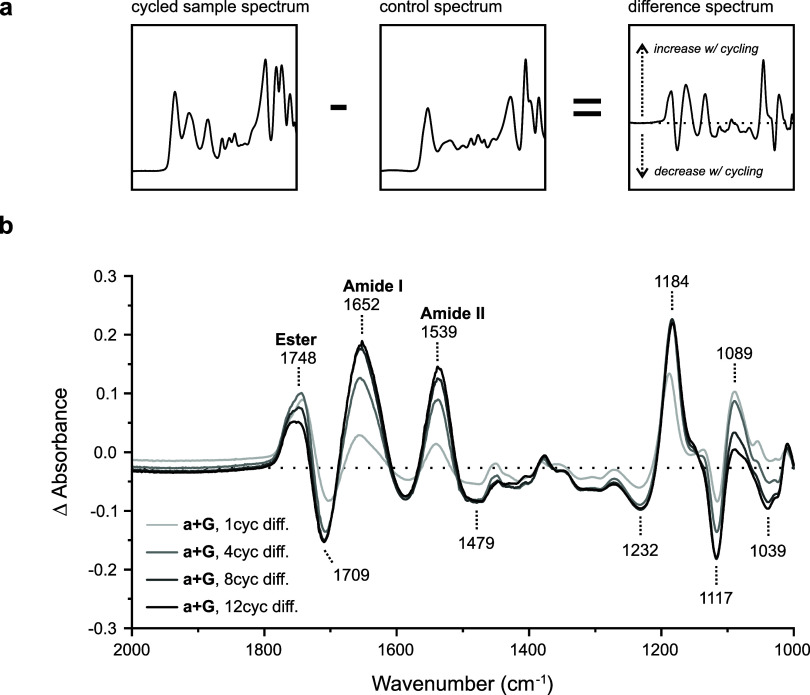
(a) Overview of differential FTIR spectroscopy for proto-biopolymer analysis. Monomer control spectra are subtracted from cycled sample spectra to generate difference spectra. (b) Difference FTIR spectra for lactic acid + glycine (a + G) depsipeptides after 1, 4, 8, and 12 wet–dry cycles in the main fingerprint region. Marked signals correspond to ester (1748 cm^–1^) and amide/peptide (1652 and 1539 cm^–1^) backbone linkages in depsipeptides. Assignments of labeled signals are provided in Table 1. No signal normalization was used.

The differential FTIR spectroscopy method is shown in Figure 2. It consists of subtracting a monomer control spectrum from a polymer spectrum; positive bands are due to functional group growth, and negative bands are due to functional group depletion (Figure 2a). In Figure 2b, differential spectra of lactic acid and glycine condensation products are shown after 1, 4, 8, and 12 wet–dry cycles. Positive signals at 1748 cm^–1^ (ester), 1652 cm^–1^ (amide I), and 1539 cm^–1^ (amide II) are unambiguously assigned to backbone linkages in depsipeptides. Raw FTIR spectra are provided in the Supporting Information (Figures S1–S5, SI).

Assignments of major signals in the fingerprint region from Figure 2b are provided in Table 1. Carbonyl assignments (ester, amide I/II, carboxylic acid) are of highest confidence. Other assignments are supported by additional control experiments (Figures S6–S7, SI) and pertinent references.^59−63^ Zoomed-in spectra from Figure 2b between 1250 and 1050 cm^–1^ are provided in the Supporting Information, along with additional discussion (Figure S8, SI).

**Table 1:** FTIR Bands Shown in Figure 2b^a^

signal (cm^–1^)	group	assignment/comments
1748	C=O str. (ester)	ester linkages in depsipeptide and oligoester (polymer)
1709	C=O str. (carb. acid)	C=O of free glycine and/or lactic acid (monomers)
1652	amide I (C=O str.)	backbone amide linkages in depsipeptide (polymer)
1539	amide II (N–H bend)	backbone amide linkages in depsipeptide (polymer)
1479	–CH_3_ bend	side chain on lactic acid (both monomer and polymer)
1232	mixed C–O/C–N str.	carboxylic acid (monomers) and/or 2° amide (polymer)
1184	C–O str.	ester linkage, C–C–O (polymer)
1117	C–O str.	free lactic acid (monomer)
1089	C–O str.	ester linkage, O–C–C (polymer)
1039	C–CH_3_ str.	lactic acid (both; also dependent on ATR film thickness^78^)

a Assignments of bands between 1300 and 1000 cm^–1^ are tentative.

**Figure 3:**
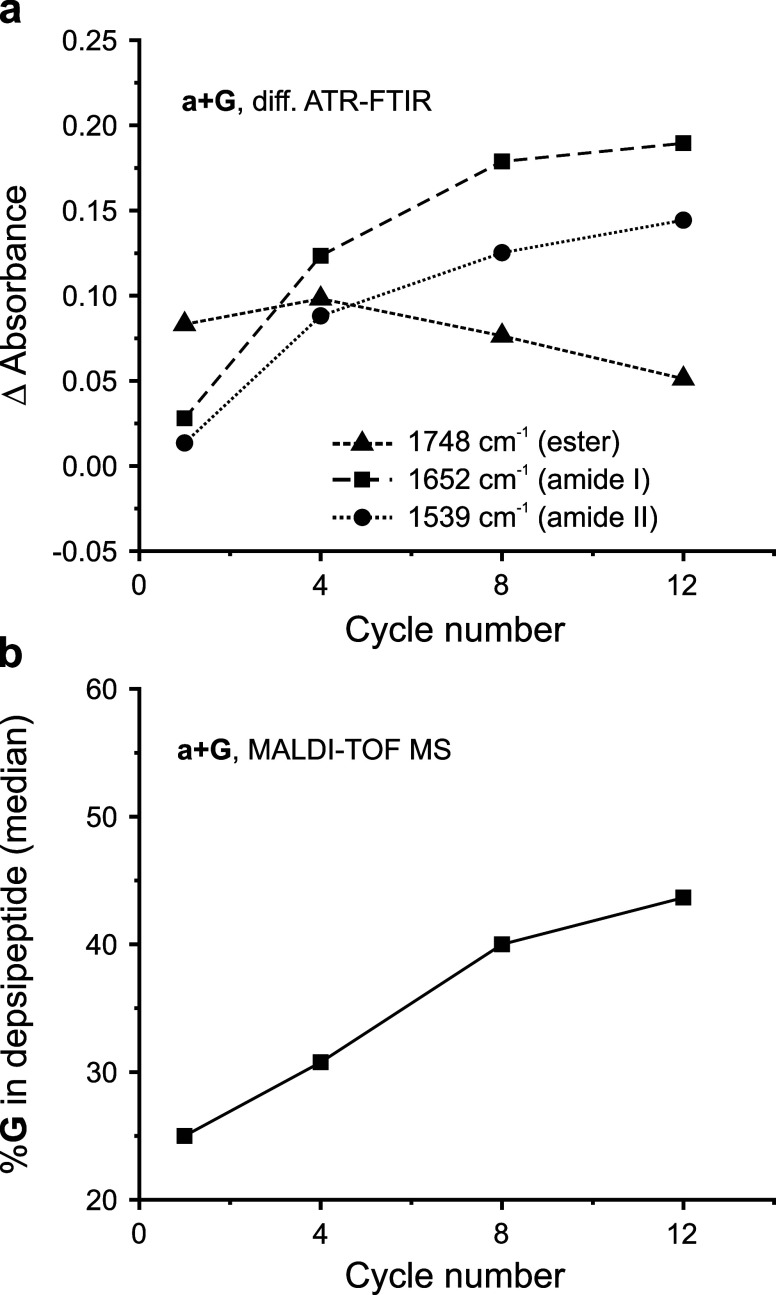
Key ester/amide changes with wet–dry cycling and comparison to MALDI-TOF MS, an orthogonal technique. (a) Differential FTIR ester and amide signals from Figure 2b. Amide I (1652 cm^–1^) and amide II (1539 cm^–1^) bands increase with cycling, whereas the ester C=O band (1748 cm^–1^) increases to 4 cycles and then decreases. These data are consistent with amide bond formation via activated ester intermediates. (b) Comparison to MALDI-TOF MS, an orthogonal technique, for the same a + G depsipeptide samples. The median glycine content in all depsipeptides increases from 1 cycle to 12 cycles, consistent with differential FTIR spectroscopy.

Changes to the ester and amide I/II signals of depsipeptides over cycling provide direct insight into the condensation process. Differential absorbances at 1748, 1652, and 1539 cm^–1^ from Figure 2b are graphed against cycle number in Figure 3a. Amide I/II bands increase to 12 cycles, whereas the ester band increases to 4 cycles and then decreases. These data support the model of depsipeptide condensation by ester activation and ester–amide exchange.^12^ With additional cycling, ester linkages of hydroxy acids are displaced by more stable amide/peptide linkages of amino acids. Ester linkages are also more susceptible to hydrolysis during wet phases of cycling.^11^ As a result, depsipeptides become more peptide-rich over time.

Differential FTIR analysis of lactic acid and glycine depsipeptides was validated by an orthogonal technique, MALDI-TOF MS, in Figure 3b. Characterization of the same samples showed the median amino acid content in depsipeptides increased from 25.0% after 1 cycle to 43.7% after 12 cycles (mass spectra in Figures S9–S12, SI). Amide I/II signals in Figure 3a track similarly to MALDI data over cycling in Figure 3b, with some difference in slope after 4 cycles. While lacking the specificity of MS, differential FTIR provides clear and compelling evidence of proto-biopolymer condensation without the need for expensive instrumentation.

### Practical Considerations: Controls, Data Acquisition, and Normalization

Control samples for differential FTIR contained all monomers at the same initial concentration and solution volume (0.200 mL) as cycled samples. These were left to dry at RT with cap open until sufficient water evaporation—typically less than 7 days for 1.5 mL microcentrifuge tubes. Ceramic spot plates have a higher surface area-to-volume ratio and can be used to reduce evaporation times for controls. While a systematic investigation of surface area-to-volume and wet–dry cycling is outside of the scope of this manuscript, a recent study suggests this parameter may influence product formation in concert with reaction kinetics and cycle turnover.^64^

The broad O–H stretch around 3300 cm^–1^ was typically stronger in control samples than in wet–dry cycled samples due to less water evaporation; cycled samples were dried at 85 °C, whereas controls were dried at RT to minimize polymerization. Uncycled controls had a gel-like appearance, whereas wet–dry cycled products were harder amorphous solids which required more scratching to remove from the reaction vessel. Modest ester signal was observed in the lactic acid and glycine control sample, and no amide/peptide bond signal (Figure S1, SI).

For differential FTIR, all spectra were acquired in absorbance mode as transmittance is not linearly proportional to concentration via Beer’s law.^59^ It is recommended that absorbance values of 0.70 or less be used for spectral subtraction, as this corresponds to ∼20% light transmission.

Normalization can be performed to improve spectral quality if/when inconsistent amounts of sample material are added to ATR target. When normalization was used, cycled samples were scaled to their controls before subtraction, according to eq 1.
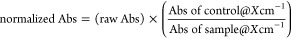
1

The scaling factor in eq 1 was the ratio of absorbances at a chosen wavenumber, *X* cm^–1^. The function generated at the normalization wavenumber was applied to the entire data set before subtraction to generate normalized difference spectra.

**Figure 4:**
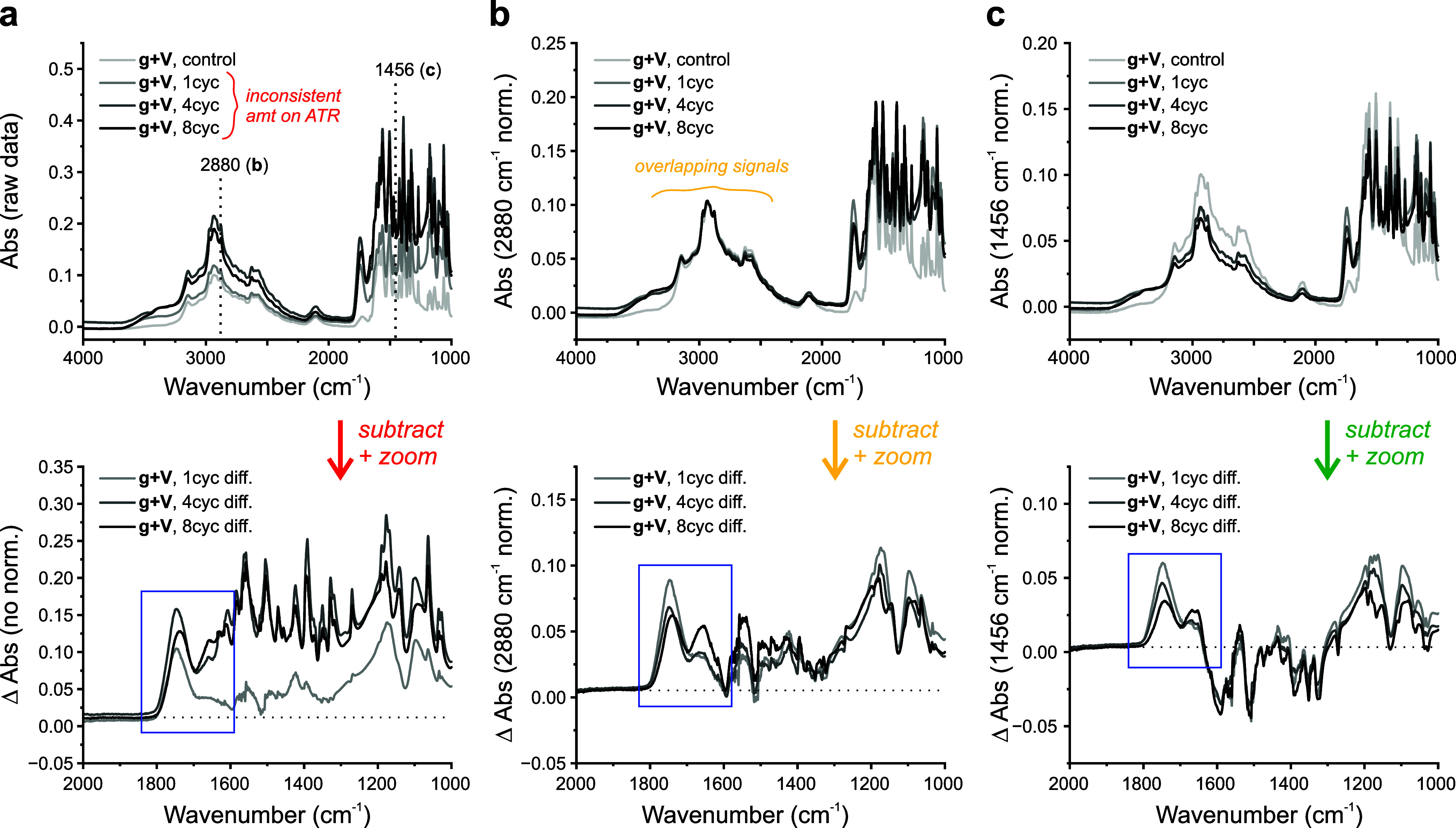
Normalization examples for glycolic acid and valine (g + V) depsipeptides. (a) Raw FTIR spectra without normalization and resulting difference spectra. Inconsistent amounts of sample placed on ATR target resulted in baseline drift across the fingerprint region, including in the key ester/amide I region for depsipeptides (blue box). (b) FTIR spectra after normalization at 2880 cm^–1^ (valine methine C–H str.) and resulting difference spectra. The ester/amide I region is improved, but few negative signals are observed (a.k.a. depletion with cycling). This is likely due to overlapping O–H str. at the normalization value and varying amounts of water between control and cycled samples. (c) FTIR spectra after normalization at 1456 cm^–1^ (assigned to asymmetric CH_3_ bend) and resulting difference spectra. Ester/amide I region is clean, and both positive and negative changes are observed with cycling.

An example of when to normalize is shown in Figure 4. Here, imprecise amounts of glycolic acid and valine depsipeptide samples were added to the ATR target and their spectra were obtained, along with their monomer control. Without normalization (Figure 4a), 4-cycle and 8-cycle difference spectra exhibited baseline drift and cannot be easily compared to the 1-cycle sample. In Figure 4b, cycled samples were normalized to the control at 2880 cm^–1^ (assigned to the methine C–H stretch of valine). The quality of difference spectra was improved; however, some baseline drift remained as seen in the lack of depletion signals. At 2880 cm^–1^, the C–H band overlaps with the broad O–H band from water, and water content fluctuates from sample to sample. In Figure 4c, cycled samples were normalized to the control sample at 1456 cm^–1^ (assigned to the asymmetric CH_3_ bend of valine), and baseline drift in difference spectra was minimized. Ester and amide changes can be clearly observed (blue inset).

To summarize, normalization can improve differential FTIR spectral quality before spectral subtraction, and the normalization wavenumber should be chosen with care. An ideal normalization wavenumber is specific to one type of vibration/functional group which is chemically stable during condensation.

### Application: Polymerization in the Presence of Space Dust Simulants

**Figure 5:**
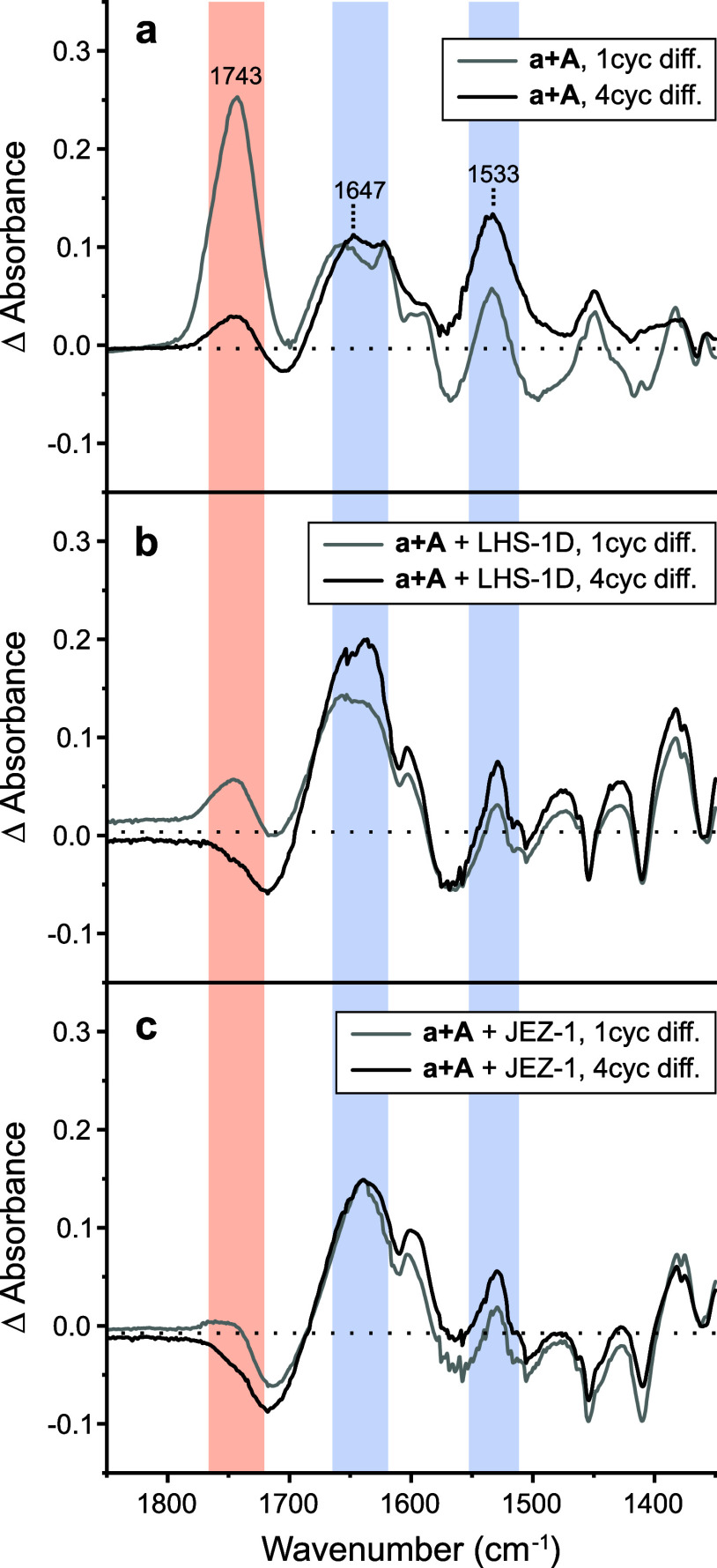
Differential FTIR spectra of lactic acid and alanine (a + A) depsipeptides subjected to wet–dry cycling in the absence or presence of model dust simulants. (a) FTIR spectra without model dust simulant added. Ester formation (light red) is pronounced at 1 cycle and conversion to amide/peptide (light blue) is observed at 4 cycles. With the addition of (b) 1.0 mg of LHS-1D lunar dust simulant or (c) 1.0 mg of JEZ-1 Martian dust simulant to samples, spectra show reduced esterification, yet amide/peptide formation persists.

Differential FTIR was used to investigate the effects of space dust simulants on lactic acid and alanine (a + A) polymerization without sample pretreatment (Figure 5). Two simulants were selected, LHS-1D model lunar dust and JEZ-1 model Martian dust. Both LHS-1D and JEZ-1 contain silica and other oxidized materials. All differential FTIR spectra in Figure 5 were normalized to the uncycled control at 1362 cm^–1^ (assigned to CH_3_ symmetric bend of alanine).

Without space dust simulant, ester and amide I/II signals of lactic acid and alanine (a + A) depsipeptides were present after 4 wet–dry cycles (Figure 5a). The ester signal was quite intense after 1 cycle and then depleted after 4 cycles. When 1.0 mg of LHS-1D model lunar dust was added to a + A samples subjected to cycling, both ester and amide I/II signals were observed, yet the initial ester signal was much lower (Figure 5b). Similarly, when 1.0 mg JEZ-1 model Martian dust was added to a + A samples subjected to 4 wet–dry cycles, amide I/II signals were present, but ester signal was suppressed (Figure 5c). Prior work from McKee et al. demonstrated that depsipeptides formed by wet–dry cycling in the presence of silica had lower ester content.^65^ Our differential FTIR spectra suggest that depsipeptides formed in the presence of simulated space dust have lower ester content also, particularly after the first cycle.

MALDI was performed on these samples also, with centrifugation (3000 rpm for 3 min in 50% acetonitrile) to reduce dust effects on MS analysis. Even with centrifugation, MALDI spectra of dust samples had reduced S/N and spectral quality (see spectra and MSPolyCalc reports in the Supporting Information). It is possible that some oligomers were removed along with dusts during sample preparation, making it difficult to corroborate amino acid content in these samples (Table S1, SI). Depsipeptides in JEZ-1 or LHS-1D samples that were detected by MALDI were generally short and had less combinatorial diversity than those without dust present (Table S1, SI).

### Current Limitations and Future Directions

As mentioned previously, differential FTIR spectroscopy is a bulk analysis. Differential ester and/or amide signals are reflective of the composition of all proto-peptide oligomers in a sample. If a more specific technique such as MS is not available, it is conceivable to fractionate by HPLC and couple with FTIR^66^ to investigate compositions of different oligomer lengths. Longer depsipeptides are typically more hydrophobic and retain for longer on reversed-phase columns.^19,21^ A hybrid approach may also be useful for discriminating ester-and-amide depsipeptides from mixtures of peptides and pure oligoesters, perhaps in conjunction with hydrolysis treatment.^68^

More investigation is needed to use differential FTIR for quantitative analysis of oligomers in model prebiotic reactions and complex sample mixtures; this study is a proof-of-concept focused on qualitative analysis. Here, we compared oligomer growth patterns to an orthogonal technique, MALDI-TOF MS. MALDI was used to determine the relative compositions of hydroxy acid (ester) and amino acid (amide) content in depsipeptides but was unable to quantify total amino acid conversion (i.e., overall amino acid uptake into polymer) as MS ionization efficiencies change with oligomer lengths. MALDI was also hindered by the presence of minerals and salts in simulated space dust. In previous prebiotic studies on depsipeptides, amino acid conversion was typically determined by NMR.^12,28,69^ Like MS, NMR analysis can be difficult in complex sample matrices. With further investigation, it is hypothesized that differential FTIR may be capable of relative quantitation of ester/amide ratios via analysis of standards with known ester and amide content, even in complex matrices. Absolute quantitation of amino acid conversion into polymer would likely necessitate both the use of internal standards^70,71^ and NMR-based validation.

## Conclusions

In this work, a simple and low-cost differential FTIR method was introduced for qualitative monitoring of depsipeptide condensation over wet–dry cycling. Considerations for proper controls, spectral subtraction, and normalization were examined, and the method was shown to tolerate complex sample matrices such as extraterrestrial dust simulants. Future optimization of this method may focus on quantitative analysis, other sample matrices relevant to origins and astrobiology (chemical gardens,^72,73^ model icy/ocean worlds,^74−76^ etc.), and expanding its utility to other classes of proto-biomolecules (e.g., proto-nucleic acids^23,77^).

## Supporting Information Available

Control FTIR spectra, MALDI-TOF MS spectra and tables, and MSPolyCalc MALDI-TOF MS data reports used to calculate median ester and amide content in depsipeptides (PDF)
