# Supplementary material for: Qualitative Monitoring of Proto-Peptide Condensation by Differential FTIR Spectroscopy
Source: ACS Earth Space Chem. 2024 May 6;8(5):937–44. doi: 10.1021/acsearthspacechem.3c00257 (PMC11103710; doi:10.1021/acsearthspacechem.3c00257)
Supplement: Supplementary file 1 — sp3c00257_si_001.pdf [file sp3c00257_si_001.pdf]

Supplemental Information

Qualitative monitoring of proto-peptide condensation by differential FTIR spectroscopy

Keon Rezaeerod,[1] Hanna Heinzmann,[1,2] Alexis V. Torrence,[1] Jui Patel,[1] and Jay G. Forsythe[1]*

(1) Department of Chemistry and Biochemistry, College of Charleston, Charleston, SC 29424 USA (2) Analytical and Bioanalytical Chemistry, Aalen University, 73430 Aalen, Germany

*Corresponding author:
Jay G. Forsythe, Ph.D.
Associate Professor
Department of Chemistry and Biochemistry
College of Charleston
66 George Street
Charleston, SC 29424 USA

Email: forsythejg@cofc.edu
Phone: (+1) 843-953-5052

Keywords

FTIR, wet-dry cycling, depsipeptides, prebiotic chemistry, astrobiology

Abstract

Condensation processes such as wet-dry cycling are thought to have played significant roles in the emergence of proto-peptides. Here, we describe a simple and low-cost method, differential Fourier transform infrared (FTIR) spectroscopy, for qualitative analysis of peptide condensation products in model primordial reactions. We optimize differential FTIR for depsipeptides and apply this method to investigate their polymerization in the presence of extraterrestrial dust simulants.

Table of Contents

Figure	*Page(s)*
**Figure S1**. FTIR spectrum of lactic acid and glycine monomer control.	S3
**Figure S2**. FTIR spectrum of lactic acid and glycine after 1 cycle.	S3
**Figure S3**. FTIR spectrum of lactic acid and glycine after 4 cycles.	S4
**Figure S4**. FTIR spectrum of lactic acid and glycine after 8 cycles.	S4
**Figure S5**. FTIR spectrum of lactic acid and glycine after 12 cycles.	S5
**Figure S6**. FTIR spectrum of lactic acid after 4 cycles.	S5
**Figure S7**. FTIR spectrum of glycine after 4 cycles.	S6
**Figure S8**. Zoomed-in differential FTIR spectra from **Fig. 2b** in main text.	S6
**Figure S9**. MALDI spectrum of lactic acid and glycine after 1 cycle.	S7
**Figure S10**. MALDI spectrum of lactic acid and glycine after 4 cycles.	S7
**Figure S11**. MALDI spectrum of lactic acid and glycine after 8 cycles.	S8
**Figure S12**. MALDI spectrum of lactic acid and glycine after 12 cycles.	S8
**Table S1**. Summary of MALDI data for dust simulant samples.	S9
**Appendix**. MSPolyCalc data reports for all MALDI mass spectra.	S10-S84

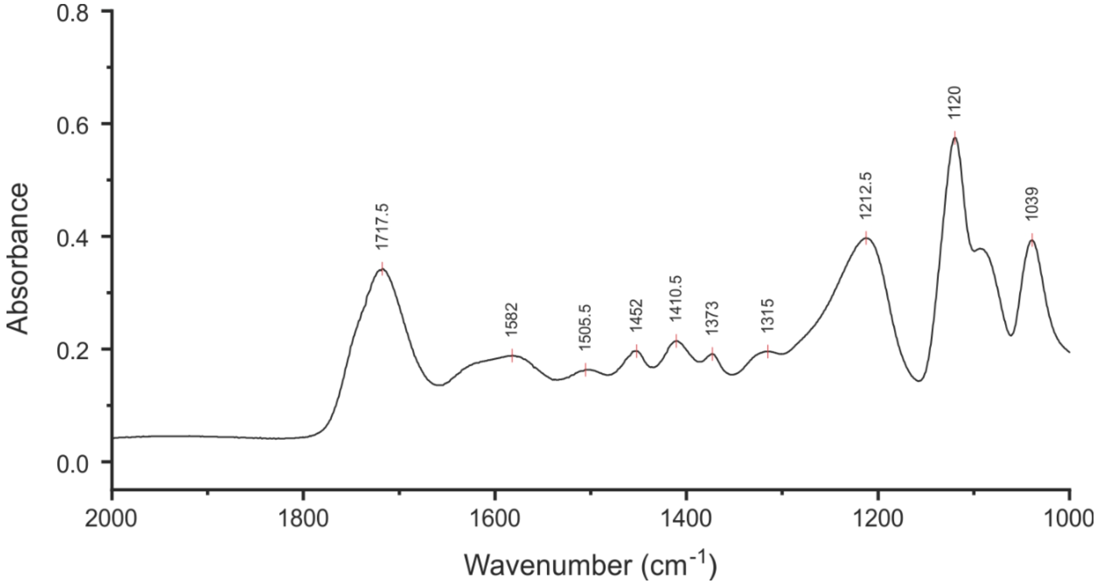

Figure S1. ATR-FTIR spectrum of lactic acid and glycine monomer control in the fingerprint range. The broad carbonyl signal at 1717.5 cm^{-1} is primarily carboxylic acid but also contains some ester. Amide I/II signals are not observed.

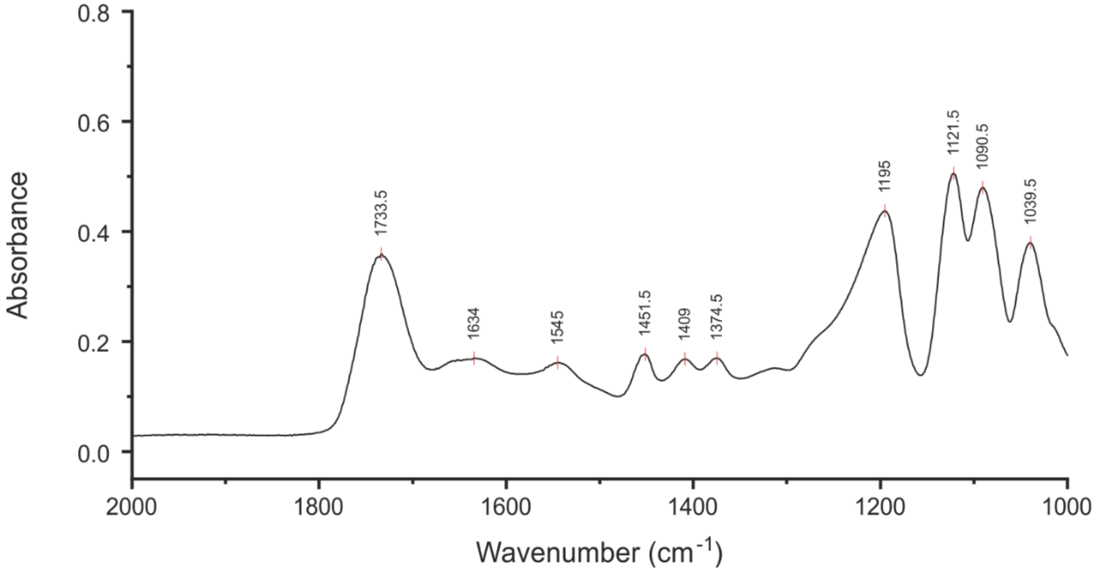

Figure S2. ATR-FTIR spectrum of lactic acid and glycine condensation products after 1 wet-dry cycle in the fingerprint range.

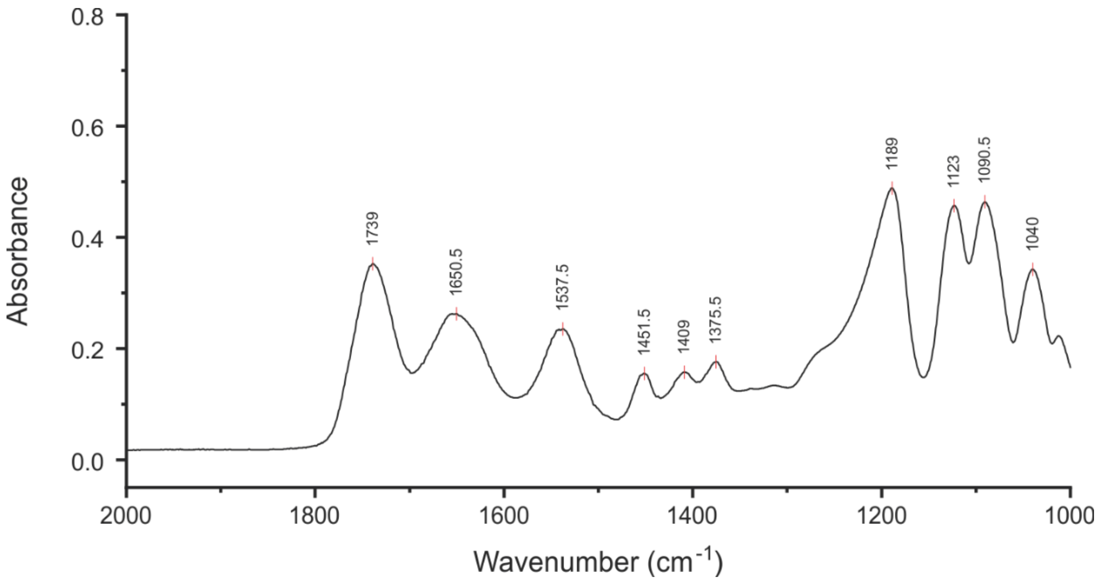

Figure S3. ATR-FTIR spectrum of lactic acid and glycine condensation products after 4 wet-dry cycles in the fingerprint range.

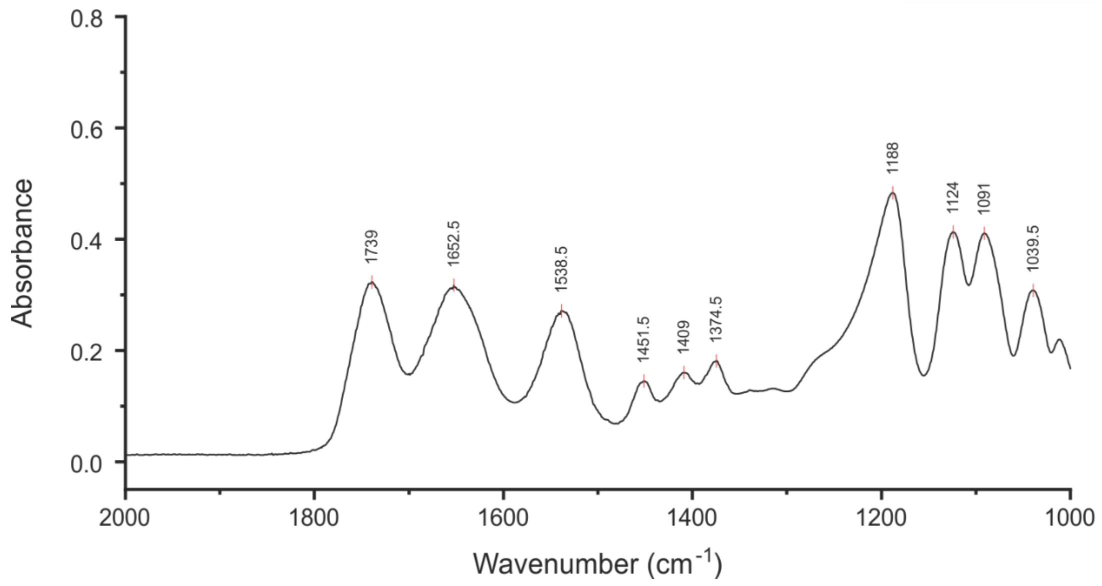

Figure S4. ATR-FTIR spectrum of lactic acid and glycine (a+G) condensation products after 8 wet-dry cycles in the fingerprint range.

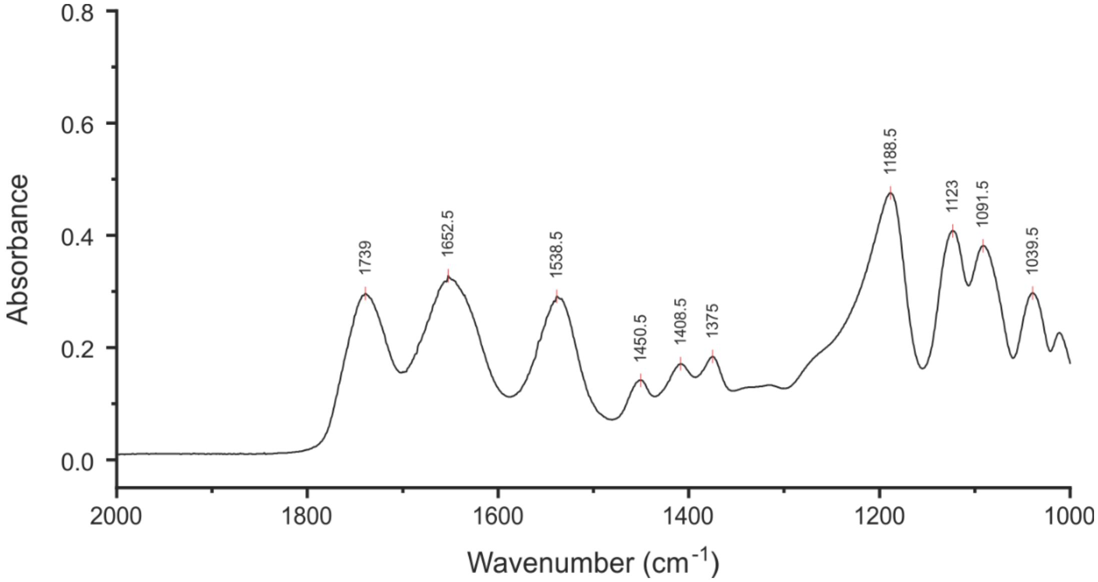

Figure S5. ATR-FTIR spectrum of lactic acid and glycine (a+G) condensation products after 12 wet-dry cycles in the fingerprint range.

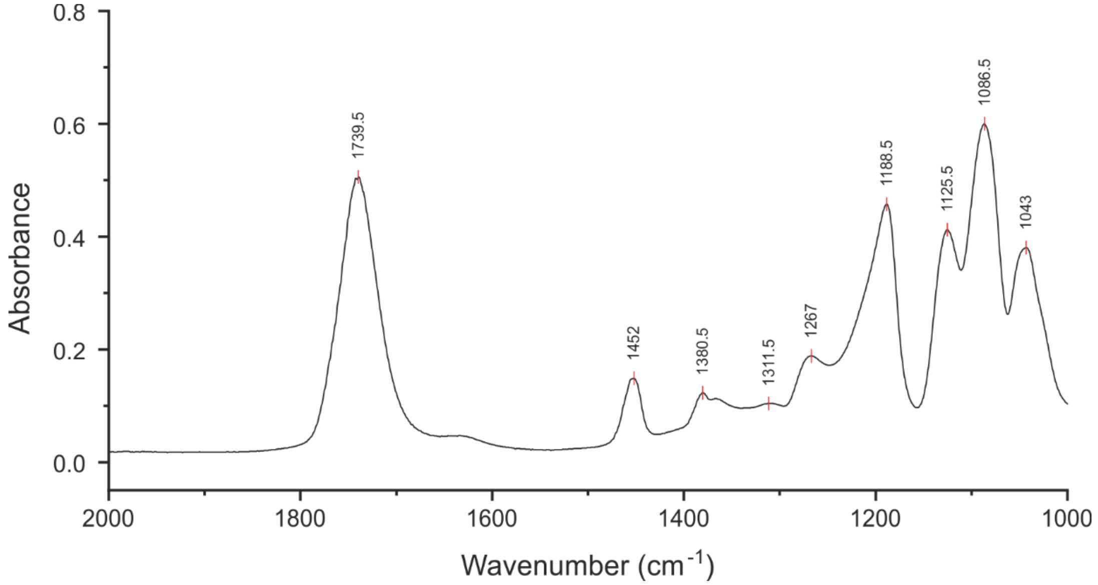

Figure S6. ATR-FTIR control spectrum of lactic acid monomer subjected to 4 wet-dry cycles in the fingerprint range. Intense ester signals are observed, signifying oligoester formation.

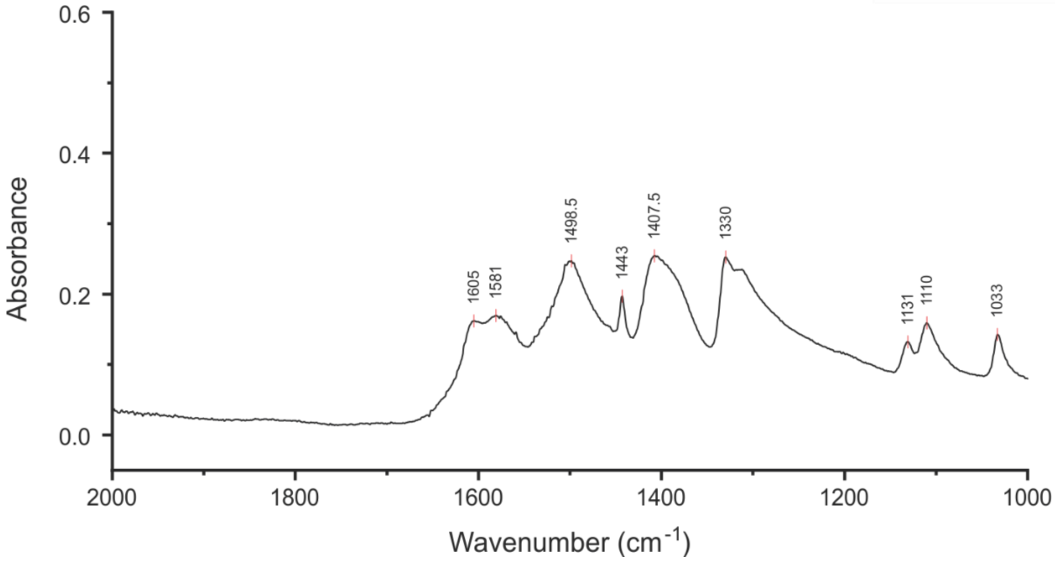

Figure S7. ATR-FTIR control spectrum of glycine subjected to 4 wet-dry cycles in the fingerprint range. Amide I/II signals from peptide bonds are not observed.

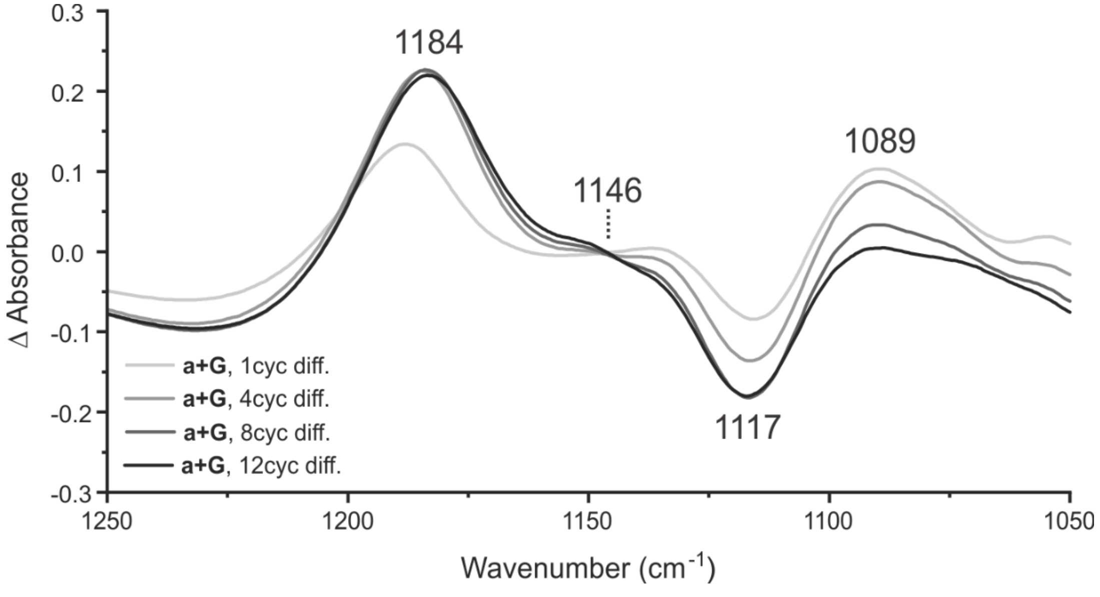

Figure S8. Zoomed-in differential FTIR spectra from **Fig. 2b**. Bands at 1184 cm^{-1} and 1089 cm^{-1} are both assigned as ester C-O stretches, and the depleting band at 1117 cm^{-1} is assigned to the lactic acid monomer C-O stretch. At 1146 cm^{-1}, an isosbestic point is present. Unlike the ester band at 1089 cm^{-1}, the ester band at 1184 cm^{-1} does not grow and then deplete over time. The 1184 cm^{-1} band is assigned to a C-C-O vibration which may be amplified in the amorphous solid/gel phase due to trapped water and resulting hydrogen bonding, whereas the 1089 cm^{-1} is assigned to the O-C-C stretch after the carbonyl group (see Smith ref. 59 in main text).

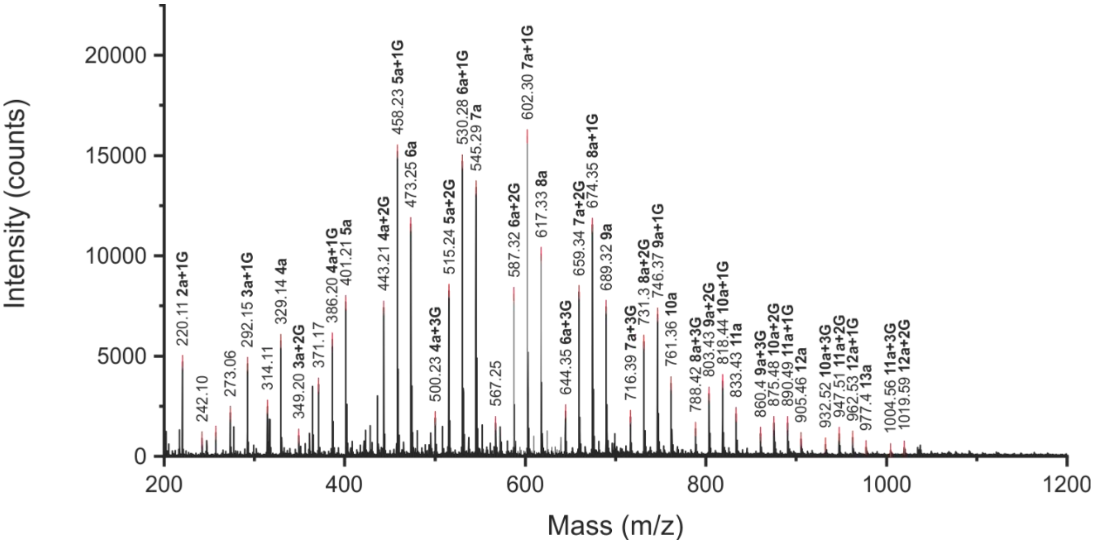

Figure S9. MALDI-TOF mass spectrum of lactic acid and glycine (a+G) depsipeptides and oligoesters after 1 wet-dry cycle. MSPolyCalc report is provided in the Appendix.

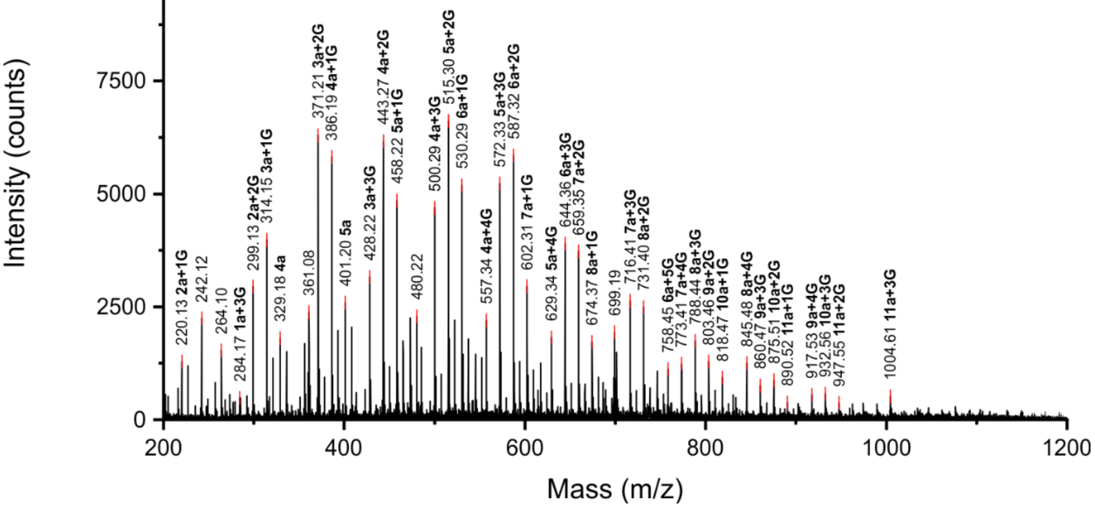

Figure S10. MALDI-TOF mass spectrum of lactic acid and glycine (a+G) depsipeptides and oligoesters after 4 wet-dry cycles. MSPolyCalc report is provided in the Appendix.

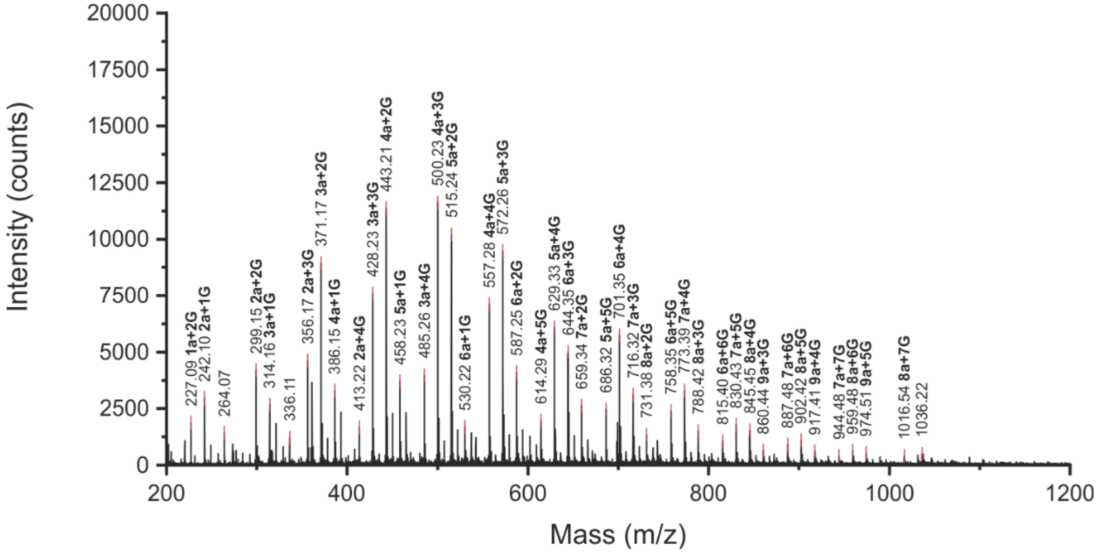

Figure S11. MALDI-TOF mass spectrum of lactic acid and glycine (a+G) depsipeptides after 8 wet-dry cycles. MSPolyCalc report is provided in the Appendix.

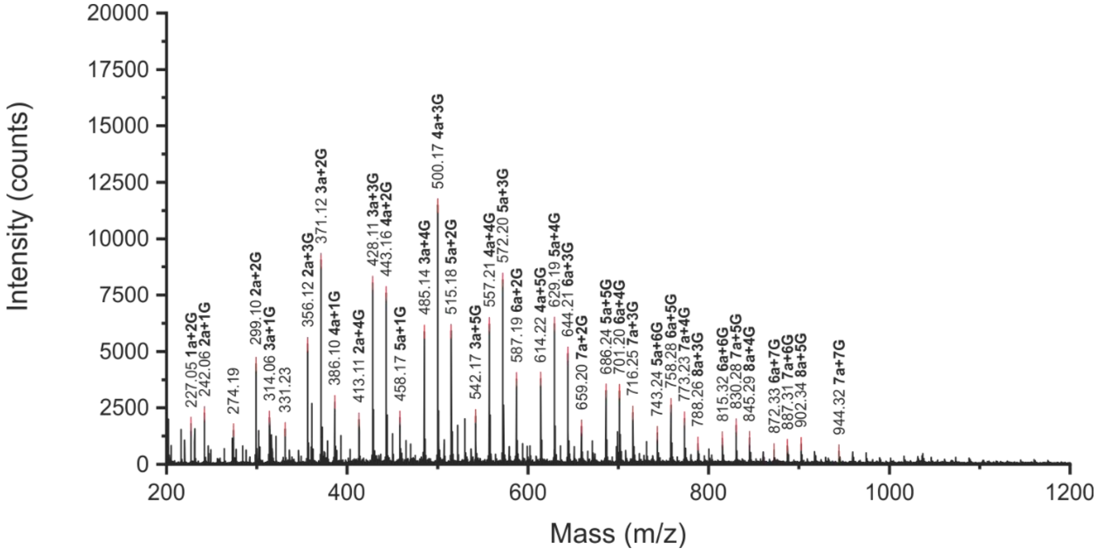

Figure S12. MALDI-TOF mass spectrum of lactic acid and glycine (a+G) depsipeptides after 12 wet-dry cycles. MSPolyCalc report is provided in the Appendix.

Table S1. MALDI-TOF MS data summary for lactic acid and alanine depsipeptides after 4 wet-dry cycles, in the absence and presence of space dust simulants. Experimental MALDI spectra and data (both 1 and 4 cycle) are provided in the Appendix, which include raw spectra. Spectral quality decreased when dusts were present, making it difficult to compare between MALDI and FTIR. Nevertheless, the data suggest depsipeptides formed in the presence of dust may be shorter and have less compositional diversity.

	Lacking dust simulant	1.0 mg LHS-1D added	1.0 mg JEZ-1 added
Longest depsipeptide detected (# of residues)	16	7	7
Median amino acid composition in detected depsipeptides (%)	40.0	36.7	46.4
Unique combinations detected (*n* hydroxy acid + *m* amino acid)	79	13	20

Analytical report

Page under construction...

Analysis parameters

aG MALDI, 1cyc

Ionizations

MF	Monoisotopic mass	m/z mass
Na^+	22.98977	22.98922
K^+	38.96371	38.96316
H^+	1.00783	1.00728

End groups

α	ω	Color
H	OH	

Monomers

	Description	mf	Monoisotopic mass	min	max
A	lactic acid	$C_3H_4O_2$	72.02113	0	100
B	glycine	C_2H_3NO	57.02146	0	100

Experimental spectrum

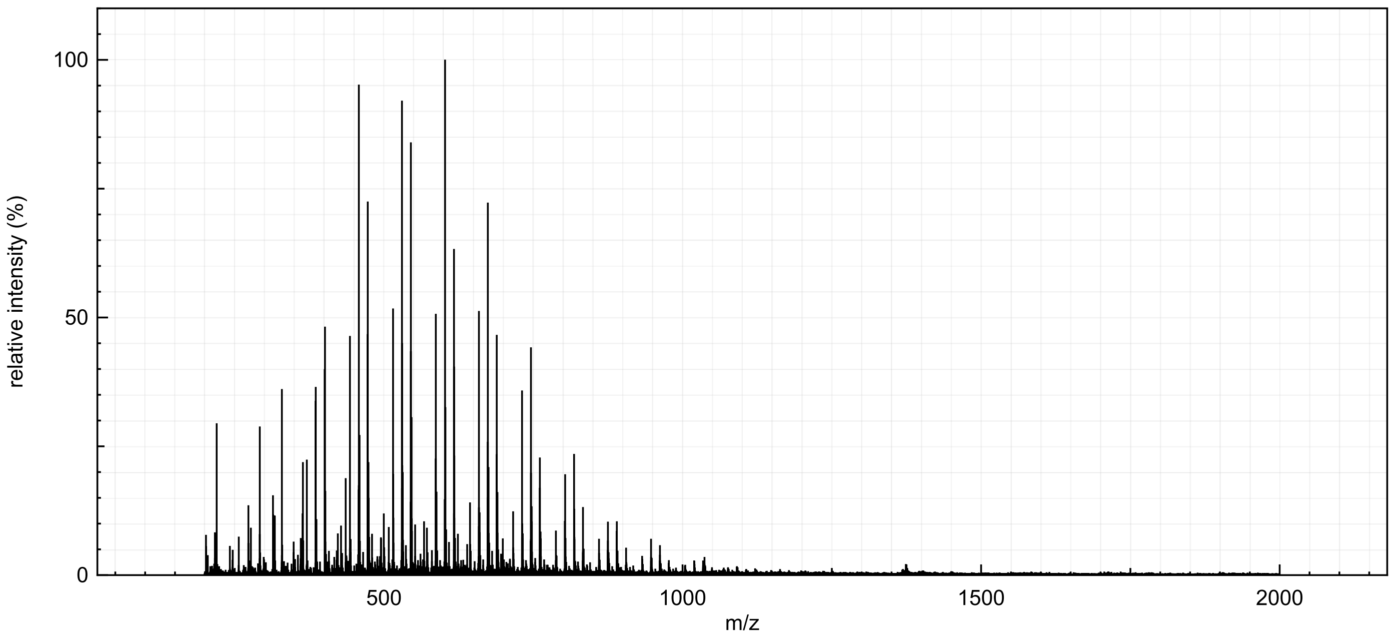

Reconstructed spectrum

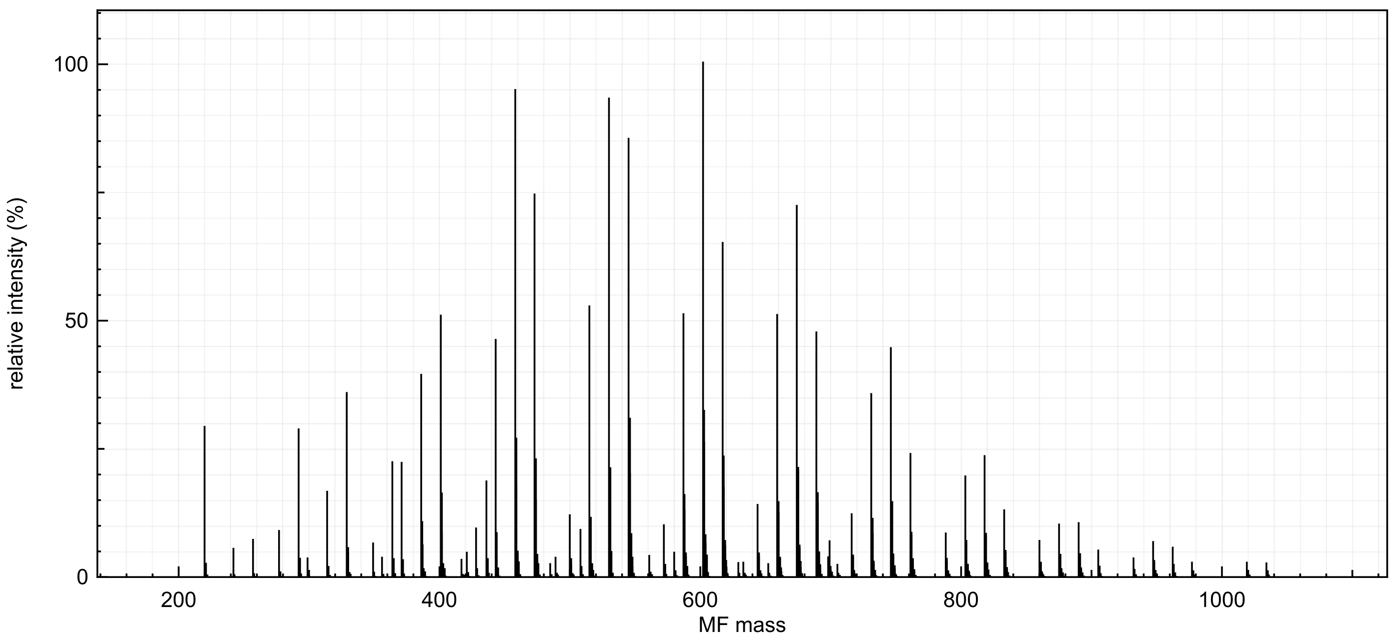

Error (ppm) versus m/z

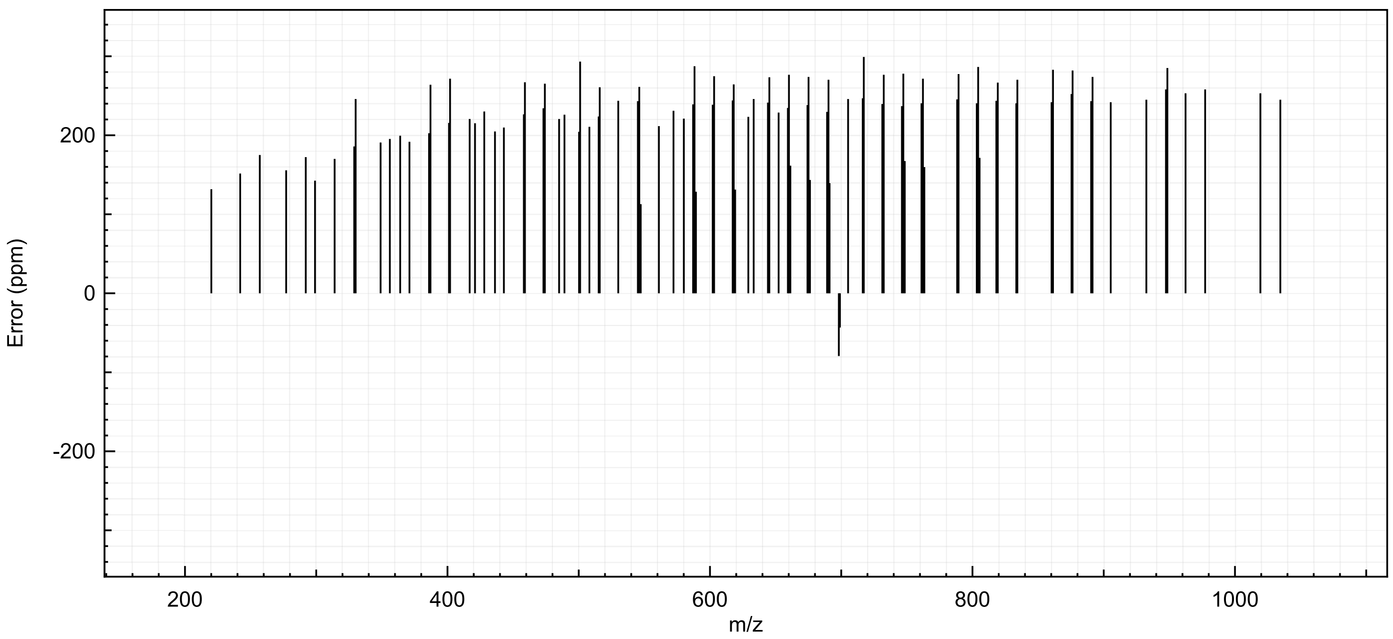

Analysis result

Percent of peaks covered: 66.82%
Number of assigned peaks: 98

Polymers [m/z]

Composition	Nb units	MF	Ioniz.	em [m/z]	Charge	Similarity	Quantity
A: 2 B: 1	3	$C_8H_{13}NO_6$	H^+	220.08156	1	80.98%	0.56%
A: 2 B: 1	3	$C_8H_{13}NO_6$	Na^+	242.06351	1	55.35%	0.21%
A: 3 B: 0	3	$C_9H_{14}O_7$	Na^+	257.06317	1	66.74%	0.19%
A: 2 B: 2	4	$C_{10}H_{16}N_2O_7$	H^+	277.10303	1	59.95%	0.25%
A: 2 B: 2	4	$C_{10}H_{16}N_2O_7$	Na^+	299.08497	1	51.99%	0.21%
A: 3 B: 1	4	$C_{11}H_{17}NO_8$	H^+	292.10269	1	79.84%	0.62%
A: 3 B: 1	4	$C_{11}H_{17}NO_8$	K^+	330.05857	1	57.74%	0.29%
A: 3 B: 1	4	$C_{11}H_{17}NO_8$	Na^+	314.08464	1	41.40%	0.93%
A: 4 B: 0	4	$C_{12}H_{18}O_9$	Na^+	329.08430	1	78.00%	0.70%
A: 2 B: 3	5	$C_{12}H_{19}N_3O_8$	Na^+	356.10644	1	46.02%	0.20%
A: 3 B: 2	5	$C_{13}H_{20}N_2O_9$	H^+	349.12416	1	59.97%	0.30%
A: 3 B: 2	5	$C_{13}H_{20}N_2O_9$	Na^+	371.10610	1	77.46%	0.45%
A: 3 B: 2	5	$C_{13}H_{20}N_2O_9$	K^+	387.08004	1	66.31%	0.42%
A: 4 B: 1	5	$C_{14}H_{21}NO_{10}$	H^+	364.12382	1	76.06%	0.65%
A: 4 B: 1	5	$C_{14}H_{21}NO_{10}$	Na^+	386.10577	1	83.56%	1.14%
A: 4 B: 1	5	$C_{14}H_{21}NO_{10}$	K^+	402.07970	1	70.97%	0.60%
A: 5 B: 0	5	$C_{15}H_{22}O_{11}$	Na^+	401.10543	1	82.15%	1.50%
A: 5 B: 0	5	$C_{15}H_{22}O_{11}$	K^+	417.07937	1	45.94%	0.29%
A: 3 B: 3	6	$C_{15}H_{23}N_3O_{10}$	Na^+	428.12756	1	64.12%	0.43%
A: 4 B: 2	6	$C_{16}H_{24}N_2O_{11}$	H^+	421.14529	1	42.32%	0.39%
A: 4 B: 2	6	$C_{16}H_{24}N_2O_{11}$	Na^+	443.12723	1	86.05%	0.87%
A: 4 B: 2	6	$C_{16}H_{24}N_2O_{11}$	K^+	459.10117	1	76.12%	0.76%
A: 5 B: 1	6	$C_{17}H_{25}NO_{12}$	H^+	436.14495	1	65.66%	0.57%
A: 5 B: 1	6	$C_{17}H_{25}NO_{12}$	Na^+	458.12690	1	85.48%	1.99%
A: 5 B: 1	6	$C_{17}H_{25}NO_{12}$	K^+	474.10083	1	73.20%	0.97%
A: 6 B: 0	6	$C_{18}H_{26}O_{13}$	Na^+	473.12656	1	81.92%	2.16%
A: 6 B: 0	6	$C_{18}H_{26}O_{13}$	K^+	489.10050	1	52.26%	0.33%
A: 3 B: 4	7	$C_{17}H_{26}N_4O_{11}$	Na^+	485.14903	1	53.42%	0.36%
A: 3 B: 4	7	$C_{17}H_{26}N_4O_{11}$	K^+	501.12297	1	61.96%	0.23%

Composition	Nb units	MF	Ioniz.	em [m/z]	Charge	Similarity	Quantity
A: 4 B: 3	7	$C_{18}H_{27}N_3O_{12}$	Na^+	500.14869	1	78.18%	0.45%
A: 4 B: 3	7	$C_{18}H_{27}N_3O_{12}$	K^+	516.12263	1	71.63%	0.51%
A: 5 B: 2	7	$C_{19}H_{28}N_2O_{13}$	Na^+	515.14836	1	85.68%	1.21%
A: 6 B: 1	7	$C_{20}H_{29}NO_{14}$	H^+	508.16608	1	65.28%	0.37%
A: 6 B: 1	7	$C_{20}H_{29}NO_{14}$	Na^+	530.14803	1	87.29%	2.35%
A: 6 B: 1	7	$C_{20}H_{29}NO_{14}$	K^+	546.12196	1	81.53%	0.99%
A: 7 B: 0	7	$C_{21}H_{30}O_{15}$	Na^+	545.14769	1	85.46%	2.25%
A: 7 B: 0	7	$C_{21}H_{30}O_{15}$	K^+	561.12163	1	56.83%	0.33%
A: 1 B: 8	9	$C_{19}H_{30}N_8O_{11}$	H^+	547.21068	1	55.93%	0.53%
A: 5 B: 3	8	$C_{21}H_{31}N_3O_{14}$	Na^+	572.16982	1	75.07%	0.51%
A: 5 B: 3	8	$C_{21}H_{31}N_3O_{14}$	K^+	588.14376	1	78.48%	0.57%
A: 6 B: 2	8	$C_{22}H_{32}N_2O_{15}$	Na^+	587.16949	1	85.80%	1.33%
A: 6 B: 2	8	$C_{22}H_{32}N_2O_{15}$	K^+	603.14343	1	81.79%	1.05%
A: 7 B: 1	8	$C_{23}H_{33}NO_{16}$	H^+	580.18721	1	58.50%	0.32%
A: 7 B: 1	8	$C_{23}H_{33}NO_{16}$	Na^+	602.16915	1	86.24%	2.46%
A: 7 B: 1	8	$C_{23}H_{33}NO_{16}$	K^+	618.14309	1	76.98%	0.96%
A: 0 B: 10	10	$C_{20}H_{32}N_{10}O_{11}$	H^+	589.23248	1	51.24%	0.36%
A: 8 B: 0	8	$C_{24}H_{34}O_{17}$	Na^+	617.16882	1	83.70%	2.00%
A: 8 B: 0	8	$C_{24}H_{34}O_{17}$	K^+	633.14276	1	53.24%	0.33%
A: 5 B: 4	9	$C_{23}H_{34}N_4O_{15}$	Na^+	629.19129	1	60.32%	0.35%
A: 5 B: 4	9	$C_{23}H_{34}N_4O_{15}$	K^+	645.16522	1	67.67%	0.31%
A: 2 B: 8	10	$C_{22}H_{34}N_8O_{13}$	H^+	619.23181	1	55.73%	0.59%
A: 6 B: 3	9	$C_{24}H_{35}N_3O_{16}$	Na^+	644.19095	1	78.14%	0.54%
A: 6 B: 3	9	$C_{24}H_{35}N_3O_{16}$	K^+	660.16489	1	77.25%	0.61%
A: 7 B: 2	9	$C_{25}H_{36}N_2O_{17}$	Na^+	659.19062	1	86.31%	1.29%
A: 7 B: 2	9	$C_{25}H_{36}N_2O_{17}$	K^+	675.16456	1	79.80%	0.88%
A: 8 B: 1	9	$C_{26}H_{37}NO_{18}$	H^+	652.20834	1	54.97%	0.25%
A: 8 B: 1	9	$C_{26}H_{37}NO_{18}$	Na^+	674.19028	1	87.65%	1.93%
A: 8 B: 1	9	$C_{26}H_{37}NO_{18}$	K^+	690.16422	1	77.67%	0.73%
A: 1 B: 10	11	$C_{23}H_{36}N_{10}O_{13}$	H^+	661.25361	1	54.21%	0.40%
A: 1 B: 10	11	$C_{23}H_{36}N_{10}O_{13}$	K^+	699.20949	1	60.20%	0.59%
A: 9 B: 0	9	$C_{27}H_{38}O_{19}$	Na^+	689.18995	1	85.14%	1.43%
A: 9 B: 0	9	$C_{27}H_{38}O_{19}$	K^+	705.16389	1	55.87%	0.35%
A: 2 B: 9	11	$C_{24}H_{37}N_9O_{14}$	H^+	676.25327	1	55.21%	0.51%

Composition	Nb units	MF	Ioniz.	em [m/z]	Charge	Similarity	Quantity
A: 2 B: 9	11	$C_{24}H_{37}N_9O_{14}$	Na^+	698.23522	1	45.71%	0.63%
A: 6 B: 4	10	$C_{26}H_{38}N_4O_{17}$	K^+	717.18635	1	68.43%	0.37%
A: 3 B: 8	11	$C_{25}H_{38}N_8O_{15}$	H^+	691.25294	1	58.57%	0.46%
A: 7 B: 3	10	$C_{27}H_{39}N_3O_{18}$	Na^+	716.21208	1	74.48%	0.55%
A: 7 B: 3	10	$C_{27}H_{39}N_3O_{18}$	K^+	732.18602	1	67.40%	0.59%
A: 8 B: 2	10	$C_{28}H_{40}N_2O_{19}$	Na^+	731.21175	1	83.49%	1.07%
A: 8 B: 2	10	$C_{28}H_{40}N_2O_{19}$	K^+	747.18568	1	79.00%	0.72%
A: 9 B: 1	10	$C_{29}H_{41}NO_{20}$	Na^+	746.21141	1	85.55%	1.43%
A: 9 B: 1	10	$C_{29}H_{41}NO_{20}$	K^+	762.18535	1	75.61%	0.55%
A: 10 B: 0	10	$C_{30}H_{42}O_{21}$	Na^+	761.21108	1	81.59%	0.97%
A: 3 B: 9	12	$C_{27}H_{41}N_9O_{16}$	H^+	748.27440	1	58.43%	0.45%
A: 7 B: 4	11	$C_{29}H_{42}N_4O_{19}$	K^+	789.20748	1	67.14%	0.35%
A: 4 B: 8	12	$C_{28}H_{42}N_8O_{17}$	H^+	763.27407	1	58.08%	0.37%
A: 8 B: 3	11	$C_{30}H_{43}N_3O_{20}$	Na^+	788.23321	1	68.42%	0.48%
A: 8 B: 3	11	$C_{30}H_{43}N_3O_{20}$	K^+	804.20715	1	76.30%	0.43%
A: 9 B: 2	11	$C_{31}H_{44}N_2O_{21}$	Na^+	803.23288	1	82.35%	0.77%
A: 9 B: 2	11	$C_{31}H_{44}N_2O_{21}$	K^+	819.20681	1	77.90%	0.49%
A: 10 B: 1	11	$C_{32}H_{45}NO_{22}$	Na^+	818.23254	1	84.58%	0.88%
A: 10 B: 1	11	$C_{32}H_{45}NO_{22}$	K^+	834.20648	1	72.21%	0.42%
A: 3 B: 10	13	$C_{29}H_{44}N_{10}O_{17}$	H^+	805.29587	1	55.10%	0.32%
A: 11 B: 0	11	$C_{33}H_{46}O_{23}$	Na^+	833.23221	1	77.15%	0.62%
A: 8 B: 4	12	$C_{32}H_{46}N_4O_{21}$	K^+	861.22861	1	69.41%	0.31%
A: 9 B: 3	12	$C_{33}H_{47}N_3O_{22}$	Na^+	860.25434	1	73.35%	0.44%
A: 9 B: 3	12	$C_{33}H_{47}N_3O_{22}$	K^+	876.22828	1	74.43%	0.38%
A: 10 B: 2	12	$C_{34}H_{48}N_2O_{23}$	Na^+	875.25401	1	77.55%	0.57%
A: 10 B: 2	12	$C_{34}H_{48}N_2O_{23}$	K^+	891.22794	1	69.74%	0.43%
A: 11 B: 1	12	$C_{35}H_{49}NO_{24}$	Na^+	890.25367	1	76.40%	0.63%
A: 12 B: 0	12	$C_{36}H_{50}O_{25}$	Na^+	905.25334	1	66.81%	0.41%
A: 10 B: 3	13	$C_{36}H_{51}N_3O_{24}$	K^+	948.24941	1	73.06%	0.30%
A: 10 B: 3	13	$C_{36}H_{51}N_3O_{24}$	Na^+	932.27547	1	69.90%	0.34%
A: 11 B: 2	13	$C_{37}H_{52}N_2O_{25}$	Na^+	947.27514	1	76.44%	0.41%
A: 12 B: 1	13	$C_{38}H_{53}NO_{26}$	Na^+	962.27480	1	75.35%	0.41%
A: 13 B: 0	13	$C_{39}H_{54}O_{27}$	Na^+	977.27447	1	66.90%	0.28%
A: 12 B: 2	14	$C_{40}H_{56}N_2O_{27}$	Na^+	1019.29627	1	72.11%	0.32%

Composition	Nb units	MF	Ioniz.	em [m/z]	Charge	Similarity	Quantity
A: 13 B: 1	14	$C_{41}H_{57}NO_{28}$	Na^+	1034.29593	1	53.27%	0.47%

Polymers grouped by monoisotopic mass

Composition				Monoisotopic mass	Quantity
A: 2 B: 1	3	$C_8H_{13}NO_6$	H^+	219.0743	0.77%
A: 2 B: 1	3	$C_8H_{13}NO_6$	Na^+		
A: 3 B: 0	3	$C_9H_{14}O_7$	Na^+	234.0740	0.19%
A: 2 B: 2	4	$C_{10}H_{16}N_2O_7$	H^+	276.0958	0.46%
A: 2 B: 2	4	$C_{10}H_{16}N_2O_7$	Na^+		
A: 3 B: 1	4	$C_{11}H_{17}NO_8$	H^+	291.0954	1.84%
A: 3 B: 1	4	$C_{11}H_{17}NO_8$	K^+		
A: 3 B: 1	4	$C_{11}H_{17}NO_8$	Na^+		
A: 4 B: 0	4	$C_{12}H_{18}O_9$	Na^+	306.0951	0.70%
A: 2 B: 3	5	$C_{12}H_{19}N_3O_8$	Na^+	333.1172	0.20%
A: 3 B: 2	5	$C_{13}H_{20}N_2O_9$	Na^+	348.1169	1.17%
A: 3 B: 2	5	$C_{13}H_{20}N_2O_9$	K^+		
A: 3 B: 2	5	$C_{13}H_{20}N_2O_9$	H^+		
A: 4 B: 1	5	$C_{14}H_{21}NO_{10}$	Na^+	363.1165	2.40%
A: 4 B: 1	5	$C_{14}H_{21}NO_{10}$	H^+		
A: 4 B: 1	5	$C_{14}H_{21}NO_{10}$	K^+		
A: 5 B: 0	5	$C_{15}H_{22}O_{11}$	Na^+	378.1162	1.79%
A: 5 B: 0	5	$C_{15}H_{22}O_{11}$	K^+		
A: 3 B: 3	6	$C_{15}H_{23}N_3O_{10}$	Na^+	405.1383	0.43%
A: 4 B: 2	6	$C_{16}H_{24}N_2O_{11}$	Na^+	420.1380	2.02%
A: 4 B: 2	6	$C_{16}H_{24}N_2O_{11}$	K^+		
A: 4 B: 2	6	$C_{16}H_{24}N_2O_{11}$	H^+		
A: 5 B: 1	6	$C_{17}H_{25}NO_{12}$	Na^+	435.1377	3.53%
A: 5 B: 1	6	$C_{17}H_{25}NO_{12}$	K^+		
A: 5 B: 1	6	$C_{17}H_{25}NO_{12}$	H^+		
A: 6 B: 0	6	$C_{18}H_{26}O_{13}$	Na^+	450.1373	2.49%
A: 6 B: 0	6	$C_{18}H_{26}O_{13}$	K^+		
A: 3 B: 4	7	$C_{17}H_{26}N_4O_{11}$	K^+	462.1598	0.59%
A: 3 B: 4	7	$C_{17}H_{26}N_4O_{11}$	Na^+		
A: 4 B: 3	7	$C_{18}H_{27}N_3O_{12}$	Na^+	477.1595	0.95%
A: 4 B: 3	7	$C_{18}H_{27}N_3O_{12}$	K^+		
A: 5 B: 2	7	$C_{19}H_{28}N_2O_{13}$	Na^+	492.1591	1.21%

Composition					Monoisotopic mass	Quantity
A: 6	B: 1	7	$C_{20}H_{29}NO_{14}$	Na^+	507.1588	3.71%
A: 6	B: 1	7	$C_{20}H_{29}NO_{14}$	K^+		
A: 6	B: 1	7	$C_{20}H_{29}NO_{14}$	H^+		
A: 7	B: 0	7	$C_{21}H_{30}O_{15}$	Na^+	522.1585	2.58%
A: 7	B: 0	7	$C_{21}H_{30}O_{15}$	K^+		
A: 1	B: 8	9	$C_{19}H_{30}N_8O_{11}$	H^+	546.2034	0.53%
A: 5	B: 3	8	$C_{21}H_{31}N_3O_{14}$	K^+	549.1806	1.08%
A: 5	B: 3	8	$C_{21}H_{31}N_3O_{14}$	Na^+		
A: 6	B: 2	8	$C_{22}H_{32}N_2O_{15}$	Na^+	564.1803	2.38%
A: 6	B: 2	8	$C_{22}H_{32}N_2O_{15}$	K^+		
A: 7	B: 1	8	$C_{23}H_{33}NO_{16}$	Na^+	579.1799	3.74%
A: 7	B: 1	8	$C_{23}H_{33}NO_{16}$	K^+		
A: 7	B: 1	8	$C_{23}H_{33}NO_{16}$	H^+		
A: 0	B: 10	10	$C_{20}H_{32}N_{10}O_{11}$	H^+	588.2252	0.36%
A: 8	B: 0	8	$C_{24}H_{34}O_{17}$	Na^+	594.1796	2.32%
A: 8	B: 0	8	$C_{24}H_{34}O_{17}$	K^+		
A: 5	B: 4	9	$C_{23}H_{34}N_4O_{15}$	K^+	606.2021	0.67%
A: 5	B: 4	9	$C_{23}H_{34}N_4O_{15}$	Na^+		
A: 2	B: 8	10	$C_{22}H_{34}N_8O_{13}$	H^+	618.2245	0.59%
A: 6	B: 3	9	$C_{24}H_{35}N_3O_{16}$	Na^+	621.2017	1.15%
A: 6	B: 3	9	$C_{24}H_{35}N_3O_{16}$	K^+		
A: 7	B: 2	9	$C_{25}H_{36}N_2O_{17}$	Na^+	636.2014	2.16%
A: 7	B: 2	9	$C_{25}H_{36}N_2O_{17}$	K^+		
A: 8	B: 1	9	$C_{26}H_{37}NO_{18}$	Na^+	651.2011	2.91%
A: 8	B: 1	9	$C_{26}H_{37}NO_{18}$	K^+		
A: 8	B: 1	9	$C_{26}H_{37}NO_{18}$	H^+		
A: 1	B: 10	11	$C_{23}H_{36}N_{10}O_{13}$	K^+	660.2463	0.99%
A: 1	B: 10	11	$C_{23}H_{36}N_{10}O_{13}$	H^+		
A: 9	B: 0	9	$C_{27}H_{38}O_{19}$	Na^+	666.2007	1.79%
A: 9	B: 0	9	$C_{27}H_{38}O_{19}$	K^+		
A: 2	B: 9	11	$C_{24}H_{37}N_9O_{14}$	H^+	675.2460	1.13%
A: 2	B: 9	11	$C_{24}H_{37}N_9O_{14}$	Na^+		
A: 6	B: 4	10	$C_{26}H_{38}N_4O_{17}$	K^+	678.2232	0.37%
A: 3	B: 8	11	$C_{25}H_{38}N_8O_{15}$	H^+	690.2457	0.46%
A: 7	B: 3	10	$C_{27}H_{39}N_3O_{18}$	Na^+	693.2229	1.14%
A: 7	B: 3	10	$C_{27}H_{39}N_3O_{18}$	K^+		

Composition			Monoisotopic mass	Quantity
A: 8 B: 2<br>A: 8 B: 2	10<br>10	$C_{28}H_{40}N_2O_{19}$ Na^+<br>$C_{28}H_{40}N_2O_{19}$ K^+	708.2225	1.79%
A: 9 B: 1<br>A: 9 B: 1	10<br>10	$C_{29}H_{41}NO_{20}$ Na^+<br>$C_{29}H_{41}NO_{20}$ K^+	723.2222	1.97%
A: 10 B: 0	10	$C_{30}H_{42}O_{21}$ Na^+	738.2219	0.97%
A: 3 B: 9	12	$C_{27}H_{41}N_9O_{16}$ H^+	747.2671	0.45%
A: 7 B: 4	11	$C_{29}H_{42}N_4O_{19}$ K^+	750.2443	0.35%
A: 4 B: 8	12	$C_{28}H_{42}N_8O_{17}$ H^+	762.2668	0.37%
A: 8 B: 3<br>A: 8 B: 3	11<br>11	$C_{30}H_{43}N_3O_{20}$ K^+<br>$C_{30}H_{43}N_3O_{20}$ Na^+	765.2440	0.91%
A: 9 B: 2<br>A: 9 B: 2	11<br>11	$C_{31}H_{44}N_2O_{21}$ Na^+<br>$C_{31}H_{44}N_2O_{21}$ K^+	780.2437	1.26%
A: 10 B: 1<br>A: 10 B: 1	11<br>11	$C_{32}H_{45}NO_{22}$ Na^+<br>$C_{32}H_{45}NO_{22}$ K^+	795.2433	1.30%
A: 3 B: 10	13	$C_{29}H_{44}N_{10}O_{17}$ H^+	804.2886	0.32%
A: 11 B: 0	11	$C_{33}H_{46}O_{23}$ Na^+	810.2430	0.62%
A: 8 B: 4	12	$C_{32}H_{46}N_4O_{21}$ K^+	822.2655	0.31%
A: 9 B: 3<br>A: 9 B: 3	12<br>12	$C_{33}H_{47}N_3O_{22}$ K^+<br>$C_{33}H_{47}N_3O_{22}$ Na^+	837.2651	0.81%
A: 10 B: 2<br>A: 10 B: 2	12<br>12	$C_{34}H_{48}N_2O_{23}$ Na^+<br>$C_{34}H_{48}N_2O_{23}$ K^+	852.2648	1.00%
A: 11 B: 1	12	$C_{35}H_{49}NO_{24}$ Na^+	867.2645	0.63%
A: 12 B: 0	12	$C_{36}H_{50}O_{25}$ Na^+	882.2641	0.41%
A: 10 B: 3<br>A: 10 B: 3	13<br>13	$C_{36}H_{51}N_3O_{24}$ K^+<br>$C_{36}H_{51}N_3O_{24}$ Na^+	909.2862	0.64%
A: 11 B: 2	13	$C_{37}H_{52}N_2O_{25}$ Na^+	924.2859	0.41%
A: 12 B: 1	13	$C_{38}H_{53}NO_{26}$ Na^+	939.2856	0.41%
A: 13 B: 0	13	$C_{39}H_{54}O_{27}$ Na^+	954.2852	0.28%
A: 12 B: 2	14	$C_{40}H_{56}N_2O_{27}$ Na^+	996.3070	0.32%
A: 13 B: 1	14	$C_{41}H_{57}NO_{28}$ Na^+	1011.3067	0.47%

Analytical report

Page under construction...

aG MALDI, 4cyc

Analysis parameters

Ionizations

MF	Monoisotopic mass	m/z mass
Na^{+}	22.98977	22.98922
K^{+}	38.96371	38.96316
H^{+}	1.00783	1.00728

End groups

α	ω	Color
H	OH	

Monomers

	Description	mf	Monoisotopic mass	min	max
A	lactic acid	$C_3H_4O_2$	72.02113	0	100
B	glycine	C_2H_3NO	57.02146	0	100

Experimental spectrum

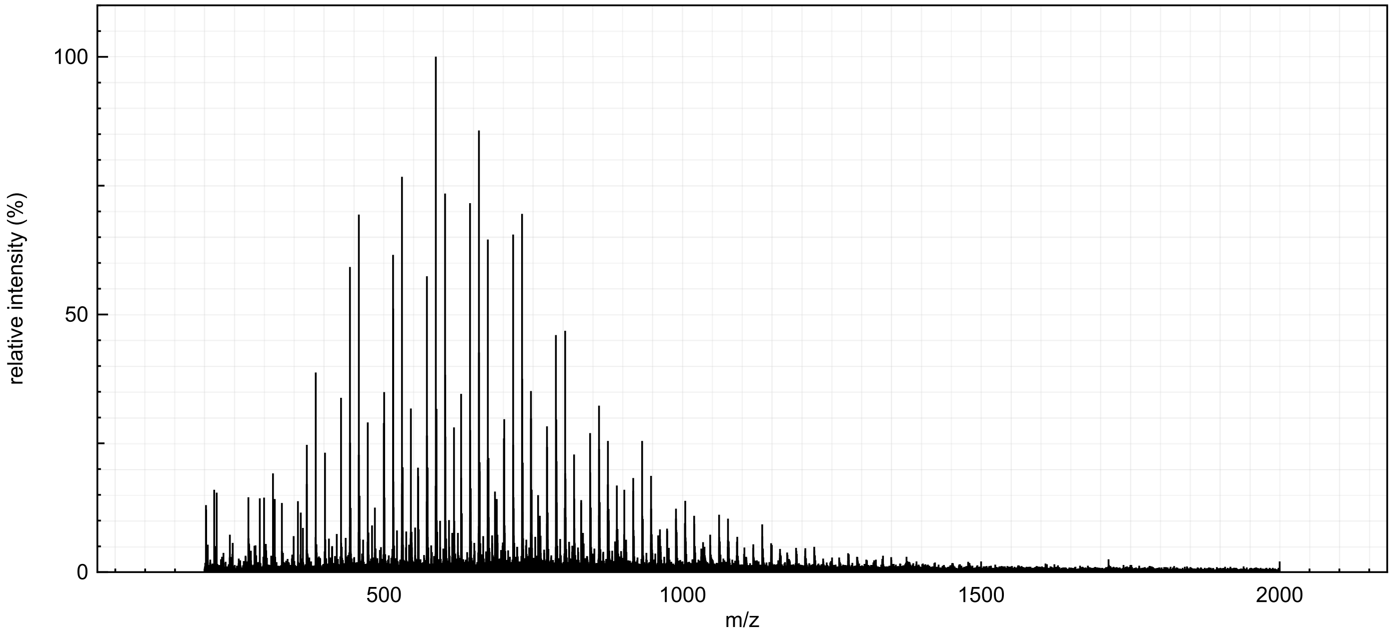

Reconstructed spectrum

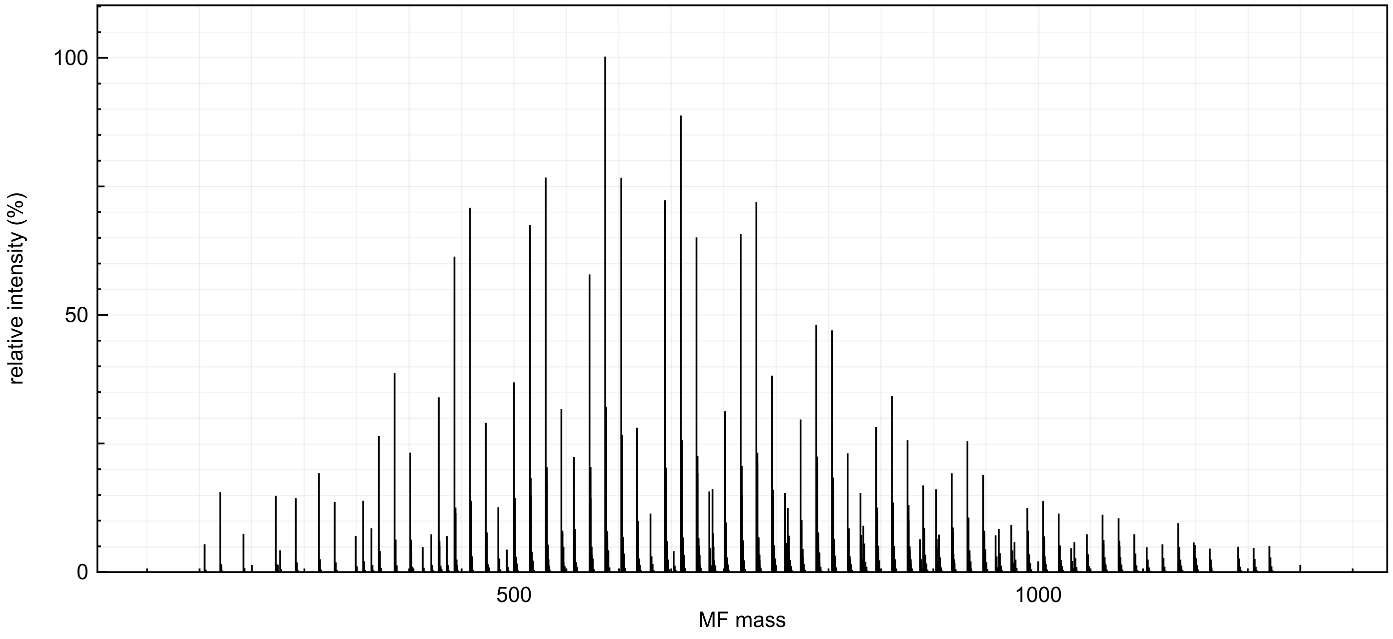

Error (ppm) versus m/z

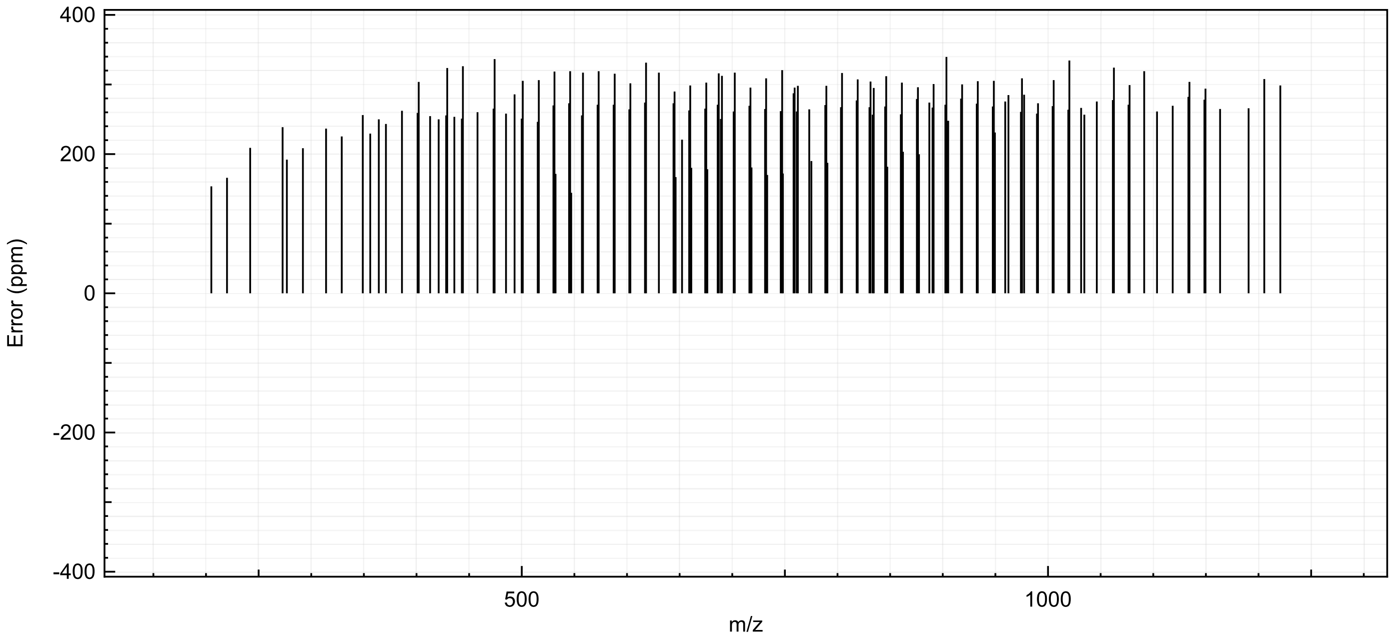

Analysis result

Percent of peaks covered: 72.14%
Number of assigned peaks: 141

Polymers [m/z]

Composition		Nb units	MF	Ioniz.	em [m/z]	Charge	Similarity	Quantity
A: 1	B: 2	3	$C_7H_{12}N_2O_5$	H^+	205.08190	1	42.19%	0.18%
A: 2	B: 1	3	$C_8H_{13}NO_6$	H^+	220.08156	1	64.63%	0.24%
A: 2	B: 1	3	$C_8H_{13}NO_6$	Na^+	242.06351	1	44.71%	0.24%
A: 3	B: 0	3	$C_9H_{14}O_7$	K^+	273.03711	1	55.25%	0.36%
A: 2	B: 2	4	$C_{10}H_{16}N_2O_7$	H^+	277.10303	1	45.66%	0.16%
A: 3	B: 1	4	$C_{11}H_{17}NO_8$	H^+	292.10269	1	63.01%	0.22%
A: 3	B: 1	4	$C_{11}H_{17}NO_8$	Na^+	314.08464	1	41.01%	0.52%
A: 4	B: 0	4	$C_{12}H_{18}O_9$	Na^+	329.08430	1	54.93%	0.29%
A: 2	B: 3	5	$C_{12}H_{19}N_3O_8$	Na^+	356.10644	1	54.38%	0.27%
A: 3	B: 2	5	$C_{13}H_{20}N_2O_9$	H^+	349.12416	1	47.11%	0.20%
A: 3	B: 2	5	$C_{13}H_{20}N_2O_9$	Na^+	371.10610	1	73.80%	0.45%
A: 4	B: 1	5	$C_{14}H_{21}NO_{10}$	H^+	364.12382	1	52.13%	0.25%
A: 4	B: 1	5	$C_{14}H_{21}NO_{10}$	Na^+	386.10577	1	74.56%	0.52%
A: 4	B: 1	5	$C_{14}H_{21}NO_{10}$	K^+	402.07970	1	51.80%	0.24%
A: 5	B: 0	5	$C_{15}H_{22}O_{11}$	Na^+	401.10543	1	64.89%	0.38%
A: 2	B: 4	6	$C_{14}H_{22}N_4O_9$	Na^+	413.12790	1	56.66%	0.24%
A: 2	B: 4	6	$C_{14}H_{22}N_4O_9$	K^+	429.10184	1	57.16%	0.29%
A: 3	B: 3	6	$C_{15}H_{23}N_3O_{10}$	Na^+	428.12756	1	69.34%	0.51%
A: 3	B: 3	6	$C_{15}H_{23}N_3O_{10}$	K^+	444.10150	1	63.36%	0.34%
A: 4	B: 2	6	$C_{16}H_{24}N_2O_{11}$	H^+	421.14529	1	51.29%	0.24%
A: 4	B: 2	6	$C_{16}H_{24}N_2O_{11}$	Na^+	443.12723	1	82.45%	0.82%
A: 5	B: 1	6	$C_{17}H_{25}NO_{12}$	H^+	436.14495	1	49.95%	0.26%
A: 5	B: 1	6	$C_{17}H_{25}NO_{12}$	Na^+	458.12690	1	81.60%	0.90%
A: 5	B: 1	6	$C_{17}H_{25}NO_{12}$	K^+	474.10083	1	59.80%	0.32%
A: 6	B: 0	6	$C_{18}H_{26}O_{13}$	Na^+	473.12656	1	68.75%	0.50%
A: 3	B: 4	7	$C_{17}H_{26}N_4O_{11}$	Na^+	485.14903	1	62.52%	0.37%
A: 3	B: 4	7	$C_{17}H_{26}N_4O_{11}$	K^+	501.12297	1	65.74%	0.37%
A: 4	B: 3	7	$C_{18}H_{27}N_3O_{12}$	Na^+	500.14869	1	79.34%	0.73%
A: 4	B: 3	7	$C_{18}H_{27}N_3O_{12}$	K^+	516.12263	1	71.25%	0.51%

Composition	Nb units	MF	Ioniz.	em [m/z]	Charge	Similarity	Quantity
A: 5 B: 2	7	$C_{19}H_{28}N_2O_{13}$	H^+	493.16642	1	40.99%	0.28%
A: 5 B: 2	7	$C_{19}H_{28}N_2O_{13}$	Na^+	515.14836	1	84.65%	1.13%
A: 5 B: 2	7	$C_{19}H_{28}N_2O_{13}$	K^+	531.12230	1	70.89%	0.52%
A: 6 B: 1	7	$C_{20}H_{29}NO_{14}$	Na^+	530.14803	1	82.53%	1.07%
A: 6 B: 1	7	$C_{20}H_{29}NO_{14}$	K^+	546.12196	1	60.40%	0.37%
A: 3 B: 5	8	$C_{19}H_{29}N_5O_{12}$	K^+	558.14443	1	61.26%	0.34%
A: 7 B: 0	7	$C_{21}H_{30}O_{15}$	Na^+	545.14769	1	72.38%	0.58%
A: 0 B: 9	9	$C_{18}H_{29}N_9O_{10}$	H^+	532.21101	1	50.15%	0.33%
A: 4 B: 4	8	$C_{20}H_{30}N_4O_{13}$	Na^+	557.17016	1	74.36%	0.57%
A: 4 B: 4	8	$C_{20}H_{30}N_4O_{13}$	K^+	573.14409	1	71.73%	0.49%
A: 1 B: 8	9	$C_{19}H_{30}N_8O_{11}$	H^+	547.21068	1	47.09%	0.33%
A: 5 B: 3	8	$C_{21}H_{31}N_3O_{14}$	Na^+	572.16982	1	81.81%	1.00%
A: 5 B: 3	8	$C_{21}H_{31}N_3O_{14}$	K^+	588.14376	1	76.16%	0.66%
A: 6 B: 2	8	$C_{22}H_{32}N_2O_{15}$	Na^+	587.16949	1	82.91%	1.42%
A: 6 B: 2	8	$C_{22}H_{32}N_2O_{15}$	K^+	603.14343	1	74.57%	0.61%
A: 7 B: 1	8	$C_{23}H_{33}NO_{16}$	Na^+	602.16915	1	82.00%	1.24%
A: 7 B: 1	8	$C_{23}H_{33}NO_{16}$	K^+	618.14309	1	64.56%	0.44%
A: 4 B: 5	9	$C_{22}H_{33}N_5O_{14}$	K^+	630.16556	1	66.13%	0.41%
A: 8 B: 0	8	$C_{24}H_{34}O_{17}$	Na^+	617.16882	1	69.48%	0.63%
A: 5 B: 4	9	$C_{23}H_{34}N_4O_{15}$	K^+	645.16522	1	71.57%	0.59%
A: 6 B: 3	9	$C_{24}H_{35}N_3O_{16}$	Na^+	644.19095	1	82.93%	1.18%
A: 6 B: 3	9	$C_{24}H_{35}N_3O_{16}$	K^+	660.16489	1	74.31%	0.69%
A: 7 B: 2	9	$C_{25}H_{36}N_2O_{17}$	Na^+	659.19062	1	85.24%	1.39%
A: 7 B: 2	9	$C_{25}H_{36}N_2O_{17}$	K^+	675.16456	1	75.18%	0.59%
A: 0 B: 11	11	$C_{22}H_{35}N_{11}O_{12}$	H^+	646.25394	1	51.54%	0.41%
A: 4 B: 6	10	$C_{24}H_{36}N_6O_{15}$	K^+	687.18702	1	41.61%	0.56%
A: 8 B: 1	9	$C_{26}H_{37}NO_{18}$	H^+	652.20834	1	47.75%	0.30%
A: 8 B: 1	9	$C_{26}H_{37}NO_{18}$	Na^+	674.19028	1	81.65%	1.10%
A: 8 B: 1	9	$C_{26}H_{37}NO_{18}$	K^+	690.16422	1	63.47%	0.40%
A: 1 B: 10	11	$C_{23}H_{36}N_{10}O_{13}$	H^+	661.25361	1	50.43%	0.43%
A: 5 B: 5	10	$C_{25}H_{37}N_5O_{16}$	Na^+	686.21275	1	46.84%	0.67%
A: 5 B: 5	10	$C_{25}H_{37}N_5O_{16}$	K^+	702.18669	1	68.04%	0.49%
A: 9 B: 0	9	$C_{27}H_{38}O_{19}$	Na^+	689.18995	1	66.13%	0.54%
A: 2 B: 9	11	$C_{24}H_{37}N_9O_{14}$	H^+	676.25327	1	53.93%	0.39%

Composition	Nb units	MF	Ioniz.	em [m/z]	Charge	Similarity	Quantity
A: 6 B: 4	10	$C_{26}H_{38}N_4O_{17}$	Na^+	701.21242	1	77.95%	0.84%
A: 6 B: 4	10	$C_{26}H_{38}N_4O_{17}$	K^+	717.18635	1	75.35%	0.59%
A: 7 B: 3	10	$C_{27}H_{39}N_3O_{18}$	Na^+	716.21208	1	85.02%	1.14%
A: 7 B: 3	10	$C_{27}H_{39}N_3O_{18}$	K^+	732.18602	1	74.83%	0.66%
A: 8 B: 2	10	$C_{28}H_{40}N_2O_{19}$	Na^+	731.21175	1	85.73%	1.26%
A: 8 B: 2	10	$C_{28}H_{40}N_2O_{19}$	K^+	747.18568	1	72.50%	0.52%
A: 1 B: 11	12	$C_{25}H_{39}N_{11}O_{14}$	H^+	718.27507	1	54.39%	0.38%
A: 5 B: 6	11	$C_{27}H_{40}N_6O_{17}$	K^+	759.20815	1	47.81%	0.55%
A: 9 B: 1	10	$C_{29}H_{41}NO_{20}$	Na^+	746.21141	1	80.68%	0.90%
A: 9 B: 1	10	$C_{29}H_{41}NO_{20}$	K^+	762.18535	1	64.39%	0.38%
A: 2 B: 10	12	$C_{26}H_{40}N_{10}O_{15}$	H^+	733.27474	1	49.37%	0.44%
A: 6 B: 5	11	$C_{28}H_{41}N_5O_{18}$	Na^+	758.23388	1	55.96%	0.69%
A: 10 B: 0	10	$C_{30}H_{42}O_{21}$	Na^+	761.21108	1	66.59%	0.49%
A: 3 B: 9	12	$C_{27}H_{41}N_9O_{16}$	H^+	748.27440	1	54.23%	0.37%
A: 7 B: 4	11	$C_{29}H_{42}N_4O_{19}$	Na^+	773.23355	1	80.63%	0.76%
A: 7 B: 4	11	$C_{29}H_{42}N_4O_{19}$	K^+	789.20748	1	79.44%	0.65%
A: 8 B: 3	11	$C_{30}H_{43}N_3O_{20}$	Na^+	788.23321	1	80.40%	1.07%
A: 8 B: 3	11	$C_{30}H_{43}N_3O_{20}$	K^+	804.20715	1	79.56%	0.57%
A: 1 B: 12	13	$C_{27}H_{42}N_{12}O_{15}$	H^+	775.29654	1	52.99%	0.34%
A: 9 B: 2	11	$C_{31}H_{44}N_2O_{21}$	Na^+	803.23288	1	82.59%	0.97%
A: 9 B: 2	11	$C_{31}H_{44}N_2O_{21}$	K^+	819.20681	1	73.39%	0.42%
A: 2 B: 11	13	$C_{28}H_{43}N_{11}O_{16}$	H^+	790.29620	1	56.73%	0.42%
A: 6 B: 6	12	$C_{30}H_{44}N_6O_{19}$	K^+	831.22928	1	54.36%	0.47%
A: 10 B: 1	11	$C_{32}H_{45}NO_{22}$	Na^+	818.23254	1	78.32%	0.64%
A: 10 B: 1	11	$C_{32}H_{45}NO_{22}$	K^+	834.20648	1	65.82%	0.33%
A: 7 B: 5	12	$C_{31}H_{45}N_5O_{20}$	Na^+	830.25501	1	62.17%	0.60%
A: 7 B: 5	12	$C_{31}H_{45}N_5O_{20}$	K^+	846.22895	1	71.39%	0.49%
A: 11 B: 0	11	$C_{33}H_{46}O_{23}$	Na^+	833.23221	1	65.37%	0.40%
A: 8 B: 4	12	$C_{32}H_{46}N_4O_{21}$	Na^+	845.25468	1	77.46%	0.75%
A: 8 B: 4	12	$C_{32}H_{46}N_4O_{21}$	K^+	861.22861	1	75.77%	0.53%
A: 9 B: 3	12	$C_{33}H_{47}N_3O_{22}$	Na^+	860.25434	1	80.92%	0.87%
A: 9 B: 3	12	$C_{33}H_{47}N_3O_{22}$	K^+	876.22828	1	77.22%	0.49%
A: 2 B: 12	14	$C_{30}H_{46}N_{12}O_{17}$	H^+	847.31766	1	53.76%	0.38%
A: 10 B: 2	12	$C_{34}H_{48}N_2O_{23}$	Na^+	875.25401	1	80.68%	0.76%

Composition	Nb units	MF	Ioniz.	em [m/z]	Charge	Similarity	Quantity
A: 10 B: 2	12	$C_{34}H_{48}N_2O_{23}$	K^+	891.22794	1	73.86%	0.42%
A: 3 B: 11	14	$C_{31}H_{47}N_{11}O_{18}$	H^+	862.31733	1	58.06%	0.37%
A: 7 B: 6	13	$C_{33}H_{48}N_6O_{21}$	Na^+	887.27647	1	44.78%	0.61%
A: 7 B: 6	13	$C_{33}H_{48}N_6O_{21}$	K^+	903.25041	1	62.04%	0.46%
A: 11 B: 1	12	$C_{35}H_{49}NO_{24}$	Na^+	890.25367	1	75.05%	0.55%
A: 4 B: 10	14	$C_{32}H_{48}N_{10}O_{19}$	H^+	877.31700	1	58.31%	0.36%
A: 8 B: 5	13	$C_{34}H_{49}N_5O_{22}$	Na^+	902.27614	1	65.11%	0.56%
A: 8 B: 5	13	$C_{34}H_{49}N_5O_{22}$	K^+	918.25008	1	73.21%	0.38%
A: 12 B: 0	12	$C_{36}H_{50}O_{25}$	Na^+	905.25334	1	63.02%	0.38%
A: 9 B: 4	13	$C_{35}H_{50}N_4O_{23}$	Na^+	917.27580	1	81.17%	0.60%
A: 9 B: 4	13	$C_{35}H_{50}N_4O_{23}$	K^+	933.24974	1	78.44%	0.42%
A: 10 B: 3	13	$C_{36}H_{51}N_3O_{24}$	Na^+	932.27547	1	80.58%	0.67%
A: 10 B: 3	13	$C_{36}H_{51}N_3O_{24}$	K^+	948.24941	1	75.98%	0.42%
A: 11 B: 2	13	$C_{37}H_{52}N_2O_{25}$	Na^+	947.27514	1	77.57%	0.62%
A: 8 B: 6	14	$C_{36}H_{52}N_6O_{23}$	Na^+	959.29760	1	53.54%	0.47%
A: 8 B: 6	14	$C_{36}H_{52}N_6O_{23}$	K^+	975.27154	1	62.83%	0.37%
A: 12 B: 1	13	$C_{38}H_{53}NO_{26}$	Na^+	962.27480	1	72.33%	0.40%
A: 5 B: 10	15	$C_{35}H_{52}N_{10}O_{21}$	H^+	949.33813	1	61.15%	0.33%
A: 9 B: 5	14	$C_{37}H_{53}N_5O_{24}$	Na^+	974.29727	1	66.80%	0.45%
A: 9 B: 5	14	$C_{37}H_{53}N_5O_{24}$	K^+	990.27121	1	73.99%	0.38%
A: 13 B: 0	13	$C_{39}H_{54}O_{27}$	Na^+	977.27447	1	62.09%	0.31%
A: 10 B: 4	14	$C_{38}H_{54}N_4O_{25}$	Na^+	989.29693	1	79.09%	0.53%
A: 10 B: 4	14	$C_{38}H_{54}N_4O_{25}$	K^+	1005.27087	1	75.84%	0.37%
A: 11 B: 3	14	$C_{39}H_{55}N_3O_{26}$	Na^+	1004.29660	1	77.46%	0.53%
A: 11 B: 3	14	$C_{39}H_{55}N_3O_{26}$	K^+	1020.27054	1	77.59%	0.33%
A: 12 B: 2	14	$C_{40}H_{56}N_2O_{27}$	Na^+	1019.29627	1	78.68%	0.45%
A: 9 B: 6	15	$C_{39}H_{56}N_6O_{25}$	Na^+	1031.31873	1	51.09%	0.38%
A: 13 B: 1	14	$C_{41}H_{57}NO_{28}$	Na^+	1034.29593	1	57.43%	0.44%
A: 10 B: 5	15	$C_{40}H_{57}N_5O_{26}$	Na^+	1046.31840	1	68.10%	0.39%
A: 10 B: 5	15	$C_{40}H_{57}N_5O_{26}$	K^+	1062.29233	1	79.95%	0.35%
A: 11 B: 4	15	$C_{41}H_{58}N_4O_{27}$	Na^+	1061.31806	1	77.34%	0.47%
A: 11 B: 4	15	$C_{41}H_{58}N_4O_{27}$	K^+	1077.29200	1	80.38%	0.33%
A: 12 B: 3	15	$C_{42}H_{59}N_3O_{28}$	Na^+	1076.31773	1	76.11%	0.44%
A: 13 B: 2	15	$C_{43}H_{60}N_2O_{29}$	Na^+	1091.31739	1	71.79%	0.35%

Composition	Nb units	MF	Ioniz.	em [m/z]	Charge	Similarity	Quantity
A: 10 B: 6	16	$C_{42}H_{60}N_6O_{27}$	Na^+	1103.33986	1	63.37%	0.32%
A: 11 B: 5	16	$C_{43}H_{61}N_5O_{28}$	Na^+	1118.33953	1	70.69%	0.33%
A: 11 B: 5	16	$C_{43}H_{61}N_5O_{28}$	K^+	1134.31346	1	77.50%	0.30%
A: 12 B: 4	16	$C_{44}H_{62}N_4O_{29}$	Na^+	1133.33919	1	76.77%	0.39%
A: 12 B: 4	16	$C_{44}H_{62}N_4O_{29}$	K^+	1149.31313	1	76.97%	0.26%
A: 13 B: 3	16	$C_{45}H_{63}N_3O_{30}$	Na^+	1148.33886	1	76.66%	0.32%
A: 14 B: 2	16	$C_{46}H_{64}N_2O_{31}$	Na^+	1163.33852	1	71.54%	0.30%
A: 12 B: 5	17	$C_{46}H_{65}N_5O_{30}$	Na^+	1190.36066	1	76.19%	0.28%
A: 13 B: 4	17	$C_{47}H_{66}N_4O_{31}$	Na^+	1205.36032	1	78.36%	0.29%
A: 14 B: 3	17	$C_{48}H_{67}N_3O_{32}$	Na^+	1220.35999	1	76.63%	0.28%

Polymers grouped by monoisotopic mass

Composition				Monoisotopic mass	Quantity
A: 1 B: 2	3	$C_7H_{12}N_2O_5$	H^+	204.0746	0.18%
A: 2 B: 1<br>A: 2 B: 1	3<br>3	$C_8H_{13}NO_6$<br>$C_8H_{13}NO_6$	H^+<br>Na^+	219.0743	0.49%
A: 3 B: 0	3	$C_9H_{14}O_7$	K^+	234.0740	0.36%
A: 2 B: 2	4	$C_{10}H_{16}N_2O_7$	H^+	276.0958	0.16%
A: 3 B: 1<br>A: 3 B: 1	4<br>4	$C_{11}H_{17}NO_8$<br>$C_{11}H_{17}NO_8$	H^+<br>Na^+	291.0954	0.75%
A: 4 B: 0	4	$C_{12}H_{18}O_9$	Na^+	306.0951	0.29%
A: 2 B: 3	5	$C_{12}H_{19}N_3O_8$	Na^+	333.1172	0.27%
A: 3 B: 2<br>A: 3 B: 2	5<br>5	$C_{13}H_{20}N_2O_9$<br>$C_{13}H_{20}N_2O_9$	Na^+<br>H^+	348.1169	0.65%
A: 4 B: 1<br>A: 4 B: 1<br>A: 4 B: 1	5<br>5<br>5	$C_{14}H_{21}NO_{10}$<br>$C_{14}H_{21}NO_{10}$<br>$C_{14}H_{21}NO_{10}$	Na^+<br>H^+<br>K^+	363.1165	1.00%
A: 5 B: 0	5	$C_{15}H_{22}O_{11}$	Na^+	378.1162	0.38%
A: 2 B: 4<br>A: 2 B: 4	6<br>6	$C_{14}H_{22}N_4O_9$<br>$C_{14}H_{22}N_4O_9$	K^+<br>Na^+	390.1387	0.52%
A: 3 B: 3<br>A: 3 B: 3	6<br>6	$C_{15}H_{23}N_3O_{10}$<br>$C_{15}H_{23}N_3O_{10}$	Na^+<br>K^+	405.1383	0.85%
A: 4 B: 2<br>A: 4 B: 2	6<br>6	$C_{16}H_{24}N_2O_{11}$<br>$C_{16}H_{24}N_2O_{11}$	Na^+<br>H^+	420.1380	1.06%

		Composition			Monoisotopic mass	Quantity
A: 5	B: 1	6	$C_{17}H_{25}NO_{12}$	Na^+	435.1377	1.48%
A: 5	B: 1	6	$C_{17}H_{25}NO_{12}$	K^+		
A: 5	B: 1	6	$C_{17}H_{25}NO_{12}$	H^+		
A: 6	B: 0	6	$C_{18}H_{26}O_{13}$	Na^+	450.1373	0.50%
A: 3	B: 4	7	$C_{17}H_{26}N_4O_{11}$	K^+	462.1598	0.74%
A: 3	B: 4	7	$C_{17}H_{26}N_4O_{11}$	Na^+		
A: 4	B: 3	7	$C_{18}H_{27}N_3O_{12}$	Na^+	477.1595	1.23%
A: 4	B: 3	7	$C_{18}H_{27}N_3O_{12}$	K^+		
A: 5	B: 2	7	$C_{19}H_{28}N_2O_{13}$	Na^+	492.1591	1.93%
A: 5	B: 2	7	$C_{19}H_{28}N_2O_{13}$	K^+		
A: 5	B: 2	7	$C_{19}H_{28}N_2O_{13}$	H^+		
A: 6	B: 1	7	$C_{20}H_{29}NO_{14}$	Na^+	507.1588	1.45%
A: 6	B: 1	7	$C_{20}H_{29}NO_{14}$	K^+		
A: 3	B: 5	8	$C_{19}H_{29}N_5O_{12}$	K^+	519.1813	0.34%
A: 7	B: 0	7	$C_{21}H_{30}O_{15}$	Na^+	522.1585	0.58%
A: 0	B: 9	9	$C_{18}H_{29}N_9O_{10}$	H^+	531.2037	0.33%
A: 4	B: 4	8	$C_{20}H_{30}N_4O_{13}$	Na^+	534.1809	1.05%
A: 4	B: 4	8	$C_{20}H_{30}N_4O_{13}$	K^+		
A: 1	B: 8	9	$C_{19}H_{30}N_8O_{11}$	H^+	546.2034	0.33%
A: 5	B: 3	8	$C_{21}H_{31}N_3O_{14}$	Na^+	549.1806	1.66%
A: 5	B: 3	8	$C_{21}H_{31}N_3O_{14}$	K^+		
A: 6	B: 2	8	$C_{22}H_{32}N_2O_{15}$	Na^+	564.1803	2.03%
A: 6	B: 2	8	$C_{22}H_{32}N_2O_{15}$	K^+		
A: 7	B: 1	8	$C_{23}H_{33}NO_{16}$	Na^+	579.1799	1.67%
A: 7	B: 1	8	$C_{23}H_{33}NO_{16}$	K^+		
A: 4	B: 5	9	$C_{22}H_{33}N_5O_{14}$	K^+	591.2024	0.41%
A: 8	B: 0	8	$C_{24}H_{34}O_{17}$	Na^+	594.1796	0.63%
A: 5	B: 4	9	$C_{23}H_{34}N_4O_{15}$	K^+	606.2021	0.59%
A: 6	B: 3	9	$C_{24}H_{35}N_3O_{16}$	Na^+	621.2017	1.87%
A: 6	B: 3	9	$C_{24}H_{35}N_3O_{16}$	K^+		
A: 7	B: 2	9	$C_{25}H_{36}N_2O_{17}$	Na^+	636.2014	1.97%
A: 7	B: 2	9	$C_{25}H_{36}N_2O_{17}$	K^+		
A: 0	B: 11	11	$C_{22}H_{35}N_{11}O_{12}$	H^+	645.2467	0.41%
A: 4	B: 6	10	$C_{24}H_{36}N_6O_{15}$	K^+	648.2239	0.56%

Composition				Monoisotopic mass	Quantity
A: 8 B: 1	9	$C_{26}H_{37}NO_{18}$	Na^+	651.2011	1.80%
A: 8 B: 1	9	$C_{26}H_{37}NO_{18}$	K^+		
A: 8 B: 1	9	$C_{26}H_{37}NO_{18}$	H^+		
A: 1 B: 10	11	$C_{23}H_{36}N_{10}O_{13}$	H^+	660.2463	0.43%
A: 5 B: 5	10	$C_{25}H_{37}N_5O_{16}$	K^+	663.2235	1.16%
A: 5 B: 5	10	$C_{25}H_{37}N_5O_{16}$	Na^+		
A: 9 B: 0	9	$C_{27}H_{38}O_{19}$	Na^+	666.2007	0.54%
A: 2 B: 9	11	$C_{24}H_{37}N_9O_{14}$	H^+	675.2460	0.39%
A: 6 B: 4	10	$C_{26}H_{38}N_4O_{17}$	Na^+	678.2232	1.42%
A: 6 B: 4	10	$C_{26}H_{38}N_4O_{17}$	K^+		
A: 7 B: 3	10	$C_{27}H_{39}N_3O_{18}$	Na^+	693.2229	1.80%
A: 7 B: 3	10	$C_{27}H_{39}N_3O_{18}$	K^+		
A: 8 B: 2	10	$C_{28}H_{40}N_2O_{19}$	Na^+	708.2225	1.77%
A: 8 B: 2	10	$C_{28}H_{40}N_2O_{19}$	K^+		
A: 1 B: 11	12	$C_{25}H_{39}N_{11}O_{14}$	H^+	717.2678	0.38%
A: 5 B: 6	11	$C_{27}H_{40}N_6O_{17}$	K^+	720.2450	0.55%
A: 9 B: 1	10	$C_{29}H_{41}NO_{20}$	Na^+	723.2222	1.29%
A: 9 B: 1	10	$C_{29}H_{41}NO_{20}$	K^+		
A: 2 B: 10	12	$C_{26}H_{40}N_{10}O_{15}$	H^+	732.2675	0.44%
A: 6 B: 5	11	$C_{28}H_{41}N_5O_{18}$	Na^+	735.2447	0.69%
A: 10 B: 0	10	$C_{30}H_{42}O_{21}$	Na^+	738.2219	0.49%
A: 3 B: 9	12	$C_{27}H_{41}N_9O_{16}$	H^+	747.2671	0.37%
A: 7 B: 4	11	$C_{29}H_{42}N_4O_{19}$	Na^+	750.2443	1.42%
A: 7 B: 4	11	$C_{29}H_{42}N_4O_{19}$	K^+		
A: 8 B: 3	11	$C_{30}H_{43}N_3O_{20}$	Na^+	765.2440	1.63%
A: 8 B: 3	11	$C_{30}H_{43}N_3O_{20}$	K^+		
A: 1 B: 12	13	$C_{27}H_{42}N_{12}O_{15}$	H^+	774.2893	0.34%
A: 9 B: 2	11	$C_{31}H_{44}N_2O_{21}$	Na^+	780.2437	1.39%
A: 9 B: 2	11	$C_{31}H_{44}N_2O_{21}$	K^+		
A: 2 B: 11	13	$C_{28}H_{43}N_{11}O_{16}$	H^+	789.2889	0.42%
A: 6 B: 6	12	$C_{30}H_{44}N_6O_{19}$	K^+	792.2661	0.47%
A: 10 B: 1	11	$C_{32}H_{45}NO_{22}$	Na^+	795.2433	0.98%
A: 10 B: 1	11	$C_{32}H_{45}NO_{22}$	K^+		
A: 7 B: 5	12	$C_{31}H_{45}N_5O_{20}$	K^+	807.2658	1.09%
A: 7 B: 5	12	$C_{31}H_{45}N_5O_{20}$	Na^+		

Composition			Monoisotopic mass	Quantity
A: 11 B: 0	11	$C_{33}H_{46}O_{23}$ Na^+	810.2430	0.40%
A: 8 B: 4<br>A: 8 B: 4	12<br>12	$C_{32}H_{46}N_4O_{21}$ Na^+<br>$C_{32}H_{46}N_4O_{21}$ K^+	822.2655	1.28%
A: 9 B: 3<br>A: 9 B: 3	12<br>12	$C_{33}H_{47}N_3O_{22}$ Na^+<br>$C_{33}H_{47}N_3O_{22}$ K^+	837.2651	1.36%
A: 2 B: 12	14	$C_{30}H_{46}N_{12}O_{17}$ H^+	846.3104	0.38%
A: 10 B: 2<br>A: 10 B: 2	12<br>12	$C_{34}H_{48}N_2O_{23}$ Na^+<br>$C_{34}H_{48}N_2O_{23}$ K^+	852.2648	1.18%
A: 3 B: 11	14	$C_{31}H_{47}N_{11}O_{18}$ H^+	861.3101	0.37%
A: 7 B: 6<br>A: 7 B: 6	13<br>13	$C_{33}H_{48}N_6O_{21}$ K^+<br>$C_{33}H_{48}N_6O_{21}$ Na^+	864.2873	1.07%
A: 11 B: 1	12	$C_{35}H_{49}NO_{24}$ Na^+	867.2645	0.55%
A: 4 B: 10	14	$C_{32}H_{48}N_{10}O_{19}$ H^+	876.3097	0.36%
A: 8 B: 5<br>A: 8 B: 5	13<br>13	$C_{34}H_{49}N_5O_{22}$ K^+<br>$C_{34}H_{49}N_5O_{22}$ Na^+	879.2869	0.95%
A: 12 B: 0	12	$C_{36}H_{50}O_{25}$ Na^+	882.2641	0.38%
A: 9 B: 4<br>A: 9 B: 4	13<br>13	$C_{35}H_{50}N_4O_{23}$ Na^+<br>$C_{35}H_{50}N_4O_{23}$ K^+	894.2866	1.02%
A: 10 B: 3<br>A: 10 B: 3	13<br>13	$C_{36}H_{51}N_3O_{24}$ Na^+<br>$C_{36}H_{51}N_3O_{24}$ K^+	909.2862	1.10%
A: 11 B: 2	13	$C_{37}H_{52}N_2O_{25}$ Na^+	924.2859	0.62%
A: 8 B: 6<br>A: 8 B: 6	14<br>14	$C_{36}H_{52}N_6O_{23}$ K^+<br>$C_{36}H_{52}N_6O_{23}$ Na^+	936.3084	0.84%
A: 12 B: 1	13	$C_{38}H_{53}NO_{26}$ Na^+	939.2856	0.40%
A: 5 B: 10	15	$C_{35}H_{52}N_{10}O_{21}$ H^+	948.3308	0.33%
A: 9 B: 5<br>A: 9 B: 5	14<br>14	$C_{37}H_{53}N_5O_{24}$ K^+<br>$C_{37}H_{53}N_5O_{24}$ Na^+	951.3080	0.83%
A: 13 B: 0	13	$C_{39}H_{54}O_{27}$ Na^+	954.2852	0.31%
A: 10 B: 4<br>A: 10 B: 4	14<br>14	$C_{38}H_{54}N_4O_{25}$ Na^+<br>$C_{38}H_{54}N_4O_{25}$ K^+	966.3077	0.89%
A: 11 B: 3<br>A: 11 B: 3	14<br>14	$C_{39}H_{55}N_3O_{26}$ K^+<br>$C_{39}H_{55}N_3O_{26}$ Na^+	981.3074	0.86%
A: 12 B: 2	14	$C_{40}H_{56}N_2O_{27}$ Na^+	996.3070	0.45%
A: 9 B: 6	15	$C_{39}H_{56}N_6O_{25}$ Na^+	1008.3295	0.38%
A: 13 B: 1	14	$C_{41}H_{57}NO_{28}$ Na^+	1011.3067	0.44%

		Composition	Monoisotopic mass	Quantity
A: 10 B: 5	15	$C_{40}H_{57}N_5O_{26}\,K^+$	1023.3292	0.74%
A: 10 B: 5	15	$C_{40}H_{57}N_5O_{26}\,Na^+$		
A: 11 B: 4	15	$C_{41}H_{58}N_4O_{27}\,K^+$	1038.3288	0.80%
A: 11 B: 4	15	$C_{41}H_{58}N_4O_{27}\,Na^+$		
A: 12 B: 3	15	$C_{42}H_{59}N_3O_{28}\,Na^+$	1053.3285	0.44%
A: 13 B: 2	15	$C_{43}H_{60}N_2O_{29}\,Na^+$	1068.3282	0.35%
A: 10 B: 6	16	$C_{42}H_{60}N_6O_{27}\,Na^+$	1080.3506	0.32%
A: 11 B: 5	16	$C_{43}H_{61}N_5O_{28}\,K^+$	1095.3503	0.63%
A: 11 B: 5	16	$C_{43}H_{61}N_5O_{28}\,Na^+$		
A: 12 B: 4	16	$C_{44}H_{62}N_4O_{29}\,K^+$	1110.3500	0.65%
A: 12 B: 4	16	$C_{44}H_{62}N_4O_{29}\,Na^+$		
A: 13 B: 3	16	$C_{45}H_{63}N_3O_{30}\,Na^+$	1125.3496	0.32%
A: 14 B: 2	16	$C_{46}H_{64}N_2O_{31}\,Na^+$	1140.3493	0.30%
A: 12 B: 5	17	$C_{46}H_{65}N_5O_{30}\,Na^+$	1167.3714	0.28%
A: 13 B: 4	17	$C_{47}H_{66}N_4O_{31}\,Na^+$	1182.3711	0.29%
A: 14 B: 3	17	$C_{48}H_{67}N_3O_{32}\,Na^+$	1197.3708	0.28%

Analytical report

Page under construction...

aG MALDI, 8cyc

Analysis parameters

Ionizations

MF	Monoisotopic mass	m/z mass
Na^+	22.98977	22.98922
K^+	38.96371	38.96316
H^+	1.00783	1.00728

End groups

α	ω	Color
H	OH	

Monomers

	Description	mf	Monoisotopic mass	min	max
A	lactic acid	$C_3H_4O_2$	72.02113	0	100
B	glycine	C_2H_3NO	57.02146	0	100

Experimental spectrum

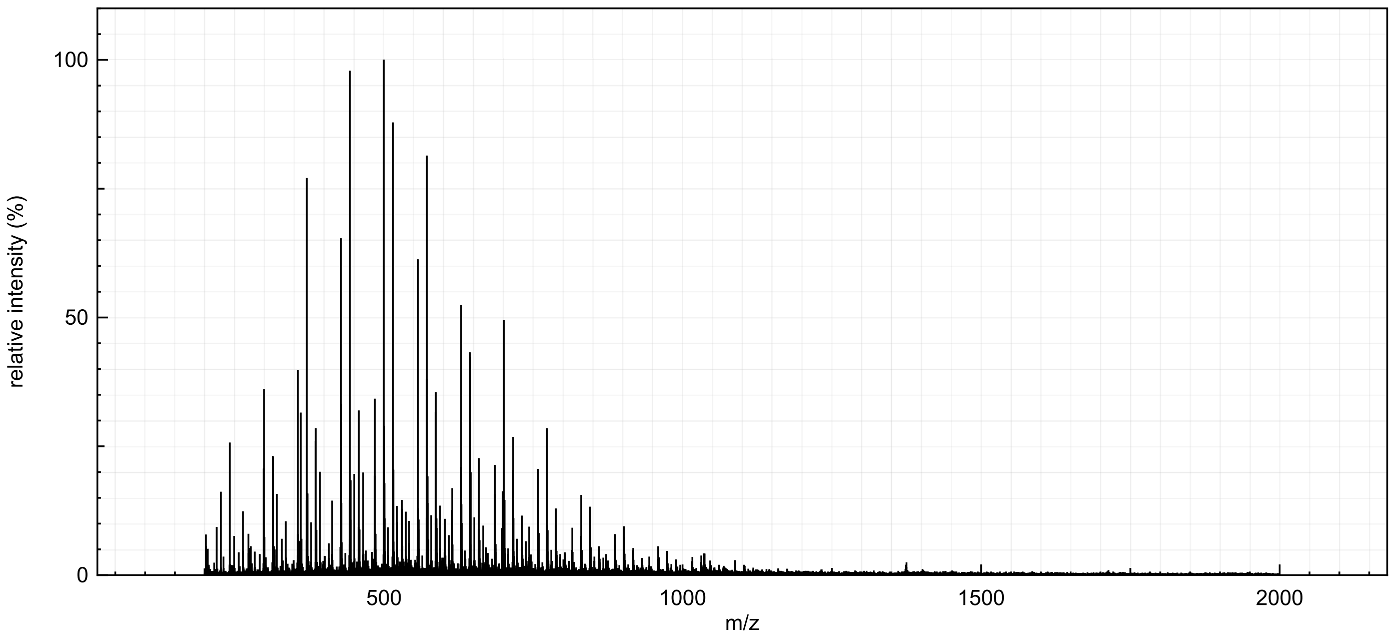

Reconstructed spectrum

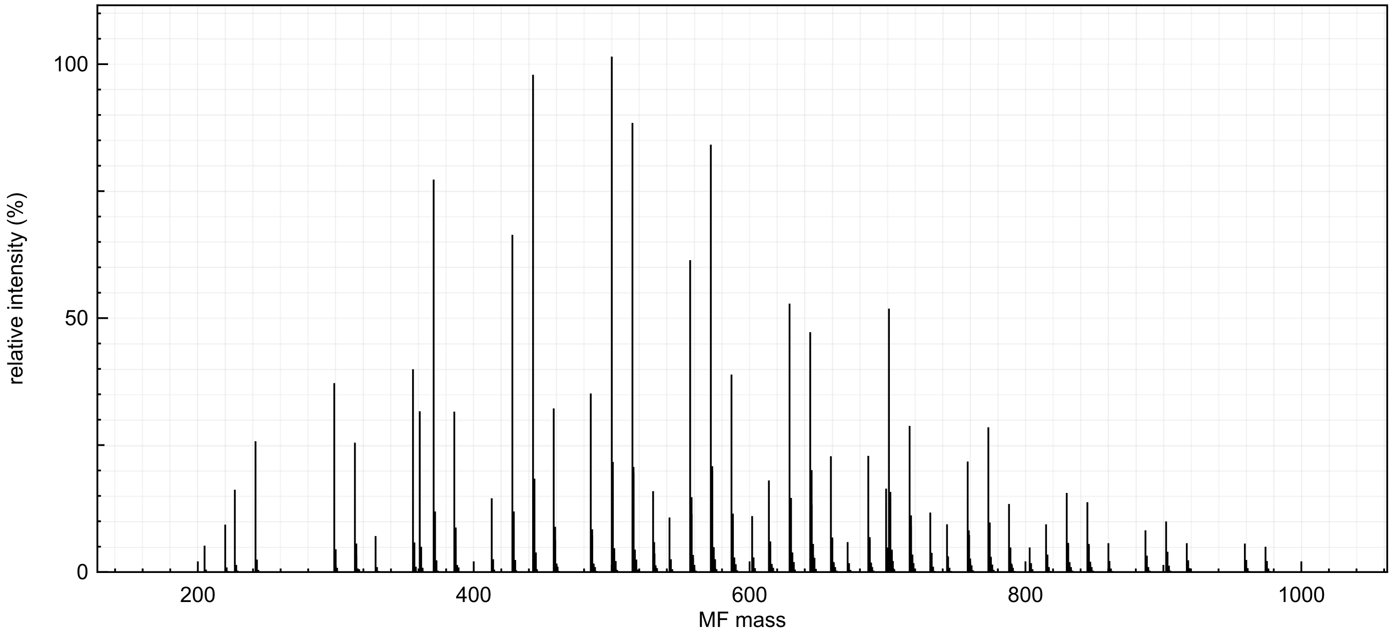

Error (ppm) versus m/z

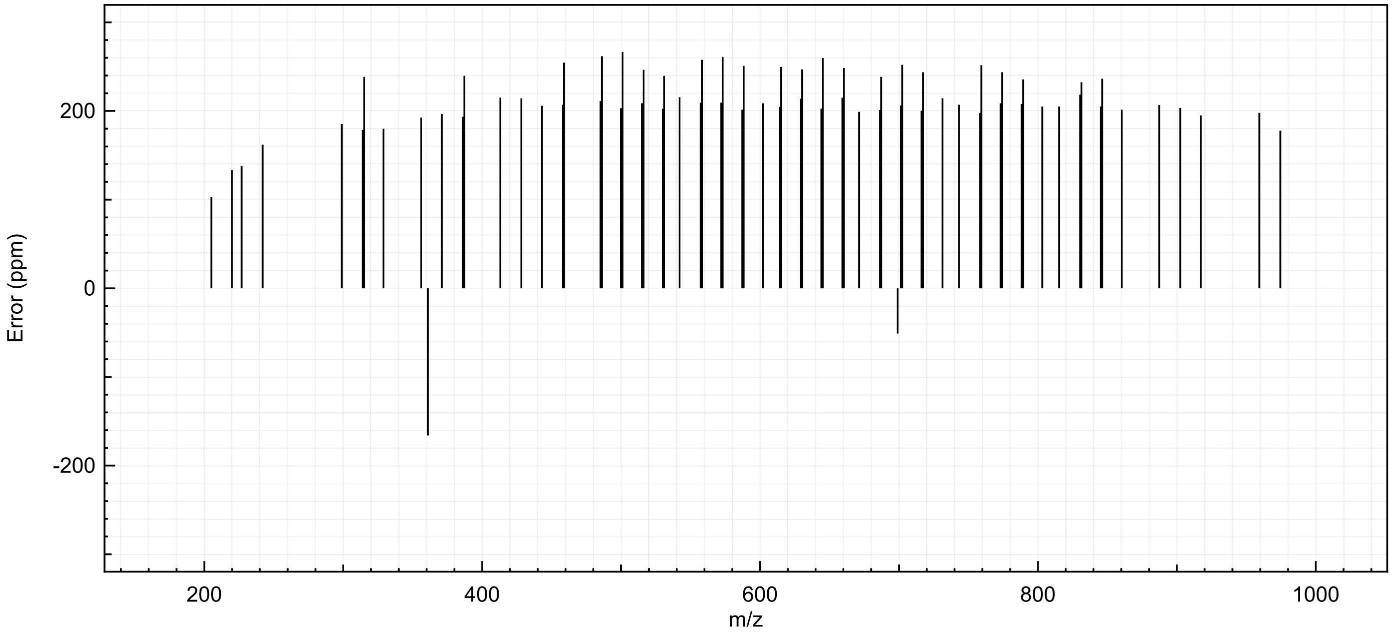

Analysis result

Percent of peaks covered: 50.58%
Number of assigned peaks: 70

Polymers [m/z]

Composition	Nb units	MF	Ioniz.	em [m/z]	Charge	Similarity	Quantity
A: 1 B: 2	3	$C_7H_{12}N_2O_5$	Na^+	227.06384	1	65.42%	0.32%
A: 1 B: 2	3	$C_7H_{12}N_2O_5$	H^+	205.08190	1	41.76%	0.21%
A: 2 B: 1	3	$C_8H_{13}NO_6$	Na^+	242.06351	1	73.25%	0.44%
A: 2 B: 1	3	$C_8H_{13}NO_6$	H^+	220.08156	1	63.82%	0.22%
A: 2 B: 2	4	$C_{10}H_{16}N_2O_7$	Na^+	299.08497	1	77.08%	0.84%
A: 2 B: 2	4	$C_{10}H_{16}N_2O_7$	K^+	315.05891	1	40.43%	0.43%
A: 3 B: 1	4	$C_{11}H_{17}NO_8$	Na^+	314.08464	1	65.25%	0.83%
A: 4 B: 0	4	$C_{12}H_{18}O_9$	Na^+	329.08430	1	48.05%	0.26%
A: 2 B: 3	5	$C_{12}H_{19}N_3O_8$	Na^+	356.10644	1	72.03%	0.81%
A: 3 B: 2	5	$C_{13}H_{20}N_2O_9$	Na^+	371.10610	1	82.94%	1.35%
A: 3 B: 2	5	$C_{13}H_{20}N_2O_9$	K^+	387.08004	1	58.09%	0.37%
A: 0 B: 6	6	$C_{12}H_{20}N_6O_7$	H^+	361.14662	1	71.07%	0.76%
A: 4 B: 1	5	$C_{14}H_{21}NO_{10}$	Na^+	386.10577	1	78.19%	0.85%
A: 2 B: 4	6	$C_{14}H_{22}N_4O_9$	Na^+	413.12790	1	70.74%	0.46%
A: 3 B: 3	6	$C_{15}H_{23}N_3O_{10}$	Na^+	428.12756	1	86.12%	1.44%
A: 4 B: 2	6	$C_{16}H_{24}N_2O_{11}$	Na^+	443.12723	1	86.23%	1.71%
A: 4 B: 2	6	$C_{16}H_{24}N_2O_{11}$	K^+	459.10117	1	58.08%	0.42%
A: 5 B: 1	6	$C_{17}H_{25}NO_{12}$	Na^+	458.12690	1	72.51%	0.78%
A: 2 B: 5	7	$C_{16}H_{25}N_5O_{10}$	K^+	486.12330	1	62.95%	0.43%
A: 3 B: 4	7	$C_{17}H_{26}N_4O_{11}$	Na^+	485.14903	1	82.04%	0.89%
A: 3 B: 4	7	$C_{17}H_{26}N_4O_{11}$	K^+	501.12297	1	70.09%	0.77%
A: 4 B: 3	7	$C_{18}H_{27}N_3O_{12}$	Na^+	500.14869	1	86.87%	1.91%
A: 4 B: 3	7	$C_{18}H_{27}N_3O_{12}$	K^+	516.12263	1	68.91%	0.73%
A: 5 B: 2	7	$C_{19}H_{28}N_2O_{13}$	Na^+	515.14836	1	85.02%	1.63%
A: 5 B: 2	7	$C_{19}H_{28}N_2O_{13}$	K^+	531.12230	1	57.22%	0.41%
A: 6 B: 1	7	$C_{20}H_{29}NO_{14}$	Na^+	530.14803	1	69.64%	0.62%
A: 3 B: 5	8	$C_{19}H_{29}N_5O_{12}$	Na^+	542.17049	1	65.35%	0.51%
A: 3 B: 5	8	$C_{19}H_{29}N_5O_{12}$	K^+	558.14443	1	71.40%	0.54%
A: 4 B: 4	8	$C_{20}H_{30}N_4O_{13}$	Na^+	557.17016	1	86.16%	1.23%

Composition	Nb units	MF	Ioniz.	em [m/z]	Charge	Similarity	Quantity
A: 4 B: 4	8	$C_{20}H_{30}N_4O_{13}$	K^+	573.14409	1	76.05%	0.82%
A: 5 B: 3	8	$C_{21}H_{31}N_3O_{14}$	Na^+	572.16982	1	86.06%	1.86%
A: 5 B: 3	8	$C_{21}H_{31}N_3O_{14}$	K^+	588.14376	1	71.20%	0.61%
A: 6 B: 2	8	$C_{22}H_{32}N_2O_{15}$	Na^+	587.16949	1	79.91%	1.23%
A: 3 B: 6	9	$C_{21}H_{32}N_6O_{13}$	K^+	615.16589	1	57.20%	0.44%
A: 7 B: 1	8	$C_{23}H_{33}NO_{16}$	Na^+	602.16915	1	63.35%	0.50%
A: 4 B: 5	9	$C_{22}H_{33}N_5O_{14}$	Na^+	614.19162	1	72.26%	0.69%
A: 4 B: 5	9	$C_{22}H_{33}N_5O_{14}$	K^+	630.16556	1	74.47%	0.68%
A: 5 B: 4	9	$C_{23}H_{34}N_4O_{15}$	Na^+	629.19129	1	83.47%	1.36%
A: 5 B: 4	9	$C_{23}H_{34}N_4O_{15}$	K^+	645.16522	1	74.21%	0.71%
A: 6 B: 3	9	$C_{24}H_{35}N_3O_{16}$	Na^+	644.19095	1	84.57%	1.46%
A: 6 B: 3	9	$C_{24}H_{35}N_3O_{16}$	K^+	660.16489	1	65.26%	0.46%
A: 7 B: 2	9	$C_{25}H_{36}N_2O_{17}$	Na^+	659.19062	1	74.68%	0.75%
A: 4 B: 6	10	$C_{24}H_{36}N_6O_{15}$	Na^+	671.21309	1	53.11%	0.50%
A: 4 B: 6	10	$C_{24}H_{36}N_6O_{15}$	K^+	687.18702	1	64.47%	0.43%
A: 1 B: 10	11	$C_{23}H_{36}N_{10}O_{13}$	K^+	699.20949	1	40.21%	1.62%
A: 5 B: 5	10	$C_{25}H_{37}N_5O_{16}$	Na^+	686.21275	1	80.31%	0.77%
A: 5 B: 5	10	$C_{25}H_{37}N_5O_{16}$	K^+	702.18669	1	75.96%	0.72%
A: 6 B: 4	10	$C_{26}H_{38}N_4O_{17}$	Na^+	701.21242	1	82.38%	1.35%
A: 6 B: 4	10	$C_{26}H_{38}N_4O_{17}$	K^+	717.18635	1	72.70%	0.55%
A: 7 B: 3	10	$C_{27}H_{39}N_3O_{18}$	Na^+	716.21208	1	80.70%	0.95%
A: 8 B: 2	10	$C_{28}H_{40}N_2O_{19}$	Na^+	731.21175	1	67.91%	0.53%
A: 5 B: 6	11	$C_{27}H_{40}N_6O_{17}$	Na^+	743.23421	1	61.06%	0.53%
A: 5 B: 6	11	$C_{27}H_{40}N_6O_{17}$	K^+	759.20815	1	69.36%	0.48%
A: 6 B: 5	11	$C_{28}H_{41}N_5O_{18}$	Na^+	758.23388	1	77.87%	0.77%
A: 6 B: 5	11	$C_{28}H_{41}N_5O_{18}$	K^+	774.20782	1	73.55%	0.48%
A: 7 B: 4	11	$C_{29}H_{42}N_4O_{19}$	Na^+	773.23355	1	81.33%	0.87%
A: 7 B: 4	11	$C_{29}H_{42}N_4O_{19}$	K^+	789.20748	1	71.72%	0.40%
A: 8 B: 3	11	$C_{30}H_{43}N_3O_{20}$	Na^+	788.23321	1	74.74%	0.61%
A: 9 B: 2	11	$C_{31}H_{44}N_2O_{21}$	Na^+	803.23288	1	63.98%	0.38%
A: 6 B: 6	12	$C_{30}H_{44}N_6O_{19}$	Na^+	815.25534	1	69.30%	0.46%
A: 6 B: 6	12	$C_{30}H_{44}N_6O_{19}$	K^+	831.22928	1	70.71%	0.40%
A: 7 B: 5	12	$C_{31}H_{45}N_5O_{20}$	Na^+	830.25501	1	77.88%	0.64%
A: 7 B: 5	12	$C_{31}H_{45}N_5O_{20}$	K^+	846.22895	1	71.50%	0.38%

Composition	Nb units	MF	Ioniz.	em [m/z]	Charge	Similarity	Quantity
A: 8 B: 4	12	$C_{32}H_{46}N_4O_{21}$	Na^+	845.25468	1	77.17%	0.60%
A: 9 B: 3	12	$C_{33}H_{47}N_3O_{22}$	Na^+	860.25434	1	70.59%	0.38%
A: 7 B: 6	13	$C_{33}H_{48}N_6O_{21}$	Na^+	887.27647	1	69.01%	0.43%
A: 8 B: 5	13	$C_{34}H_{49}N_5O_{22}$	Na^+	902.27614	1	75.82%	0.50%
A: 9 B: 4	13	$C_{35}H_{50}N_4O_{23}$	Na^+	917.27580	1	69.92%	0.38%
A: 8 B: 6	14	$C_{36}H_{52}N_6O_{23}$	Na^+	959.29760	1	69.86%	0.38%
A: 9 B: 5	14	$C_{37}H_{53}N_5O_{24}$	Na^+	974.29727	1	72.89%	0.34%

Polymers grouped by monoisotopic mass

Composition				Monoisotopic mass	Quantity
A: 1 B: 2<br>A: 1 B: 2	3<br>3	$C_7H_{12}N_2O_5$<br>$C_7H_{12}N_2O_5$	Na^+<br>H^+	204.0746	0.53%
A: 2 B: 1<br>A: 2 B: 1	3<br>3	$C_8H_{13}NO_6$<br>$C_8H_{13}NO_6$	Na^+<br>H^+	219.0743	0.66%
A: 2 B: 2<br>A: 2 B: 2	4<br>4	$C_{10}H_{16}N_2O_7$<br>$C_{10}H_{16}N_2O_7$	Na^+<br>K^+	276.0958	1.27%
A: 3 B: 1	4	$C_{11}H_{17}NO_8$	Na^+	291.0954	0.83%
A: 4 B: 0	4	$C_{12}H_{18}O_9$	Na^+	306.0951	0.26%
A: 2 B: 3	5	$C_{12}H_{19}N_3O_8$	Na^+	333.1172	0.81%
A: 3 B: 2<br>A: 3 B: 2	5<br>5	$C_{13}H_{20}N_2O_9$<br>$C_{13}H_{20}N_2O_9$	Na^+<br>K^+	348.1169	1.72%
A: 0 B: 6	6	$C_{12}H_{20}N_6O_7$	H^+	360.1393	0.76%
A: 4 B: 1	5	$C_{14}H_{21}NO_{10}$	Na^+	363.1165	0.85%
A: 2 B: 4	6	$C_{14}H_{22}N_4O_9$	Na^+	390.1387	0.46%
A: 3 B: 3	6	$C_{15}H_{23}N_3O_{10}$	Na^+	405.1383	1.44%
A: 4 B: 2<br>A: 4 B: 2	6<br>6	$C_{16}H_{24}N_2O_{11}$<br>$C_{16}H_{24}N_2O_{11}$	Na^+<br>K^+	420.1380	2.13%
A: 5 B: 1	6	$C_{17}H_{25}NO_{12}$	Na^+	435.1377	0.78%
A: 2 B: 5	7	$C_{16}H_{25}N_5O_{10}$	K^+	447.1601	0.43%
A: 3 B: 4<br>A: 3 B: 4	7<br>7	$C_{17}H_{26}N_4O_{11}$<br>$C_{17}H_{26}N_4O_{11}$	Na^+<br>K^+	462.1598	1.66%
A: 4 B: 3<br>A: 4 B: 3	7<br>7	$C_{18}H_{27}N_3O_{12}$<br>$C_{18}H_{27}N_3O_{12}$	Na^+<br>K^+	477.1595	2.63%
A: 5 B: 2<br>A: 5 B: 2	7<br>7	$C_{19}H_{28}N_2O_{13}$<br>$C_{19}H_{28}N_2O_{13}$	Na^+<br>K^+	492.1591	2.05%

			Composition	Monoisotopic mass	Quantity
A: 6	B: 1	7	$C_{20}H_{29}NO_{14}$ Na^+	507.1588	0.62%
A: 3	B: 5	8	$C_{19}H_{29}N_5O_{12}$ K^+	519.1813	1.05%
A: 3	B: 5	8	$C_{19}H_{29}N_5O_{12}$ Na^+		
A: 4	B: 4	8	$C_{20}H_{30}N_4O_{13}$ Na^+	534.1809	2.05%
A: 4	B: 4	8	$C_{20}H_{30}N_4O_{13}$ K^+		
A: 5	B: 3	8	$C_{21}H_{31}N_3O_{14}$ Na^+	549.1806	2.47%
A: 5	B: 3	8	$C_{21}H_{31}N_3O_{14}$ K^+		
A: 6	B: 2	8	$C_{22}H_{32}N_2O_{15}$ Na^+	564.1803	1.23%
A: 3	B: 6	9	$C_{21}H_{32}N_6O_{13}$ K^+	576.2027	0.44%
A: 7	B: 1	8	$C_{23}H_{33}NO_{16}$ Na^+	579.1799	0.50%
A: 4	B: 5	9	$C_{22}H_{33}N_5O_{14}$ K^+	591.2024	1.37%
A: 4	B: 5	9	$C_{22}H_{33}N_5O_{14}$ Na^+		
A: 5	B: 4	9	$C_{23}H_{34}N_4O_{15}$ Na^+	606.2021	2.07%
A: 5	B: 4	9	$C_{23}H_{34}N_4O_{15}$ K^+		
A: 6	B: 3	9	$C_{24}H_{35}N_3O_{16}$ Na^+	621.2017	1.93%
A: 6	B: 3	9	$C_{24}H_{35}N_3O_{16}$ K^+		
A: 7	B: 2	9	$C_{25}H_{36}N_2O_{17}$ Na^+	636.2014	0.75%
A: 4	B: 6	10	$C_{24}H_{36}N_6O_{15}$ K^+	648.2239	0.92%
A: 4	B: 6	10	$C_{24}H_{36}N_6O_{15}$ Na^+		
A: 1	B: 10	11	$C_{23}H_{36}N_{10}O_{13}$ K^+	660.2463	1.62%
A: 5	B: 5	10	$C_{25}H_{37}N_5O_{16}$ Na^+	663.2235	1.50%
A: 5	B: 5	10	$C_{25}H_{37}N_5O_{16}$ K^+		
A: 6	B: 4	10	$C_{26}H_{38}N_4O_{17}$ Na^+	678.2232	1.90%
A: 6	B: 4	10	$C_{26}H_{38}N_4O_{17}$ K^+		
A: 7	B: 3	10	$C_{27}H_{39}N_3O_{18}$ Na^+	693.2229	0.95%
A: 8	B: 2	10	$C_{28}H_{40}N_2O_{19}$ Na^+	708.2225	0.53%
A: 5	B: 6	11	$C_{27}H_{40}N_6O_{17}$ K^+	720.2450	1.01%
A: 5	B: 6	11	$C_{27}H_{40}N_6O_{17}$ Na^+		
A: 6	B: 5	11	$C_{28}H_{41}N_5O_{18}$ Na^+	735.2447	1.25%
A: 6	B: 5	11	$C_{28}H_{41}N_5O_{18}$ K^+		
A: 7	B: 4	11	$C_{29}H_{42}N_4O_{19}$ Na^+	750.2443	1.26%
A: 7	B: 4	11	$C_{29}H_{42}N_4O_{19}$ K^+		
A: 8	B: 3	11	$C_{30}H_{43}N_3O_{20}$ Na^+	765.2440	0.61%
A: 9	B: 2	11	$C_{31}H_{44}N_2O_{21}$ Na^+	780.2437	0.38%
A: 6	B: 6	12	$C_{30}H_{44}N_6O_{19}$ K^+	792.2661	0.87%
A: 6	B: 6	12	$C_{30}H_{44}N_6O_{19}$ Na^+		

Composition				Monoisotopic mass	Quantity
A: 7	B: 5	12	$C_{31}H_{45}N_5O_{20}$ Na^+	807.2658	1.01%
A: 7	B: 5	12	$C_{31}H_{45}N_5O_{20}$ K^+		
A: 8	B: 4	12	$C_{32}H_{46}N_4O_{21}$ Na^+	822.2655	0.60%
A: 9	B: 3	12	$C_{33}H_{47}N_3O_{22}$ Na^+	837.2651	0.38%
A: 7	B: 6	13	$C_{33}H_{48}N_6O_{21}$ Na^+	864.2873	0.43%
A: 8	B: 5	13	$C_{34}H_{49}N_5O_{22}$ Na^+	879.2869	0.50%
A: 9	B: 4	13	$C_{35}H_{50}N_4O_{23}$ Na^+	894.2866	0.38%
A: 8	B: 6	14	$C_{36}H_{52}N_6O_{23}$ Na^+	936.3084	0.38%
A: 9	B: 5	14	$C_{37}H_{53}N_5O_{24}$ Na^+	951.3080	0.34%

Analytical report

Page under construction...

aG MALDI, 12cyc

Analysis parameters

Ionizations

MF	Monoisotopic mass	m/z mass
Na^{+}	22.98977	22.98922
K^{+}	38.96371	38.96316
H^{+}	1.00783	1.00728

End groups

α	ω	Color
H	OH	

Monomers

	Description	mf	Monoisotopic mass	min	max
A	lactic acid	$C_3H_4O_2$	72.02113	0	100
B	glycine	C_2H_3NO	57.02146	0	100

Experimental spectrum

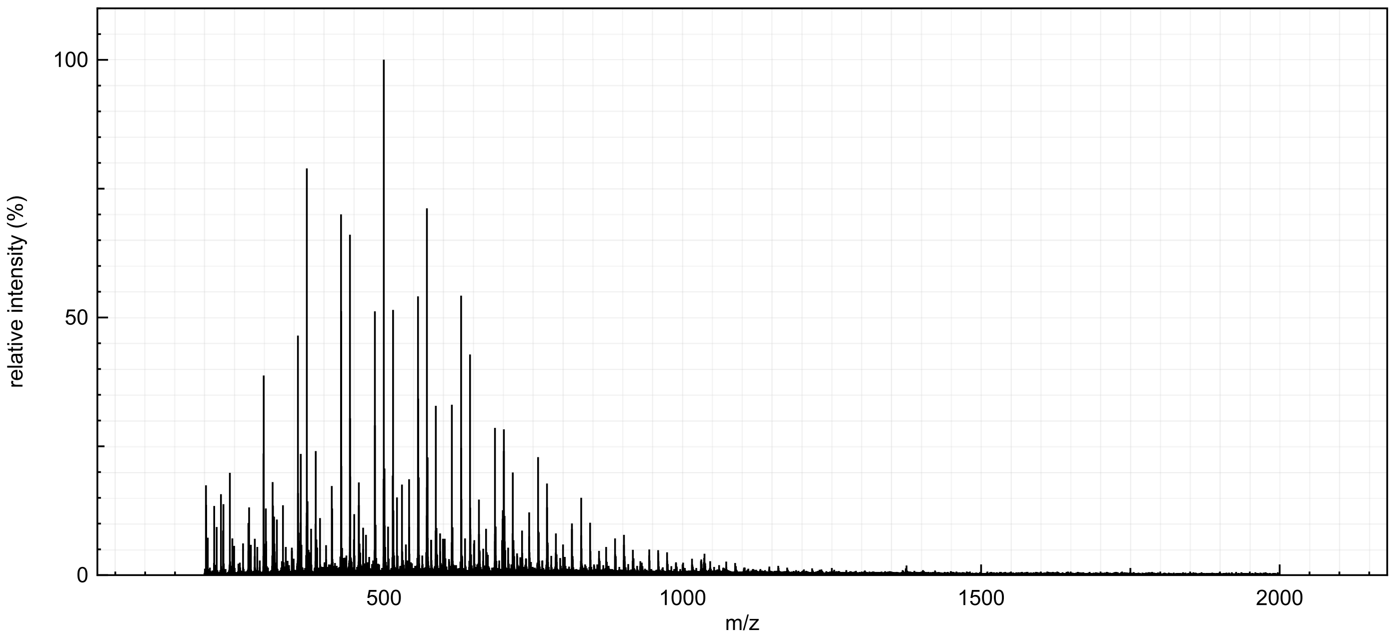

Reconstructed spectrum

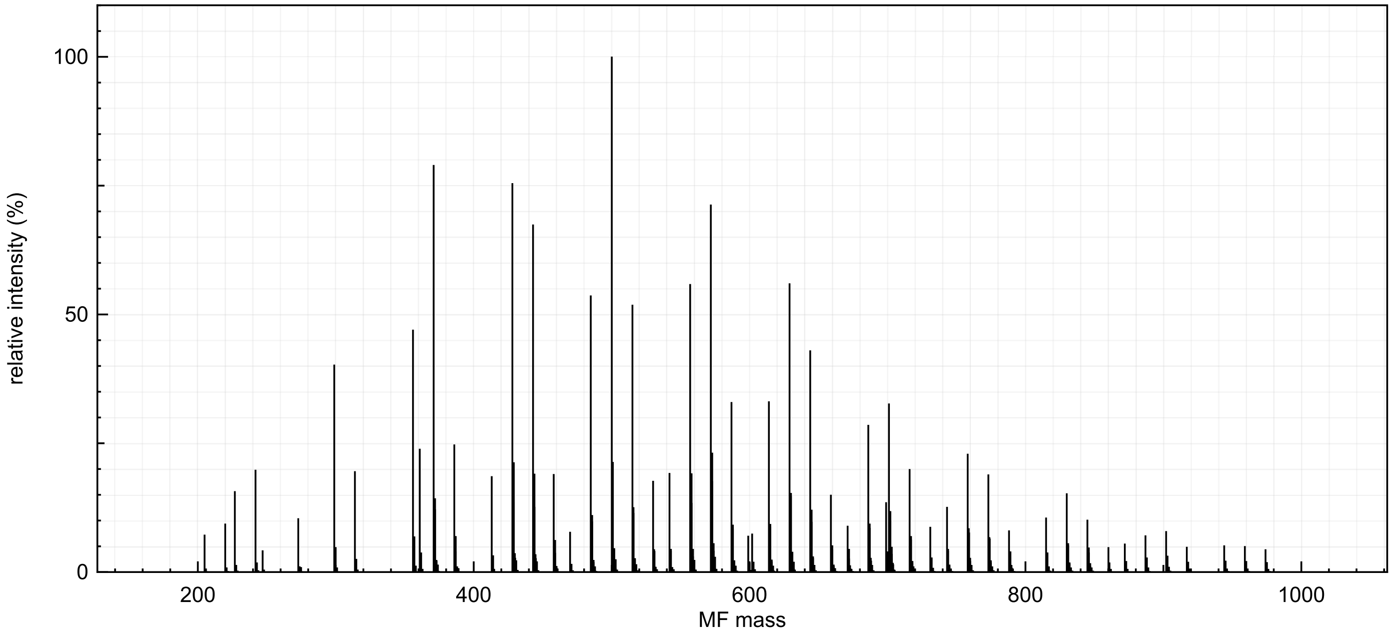

Error (ppm) versus m/z

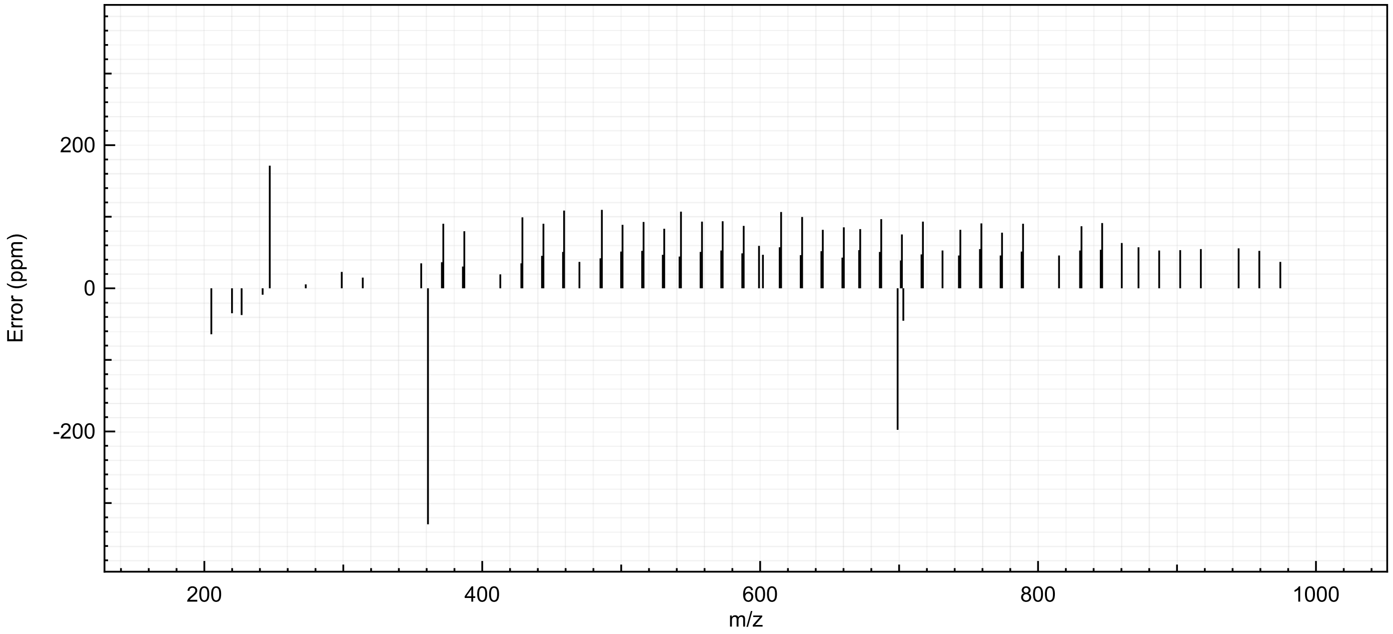

Analysis result

Percent of peaks covered: 55.21%
Number of assigned peaks: 80

Polymers [m/z]

Composition	Nb units	MF	Ioniz.	em [m/z]	Charge	Similarity	Quantity
A: 1 B: 2	3	$C_7H_{12}N_2O_5$	H^+	205.08190	1	52.46%	0.22%
A: 1 B: 2	3	$C_7H_{12}N_2O_5$	Na^+	227.06384	1	44.94%	0.51%
A: 2 B: 1	3	$C_8H_{13}NO_6$	H^+	220.08156	1	68.79%	0.20%
A: 2 B: 1	3	$C_8H_{13}NO_6$	Na^+	242.06351	1	63.00%	0.48%
A: 3 B: 0	3	$C_9H_{14}O_7$	K^+	273.03711	1	45.92%	0.54%
A: 0 B: 4	4	$C_8H_{14}N_4O_5$	H^+	247.10370	1	41.01%	0.22%
A: 2 B: 2	4	$C_{10}H_{16}N_2O_7$	Na^+	299.08497	1	67.84%	1.15%
A: 3 B: 1	4	$C_{11}H_{17}NO_8$	Na^+	314.08464	1	50.68%	0.88%
A: 2 B: 3	5	$C_{12}H_{19}N_3O_8$	Na^+	356.10644	1	74.53%	1.07%
A: 2 B: 3	5	$C_{12}H_{19}N_3O_8$	K^+	372.08037	1	60.93%	0.56%
A: 3 B: 2	5	$C_{13}H_{20}N_2O_9$	Na^+	371.10610	1	81.76%	1.49%
A: 3 B: 2	5	$C_{13}H_{20}N_2O_9$	K^+	387.08004	1	51.91%	0.39%
A: 0 B: 6	6	$C_{12}H_{20}N_6O_7$	H^+	361.14662	1	72.80%	0.70%
A: 4 B: 1	5	$C_{14}H_{21}NO_{10}$	Na^+	386.10577	1	68.23%	0.69%
A: 2 B: 4	6	$C_{14}H_{22}N_4O_9$	Na^+	413.12790	1	75.06%	0.58%
A: 2 B: 4	6	$C_{14}H_{22}N_4O_9$	K^+	429.10184	1	66.64%	0.76%
A: 3 B: 3	6	$C_{15}H_{23}N_3O_{10}$	Na^+	428.12756	1	83.03%	1.89%
A: 3 B: 3	6	$C_{15}H_{23}N_3O_{10}$	K^+	444.10150	1	68.10%	0.65%
A: 4 B: 2	6	$C_{16}H_{24}N_2O_{11}$	Na^+	443.12723	1	82.95%	1.56%
A: 4 B: 2	6	$C_{16}H_{24}N_2O_{11}$	K^+	459.10117	1	54.16%	0.41%
A: 5 B: 1	6	$C_{17}H_{25}NO_{12}$	Na^+	458.12690	1	67.30%	0.71%
A: 2 B: 5	7	$C_{16}H_{25}N_5O_{10}$	Na^+	470.14936	1	57.60%	0.38%
A: 2 B: 5	7	$C_{16}H_{25}N_5O_{10}$	K^+	486.12330	1	64.80%	0.51%
A: 3 B: 4	7	$C_{17}H_{26}N_4O_{11}$	Na^+	485.14903	1	85.48%	1.29%
A: 3 B: 4	7	$C_{17}H_{26}N_4O_{11}$	K^+	501.12297	1	71.17%	0.81%
A: 4 B: 3	7	$C_{18}H_{27}N_3O_{12}$	Na^+	500.14869	1	86.24%	2.00%
A: 4 B: 3	7	$C_{18}H_{27}N_3O_{12}$	K^+	516.12263	1	67.97%	0.56%
A: 5 B: 2	7	$C_{19}H_{28}N_2O_{13}$	Na^+	515.14836	1	83.96%	1.24%
A: 5 B: 2	7	$C_{19}H_{28}N_2O_{13}$	K^+	531.12230	1	52.38%	0.39%

Composition	Nb units	MF	Ioniz.	em [m/z]	Charge	Similarity	Quantity
A: 2 B: 6	8	$C_{18}H_{28}N_6O_{11}$	K^+	543.14476	1	52.18%	0.38%
A: 6 B: 1	7	$C_{20}H_{29}NO_{14}$	Na^+	530.14803	1	65.81%	0.60%
A: 3 B: 5	8	$C_{19}H_{29}N_5O_{12}$	Na^+	542.17049	1	72.47%	0.62%
A: 3 B: 5	8	$C_{19}H_{29}N_5O_{12}$	K^+	558.14443	1	75.74%	0.62%
A: 4 B: 4	8	$C_{20}H_{30}N_4O_{13}$	Na^+	557.17016	1	87.31%	1.49%
A: 4 B: 4	8	$C_{20}H_{30}N_4O_{13}$	K^+	573.14409	1	73.88%	0.73%
A: 5 B: 3	8	$C_{21}H_{31}N_3O_{14}$	Na^+	572.16982	1	84.19%	1.60%
A: 5 B: 3	8	$C_{21}H_{31}N_3O_{14}$	K^+	588.14376	1	66.49%	0.50%
A: 6 B: 2	8	$C_{22}H_{32}N_2O_{15}$	Na^+	587.16949	1	76.35%	0.92%
A: 3 B: 6	9	$C_{21}H_{32}N_6O_{13}$	Na^+	599.19196	1	50.31%	0.59%
A: 3 B: 6	9	$C_{21}H_{32}N_6O_{13}$	K^+	615.16589	1	63.47%	0.51%
A: 7 B: 1	8	$C_{23}H_{33}NO_{16}$	Na^+	602.16915	1	62.29%	0.46%
A: 4 B: 5	9	$C_{22}H_{33}N_5O_{14}$	Na^+	614.19162	1	78.17%	0.94%
A: 4 B: 5	9	$C_{22}H_{33}N_5O_{14}$	K^+	630.16556	1	73.29%	0.66%
A: 5 B: 4	9	$C_{23}H_{34}N_4O_{15}$	Na^+	629.19129	1	84.96%	1.39%
A: 5 B: 4	9	$C_{23}H_{34}N_4O_{15}$	K^+	645.16522	1	70.81%	0.56%
A: 6 B: 3	9	$C_{24}H_{35}N_3O_{16}$	Na^+	644.19095	1	82.27%	1.13%
A: 6 B: 3	9	$C_{24}H_{35}N_3O_{16}$	K^+	660.16489	1	62.90%	0.44%
A: 3 B: 7	10	$C_{23}H_{35}N_7O_{14}$	K^+	672.18736	1	53.59%	0.45%
A: 7 B: 2	9	$C_{25}H_{36}N_2O_{17}$	Na^+	659.19062	1	71.29%	0.64%
A: 4 B: 6	10	$C_{24}H_{36}N_6O_{15}$	Na^+	671.21309	1	59.56%	0.55%
A: 4 B: 6	10	$C_{24}H_{36}N_6O_{15}$	K^+	687.18702	1	69.05%	0.50%
A: 1 B: 10	11	$C_{23}H_{36}N_{10}O_{13}$	K^+	699.20949	1	44.72%	1.53%
A: 5 B: 5	10	$C_{25}H_{37}N_5O_{16}$	Na^+	686.21275	1	79.46%	0.88%
A: 5 B: 5	10	$C_{25}H_{37}N_5O_{16}$	K^+	702.18669	1	75.07%	0.67%
A: 6 B: 4	10	$C_{26}H_{38}N_4O_{17}$	Na^+	701.21242	1	80.86%	1.21%
A: 6 B: 4	10	$C_{26}H_{38}N_4O_{17}$	K^+	717.18635	1	70.95%	0.48%
A: 7 B: 3	10	$C_{27}H_{39}N_3O_{18}$	Na^+	716.21208	1	77.00%	0.78%
A: 0 B: 12	12	$C_{24}H_{38}N_{12}O_{13}$	H^+	703.27541	1	57.55%	0.44%
A: 4 B: 7	11	$C_{26}H_{39}N_7O_{16}$	K^+	744.20849	1	62.35%	0.44%
A: 8 B: 2	10	$C_{28}H_{40}N_2O_{19}$	Na^+	731.21175	1	66.33%	0.48%
A: 5 B: 6	11	$C_{27}H_{40}N_6O_{17}$	Na^+	743.23421	1	68.35%	0.64%
A: 5 B: 6	11	$C_{27}H_{40}N_6O_{17}$	K^+	759.20815	1	69.92%	0.50%
A: 6 B: 5	11	$C_{28}H_{41}N_5O_{18}$	Na^+	758.23388	1	77.85%	0.82%

Composition	Nb units	MF	Ioniz.	em [m/z]	Charge	Similarity	Quantity
A: 6 B: 5	11	$C_{28}H_{41}N_5O_{18}$	K^+	774.20782	1	70.64%	0.40%
A: 7 B: 4	11	$C_{29}H_{42}N_4O_{19}$	Na^+	773.23355	1	80.70%	0.71%
A: 7 B: 4	11	$C_{29}H_{42}N_4O_{19}$	K^+	789.20748	1	70.75%	0.35%
A: 8 B: 3	11	$C_{30}H_{43}N_3O_{20}$	Na^+	788.23321	1	69.62%	0.49%
A: 6 B: 6	12	$C_{30}H_{44}N_6O_{19}$	Na^+	815.25534	1	76.12%	0.51%
A: 6 B: 6	12	$C_{30}H_{44}N_6O_{19}$	K^+	831.22928	1	68.57%	0.38%
A: 7 B: 5	12	$C_{31}H_{45}N_5O_{20}$	Na^+	830.25501	1	76.97%	0.58%
A: 7 B: 5	12	$C_{31}H_{45}N_5O_{20}$	K^+	846.22895	1	68.25%	0.34%
A: 8 B: 4	12	$C_{32}H_{46}N_4O_{21}$	Na^+	845.25468	1	73.48%	0.49%
A: 9 B: 3	12	$C_{33}H_{47}N_3O_{22}$	Na^+	860.25434	1	66.18%	0.35%
A: 6 B: 7	13	$C_{32}H_{47}N_7O_{20}$	Na^+	872.27681	1	62.43%	0.40%
A: 7 B: 6	13	$C_{33}H_{48}N_6O_{21}$	Na^+	887.27647	1	74.56%	0.42%
A: 8 B: 5	13	$C_{34}H_{49}N_5O_{22}$	Na^+	902.27614	1	77.97%	0.44%
A: 9 B: 4	13	$C_{35}H_{50}N_4O_{23}$	Na^+	917.27580	1	65.40%	0.35%
A: 7 B: 7	14	$C_{35}H_{51}N_7O_{22}$	Na^+	944.29794	1	69.62%	0.33%
A: 8 B: 6	14	$C_{36}H_{52}N_6O_{23}$	Na^+	959.29760	1	73.84%	0.38%
A: 9 B: 5	14	$C_{37}H_{53}N_5O_{24}$	Na^+	974.29727	1	72.96%	0.32%

Polymers grouped by monoisotopic mass

Composition				Monoisotopic mass	Quantity
A: 1 B: 2<br>A: 1 B: 2	3<br>3	$C_7H_{12}N_2O_5$<br>$C_7H_{12}N_2O_5$	H^+<br>Na^+	204.0746	0.73%
A: 2 B: 1<br>A: 2 B: 1	3<br>3	$C_8H_{13}NO_6$<br>$C_8H_{13}NO_6$	H^+<br>Na^+	219.0743	0.68%
A: 3 B: 0	3	$C_9H_{14}O_7$	K^+	234.0740	0.54%
A: 0 B: 4	4	$C_8H_{14}N_4O_5$	H^+	246.0964	0.22%
A: 2 B: 2	4	$C_{10}H_{16}N_2O_7$	Na^+	276.0958	1.15%
A: 3 B: 1	4	$C_{11}H_{17}NO_8$	Na^+	291.0954	0.88%
A: 2 B: 3<br>A: 2 B: 3	5<br>5	$C_{12}H_{19}N_3O_8$<br>$C_{12}H_{19}N_3O_8$	Na^+<br>K^+	333.1172	1.63%
A: 3 B: 2<br>A: 3 B: 2	5<br>5	$C_{13}H_{20}N_2O_9$<br>$C_{13}H_{20}N_2O_9$	Na^+<br>K^+	348.1169	1.88%
A: 0 B: 6	6	$C_{12}H_{20}N_6O_7$	H^+	360.1393	0.70%
A: 4 B: 1	5	$C_{14}H_{21}NO_{10}$	Na^+	363.1165	0.69%

Composition				Monoisotopic mass	Quantity
A: 2	B: 4	6	$C_{14}H_{22}N_4O_9$ Na^+	390.1387	1.34%
A: 2	B: 4	6	$C_{14}H_{22}N_4O_9$ K^+		
A: 3	B: 3	6	$C_{15}H_{23}N_3O_{10}$ Na^+	405.1383	2.53%
A: 3	B: 3	6	$C_{15}H_{23}N_3O_{10}$ K^+		
A: 4	B: 2	6	$C_{16}H_{24}N_2O_{11}$ Na^+	420.1380	1.97%
A: 4	B: 2	6	$C_{16}H_{24}N_2O_{11}$ K^+		
A: 5	B: 1	6	$C_{17}H_{25}NO_{12}$ Na^+	435.1377	0.71%
A: 2	B: 5	7	$C_{16}H_{25}N_5O_{10}$ K^+	447.1601	0.89%
A: 2	B: 5	7	$C_{16}H_{25}N_5O_{10}$ Na^+		
A: 3	B: 4	7	$C_{17}H_{26}N_4O_{11}$ Na^+	462.1598	2.10%
A: 3	B: 4	7	$C_{17}H_{26}N_4O_{11}$ K^+		
A: 4	B: 3	7	$C_{18}H_{27}N_3O_{12}$ Na^+	477.1595	2.56%
A: 4	B: 3	7	$C_{18}H_{27}N_3O_{12}$ K^+		
A: 5	B: 2	7	$C_{19}H_{28}N_2O_{13}$ Na^+	492.1591	1.64%
A: 5	B: 2	7	$C_{19}H_{28}N_2O_{13}$ K^+		
A: 2	B: 6	8	$C_{18}H_{28}N_6O_{11}$ K^+	504.1816	0.38%
A: 6	B: 1	7	$C_{20}H_{29}NO_{14}$ Na^+	507.1588	0.60%
A: 3	B: 5	8	$C_{19}H_{29}N_5O_{12}$ K^+	519.1813	1.24%
A: 3	B: 5	8	$C_{19}H_{29}N_5O_{12}$ Na^+		
A: 4	B: 4	8	$C_{20}H_{30}N_4O_{13}$ Na^+	534.1809	2.22%
A: 4	B: 4	8	$C_{20}H_{30}N_4O_{13}$ K^+		
A: 5	B: 3	8	$C_{21}H_{31}N_3O_{14}$ Na^+	549.1806	2.11%
A: 5	B: 3	8	$C_{21}H_{31}N_3O_{14}$ K^+		
A: 6	B: 2	8	$C_{22}H_{32}N_2O_{15}$ Na^+	564.1803	0.92%
A: 3	B: 6	9	$C_{21}H_{32}N_6O_{13}$ K^+	576.2027	1.10%
A: 3	B: 6	9	$C_{21}H_{32}N_6O_{13}$ Na^+		
A: 7	B: 1	8	$C_{23}H_{33}NO_{16}$ Na^+	579.1799	0.46%
A: 4	B: 5	9	$C_{22}H_{33}N_5O_{14}$ Na^+	591.2024	1.60%
A: 4	B: 5	9	$C_{22}H_{33}N_5O_{14}$ K^+		
A: 5	B: 4	9	$C_{23}H_{34}N_4O_{15}$ Na^+	606.2021	1.95%
A: 5	B: 4	9	$C_{23}H_{34}N_4O_{15}$ K^+		
A: 6	B: 3	9	$C_{24}H_{35}N_3O_{16}$ Na^+	621.2017	1.56%
A: 6	B: 3	9	$C_{24}H_{35}N_3O_{16}$ K^+		
A: 3	B: 7	10	$C_{23}H_{35}N_7O_{14}$ K^+	633.2242	0.45%
A: 7	B: 2	9	$C_{25}H_{36}N_2O_{17}$ Na^+	636.2014	0.64%

Composition				Monoisotopic mass	Quantity
A: 4	B: 6	10	$C_{24}H_{36}N_{6}O_{15}\,K^{+}$	648.2239	1.05%
A: 4	B: 6	10	$C_{24}H_{36}N_{6}O_{15}\,Na^{+}$		
A: 1	B: 10	11	$C_{23}H_{36}N_{10}O_{13}\,K^{+}$	660.2463	1.53%
A: 5	B: 5	10	$C_{25}H_{37}N_{5}O_{16}\,Na^{+}$	663.2235	1.55%
A: 5	B: 5	10	$C_{25}H_{37}N_{5}O_{16}\,K^{+}$		
A: 6	B: 4	10	$C_{26}H_{38}N_{4}O_{17}\,Na^{+}$	678.2232	1.69%
A: 6	B: 4	10	$C_{26}H_{38}N_{4}O_{17}\,K^{+}$		
A: 7	B: 3	10	$C_{27}H_{39}N_{3}O_{18}\,Na^{+}$	693.2229	0.78%
A: 0	B: 12	12	$C_{24}H_{38}N_{12}O_{13}\,H^{+}$	702.2681	0.44%
A: 4	B: 7	11	$C_{26}H_{39}N_{7}O_{16}\,K^{+}$	705.2453	0.44%
A: 8	B: 2	10	$C_{28}H_{40}N_{2}O_{19}\,Na^{+}$	708.2225	0.48%
A: 5	B: 6	11	$C_{27}H_{40}N_{6}O_{17}\,K^{+}$	720.2450	1.14%
A: 5	B: 6	11	$C_{27}H_{40}N_{6}O_{17}\,Na^{+}$		
A: 6	B: 5	11	$C_{28}H_{41}N_{5}O_{18}\,Na^{+}$	735.2447	1.22%
A: 6	B: 5	11	$C_{28}H_{41}N_{5}O_{18}\,K^{+}$		
A: 7	B: 4	11	$C_{29}H_{42}N_{4}O_{19}\,Na^{+}$	750.2443	1.06%
A: 7	B: 4	11	$C_{29}H_{42}N_{4}O_{19}\,K^{+}$		
A: 8	B: 3	11	$C_{30}H_{43}N_{3}O_{20}\,Na^{+}$	765.2440	0.49%
A: 6	B: 6	12	$C_{30}H_{44}N_{6}O_{19}\,Na^{+}$	792.2661	0.89%
A: 6	B: 6	12	$C_{30}H_{44}N_{6}O_{19}\,K^{+}$		
A: 7	B: 5	12	$C_{31}H_{45}N_{5}O_{20}\,Na^{+}$	807.2658	0.92%
A: 7	B: 5	12	$C_{31}H_{45}N_{5}O_{20}\,K^{+}$		
A: 8	B: 4	12	$C_{32}H_{46}N_{4}O_{21}\,Na^{+}$	822.2655	0.49%
A: 9	B: 3	12	$C_{33}H_{47}N_{3}O_{22}\,Na^{+}$	837.2651	0.35%
A: 6	B: 7	13	$C_{32}H_{47}N_{7}O_{20}\,Na^{+}$	849.2876	0.40%
A: 7	B: 6	13	$C_{33}H_{48}N_{6}O_{21}\,Na^{+}$	864.2873	0.42%
A: 8	B: 5	13	$C_{34}H_{49}N_{5}O_{22}\,Na^{+}$	879.2869	0.44%
A: 9	B: 4	13	$C_{35}H_{50}N_{4}O_{23}\,Na^{+}$	894.2866	0.35%
A: 7	B: 7	14	$C_{35}H_{51}N_{7}O_{22}\,Na^{+}$	921.3087	0.33%
A: 8	B: 6	14	$C_{36}H_{52}N_{6}O_{23}\,Na^{+}$	936.3084	0.38%
A: 9	B: 5	14	$C_{37}H_{53}N_{5}O_{24}\,Na^{+}$	951.3080	0.32%

Analytical report

Page under construction...

aA MALDI, 1cyc, no dust ctrl

Analysis parameters

Ionizations

MF	Monoisotopic mass	m/z mass
H^+	1.00783	1.00728
Na^+	22.98977	22.98922
K^+	38.96371	38.96316

End groups

α	ω	Color
H	OH	

Monomers

	Description	mf	Monoisotopic mass	min	max
A	Lactic acid	$C_3H_4O_2$	72.02113	0	100
B	Alanine	C_3H_5NO	71.03711	0	100

Experimental spectrum

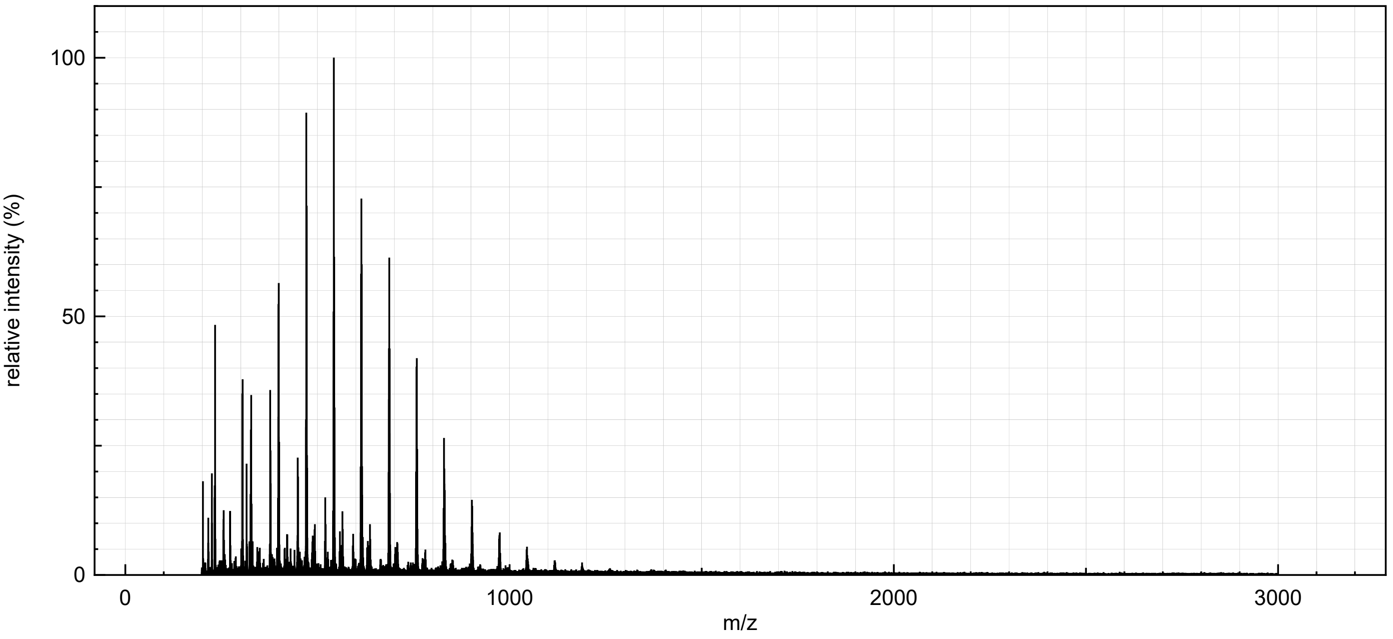

Reconstructed spectrum

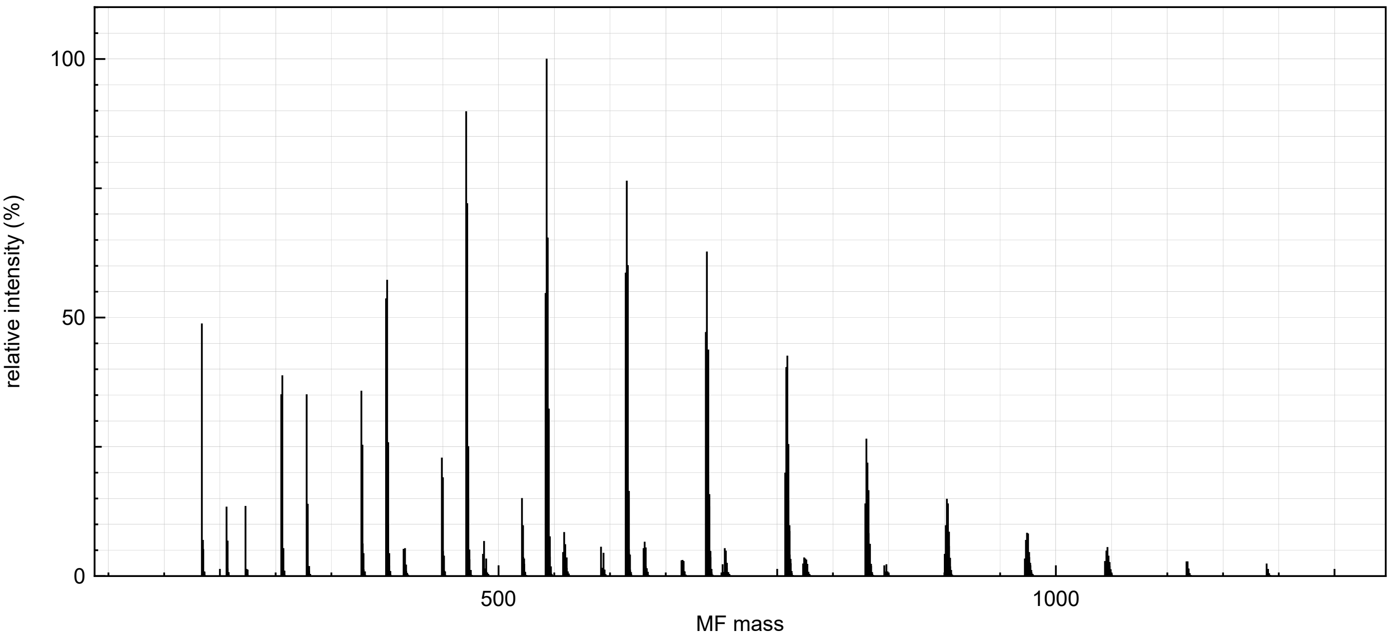

Error (ppm) versus m/z

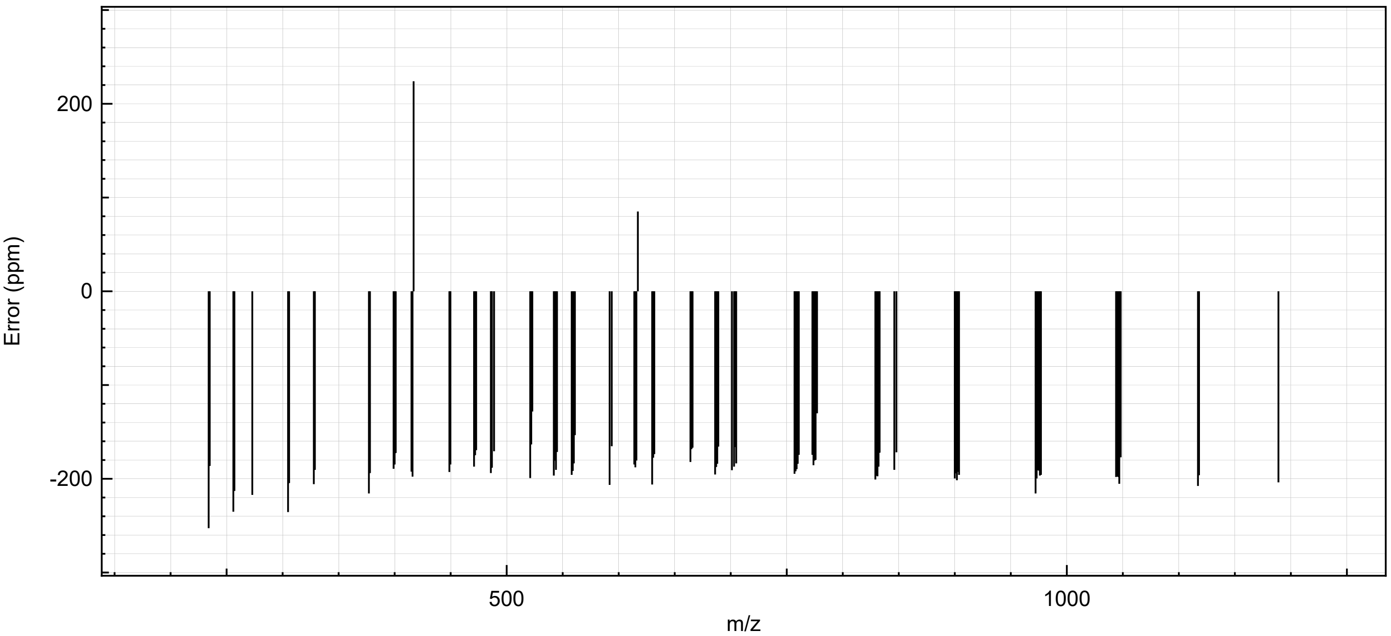

Analysis result

Percent of peaks covered: 50.29%
Number of assigned peaks: 92

Polymers [m/z]

Composition	Nb units	MF	Ioniz.	em [m/z]	Charge	Similarity	Quantity
A: 2 B: 1	3	$C_9H_{15}NO_6$	H^+	234.09721	1	91.98%	0.58%
A: 2 B: 1	3	$C_9H_{15}NO_6$	Na^+	256.07916	1	70.50%	0.31%
A: 3 B: 0	3	$C_9H_{14}O_7$	H^+	235.08123	1	71.61%	0.13%
A: 3 B: 0	3	$C_9H_{14}O_7$	Na^+	257.06317	1	55.81%	0.17%
A: 3 B: 0	3	$C_9H_{14}O_7$	K^+	273.03711	1	78.91%	0.32%
A: 2 B: 2	4	$C_{12}H_{20}N_2O_7$	H^+	305.13433	1	51.59%	0.90%
A: 3 B: 1	4	$C_{12}H_{19}NO_8$	H^+	306.11834	1	87.14%	0.61%
A: 3 B: 1	4	$C_{12}H_{19}NO_8$	Na^+	328.10029	1	78.14%	0.61%
A: 4 B: 0	4	$C_{12}H_{18}O_9$	Na^+	329.08430	1	75.82%	0.26%
A: 3 B: 2	5	$C_{15}H_{24}N_2O_9$	H^+	377.15546	1	61.70%	0.79%
A: 3 B: 2	5	$C_{15}H_{24}N_2O_9$	Na^+	399.13740	1	58.01%	1.56%
A: 3 B: 2	5	$C_{15}H_{24}N_2O_9$	K^+	415.11134	1	56.08%	0.24%
A: 4 B: 1	5	$C_{15}H_{23}NO_{10}$	H^+	378.13947	1	85.09%	0.49%
A: 4 B: 1	5	$C_{15}H_{23}NO_{10}$	Na^+	400.12142	1	78.36%	1.04%
A: 4 B: 1	5	$C_{15}H_{23}NO_{10}$	K^+	416.09535	1	63.09%	0.22%
A: 5 B: 0	5	$C_{15}H_{22}O_{11}$	Na^+	401.10543	1	78.57%	0.46%
A: 5 B: 0	5	$C_{15}H_{22}O_{11}$	K^+	417.07937	1	66.90%	0.16%
A: 3 B: 3	6	$C_{18}H_{29}N_3O_{10}$	K^+	486.14845	1	50.27%	0.28%
A: 4 B: 2	6	$C_{18}H_{28}N_2O_{11}$	H^+	449.17659	1	65.97%	0.54%
A: 4 B: 2	6	$C_{18}H_{28}N_2O_{11}$	Na^+	471.15853	1	63.34%	2.06%
A: 4 B: 2	6	$C_{18}H_{28}N_2O_{11}$	K^+	487.13247	1	60.53%	0.28%
A: 5 B: 1	6	$C_{18}H_{27}NO_{12}$	H^+	450.16060	1	79.01%	0.35%
A: 5 B: 1	6	$C_{18}H_{27}NO_{12}$	Na^+	472.14255	1	80.13%	1.32%
A: 6 B: 0	6	$C_{18}H_{26}O_{13}$	Na^+	473.12656	1	80.10%	0.62%
A: 6 B: 0	6	$C_{18}H_{26}O_{13}$	K^+	489.10050	1	65.43%	0.18%
A: 4 B: 3	7	$C_{21}H_{33}N_3O_{12}$	Na^+	542.19564	1	50.68%	2.45%
A: 4 B: 3	7	$C_{21}H_{33}N_3O_{12}$	K^+	558.16958	1	54.11%	0.28%
A: 5 B: 2	7	$C_{21}H_{32}N_2O_{13}$	H^+	521.19772	1	66.95%	0.35%
A: 5 B: 2	7	$C_{21}H_{32}N_2O_{13}$	Na^+	543.17966	1	69.94%	2.11%

Composition	Nb units	MF	Ioniz.	em [m/z]	Charge	Similarity	Quantity
A: 5 B: 2	7	$C_{21}H_{32}N_2O_{13}$	K^+	559.15360	1	65.11%	0.28%
A: 6 B: 1	7	$C_{21}H_{31}NO_{14}$	H^+	522.18173	1	75.78%	0.25%
A: 6 B: 1	7	$C_{21}H_{31}NO_{14}$	Na^+	544.16368	1	86.13%	1.24%
A: 6 B: 1	7	$C_{21}H_{31}NO_{14}$	K^+	560.13761	1	71.25%	0.23%
A: 7 B: 0	7	$C_{21}H_{30}O_{15}$	H^+	523.16575	1	60.68%	0.18%
A: 7 B: 0	7	$C_{21}H_{30}O_{15}$	Na^+	545.14769	1	84.48%	0.55%
A: 7 B: 0	7	$C_{21}H_{30}O_{15}$	K^+	561.12163	1	67.06%	0.19%
A: 5 B: 3	8	$C_{24}H_{37}N_3O_{14}$	H^+	592.23483	1	57.50%	0.29%
A: 5 B: 3	8	$C_{24}H_{37}N_3O_{14}$	Na^+	614.21677	1	55.15%	2.19%
A: 5 B: 3	8	$C_{24}H_{37}N_3O_{14}$	K^+	630.19071	1	61.15%	0.29%
A: 6 B: 2	8	$C_{24}H_{36}N_2O_{15}$	Na^+	615.20079	1	72.76%	1.89%
A: 6 B: 2	8	$C_{24}H_{36}N_2O_{15}$	K^+	631.17473	1	67.55%	0.26%
A: 7 B: 1	8	$C_{24}H_{35}NO_{16}$	H^+	594.20286	1	72.06%	0.18%
A: 7 B: 1	8	$C_{24}H_{35}NO_{16}$	Na^+	616.18480	1	81.32%	1.17%
A: 7 B: 1	8	$C_{24}H_{35}NO_{16}$	K^+	632.15874	1	72.41%	0.22%
A: 8 B: 0	8	$C_{24}H_{34}O_{17}$	Na^+	617.16882	1	79.33%	0.63%
A: 5 B: 4	9	$C_{27}H_{42}N_4O_{15}$	K^+	701.22782	1	56.63%	0.25%
A: 6 B: 3	9	$C_{27}H_{41}N_3O_{16}$	H^+	664.25596	1	62.99%	0.20%
A: 6 B: 3	9	$C_{27}H_{41}N_3O_{16}$	Na^+	686.23790	1	61.75%	1.76%
A: 7 B: 2	9	$C_{27}H_{40}N_2O_{17}$	H^+	665.23997	1	62.59%	0.19%
A: 7 B: 2	9	$C_{27}H_{40}N_2O_{17}$	Na^+	687.22192	1	78.61%	1.39%
A: 7 B: 2	9	$C_{27}H_{40}N_2O_{17}$	K^+	703.19586	1	68.92%	0.25%
A: 8 B: 1	9	$C_{27}H_{39}NO_{18}$	H^+	666.22399	1	65.29%	0.17%
A: 8 B: 1	9	$C_{27}H_{39}NO_{18}$	Na^+	688.20593	1	86.35%	0.81%
A: 8 B: 1	9	$C_{27}H_{39}NO_{18}$	K^+	704.17987	1	69.91%	0.23%
A: 9 B: 0	9	$C_{27}H_{38}O_{19}$	Na^+	689.18995	1	79.70%	0.42%
A: 9 B: 0	9	$C_{27}H_{38}O_{19}$	K^+	705.16389	1	64.55%	0.21%
A: 6 B: 4	10	$C_{30}H_{46}N_4O_{17}$	Na^+	757.27502	1	52.07%	1.18%
A: 6 B: 4	10	$C_{30}H_{46}N_4O_{17}$	K^+	773.24895	1	65.36%	0.23%
A: 7 B: 3	10	$C_{30}H_{45}N_3O_{18}$	Na^+	758.25903	1	67.02%	1.26%
A: 7 B: 3	10	$C_{30}H_{45}N_3O_{18}$	K^+	774.23297	1	69.04%	0.23%
A: 8 B: 2	10	$C_{30}H_{44}N_2O_{19}$	Na^+	759.24305	1	78.96%	0.98%
A: 8 B: 2	10	$C_{30}H_{44}N_2O_{19}$	K^+	775.21699	1	69.73%	0.22%
A: 9 B: 1	10	$C_{30}H_{43}NO_{20}$	Na^+	760.22706	1	84.75%	0.61%

Composition	Nb units	MF	Ioniz.	em [m/z]	Charge	Similarity	Quantity
A: 9 B: 1	10	$C_{30}H_{43}NO_{20}$	K^+	776.20100	1	67.54%	0.21%
A: 10 B: 0	10	$C_{30}H_{42}O_{21}$	Na^+	761.21108	1	78.47%	0.36%
A: 10 B: 0	10	$C_{30}H_{42}O_{21}$	K^+	777.18502	1	65.89%	0.22%
A: 7 B: 4	11	$C_{33}H_{50}N_4O_{19}$	Na^+	829.29615	1	58.17%	0.83%
A: 8 B: 3	11	$C_{33}H_{49}N_3O_{20}$	Na^+	830.28016	1	69.37%	0.86%
A: 8 B: 3	11	$C_{33}H_{49}N_3O_{20}$	K^+	846.25410	1	70.30%	0.18%
A: 9 B: 2	11	$C_{33}H_{48}N_2O_{21}$	Na^+	831.26418	1	80.58%	0.67%
A: 10 B: 1	11	$C_{33}H_{47}NO_{22}$	Na^+	832.24819	1	85.30%	0.44%
A: 10 B: 1	11	$C_{33}H_{47}NO_{22}$	K^+	848.22213	1	70.32%	0.17%
A: 11 B: 0	11	$C_{33}H_{46}O_{23}$	Na^+	833.23221	1	77.24%	0.28%
A: 7 B: 5	12	$C_{36}H_{55}N_5O_{20}$	Na^+	900.33326	1	55.50%	0.47%
A: 8 B: 4	12	$C_{36}H_{54}N_4O_{21}$	Na^+	901.31728	1	64.81%	0.59%
A: 9 B: 3	12	$C_{36}H_{53}N_3O_{22}$	Na^+	902.30129	1	73.10%	0.56%
A: 10 B: 2	12	$C_{36}H_{52}N_2O_{23}$	Na^+	903.28531	1	83.53%	0.44%
A: 11 B: 1	12	$C_{36}H_{51}NO_{24}$	Na^+	904.26932	1	85.60%	0.29%
A: 8 B: 5	13	$C_{39}H_{59}N_5O_{22}$	Na^+	972.35439	1	61.00%	0.34%
A: 9 B: 4	13	$C_{39}H_{58}N_4O_{23}$	Na^+	973.33840	1	69.09%	0.39%
A: 10 B: 3	13	$C_{39}H_{57}N_3O_{24}$	Na^+	974.32242	1	78.00%	0.37%
A: 11 B: 2	13	$C_{39}H_{56}N_2O_{25}$	Na^+	975.30644	1	83.00%	0.28%
A: 12 B: 1	13	$C_{39}H_{55}NO_{26}$	Na^+	976.29045	1	84.76%	0.20%
A: 13 B: 0	13	$C_{39}H_{54}O_{27}$	Na^+	977.27447	1	81.50%	0.15%
A: 9 B: 5	14	$C_{42}H_{63}N_5O_{24}$	Na^+	1044.37552	1	67.35%	0.26%
A: 10 B: 4	14	$C_{42}H_{62}N_4O_{25}$	Na^+	1045.35953	1	74.24%	0.28%
A: 11 B: 3	14	$C_{42}H_{61}N_3O_{26}$	Na^+	1046.34355	1	81.67%	0.26%
A: 12 B: 2	14	$C_{42}H_{60}N_2O_{27}$	Na^+	1047.32757	1	84.15%	0.21%
A: 13 B: 1	14	$C_{42}H_{59}NO_{28}$	Na^+	1048.31158	1	82.29%	0.16%
A: 11 B: 4	15	$C_{45}H_{66}N_4O_{27}$	Na^+	1117.38066	1	76.71%	0.19%
A: 12 B: 3	15	$C_{45}H_{65}N_3O_{28}$	Na^+	1118.36468	1	82.22%	0.18%
A: 12 B: 4	16	$C_{48}H_{70}N_4O_{29}$	Na^+	1189.40179	1	86.49%	0.14%

Polymers grouped by monoisotopic mass

Composition				Monoisotopic mass	Quantity
A: 2 B: 1	3	$C_9H_{15}NO_6$	H^+	233.0899	0.89%
A: 2 B: 1	3	$C_9H_{15}NO_6$	Na^+		

Composition				Monoisotopic mass	Quantity
A: 3 B: 0	3	$C_9H_{14}O_7$	K^+		
A: 3 B: 0	3	$C_9H_{14}O_7$	H^+	234.0740	0.63%
A: 3 B: 0	3	$C_9H_{14}O_7$	Na^+		
A: 2 B: 2	4	$C_{12}H_{20}N_2O_7$	H^+	304.1271	0.90%
A: 3 B: 1	4	$C_{12}H_{19}NO_8$	H^+	305.1111	1.22%
A: 3 B: 1	4	$C_{12}H_{19}NO_8$	Na^+		
A: 4 B: 0	4	$C_{12}H_{18}O_9$	Na^+	306.0951	0.26%
A: 3 B: 2	5	$C_{15}H_{24}N_2O_9$	H^+		
A: 3 B: 2	5	$C_{15}H_{24}N_2O_9$	Na^+	376.1482	2.59%
A: 3 B: 2	5	$C_{15}H_{24}N_2O_9$	K^+		
A: 4 B: 1	5	$C_{15}H_{23}NO_{10}$	H^+		
A: 4 B: 1	5	$C_{15}H_{23}NO_{10}$	Na^+	377.1322	1.75%
A: 4 B: 1	5	$C_{15}H_{23}NO_{10}$	K^+		
A: 5 B: 0	5	$C_{15}H_{22}O_{11}$	Na^+	378.1162	0.62%
A: 5 B: 0	5	$C_{15}H_{22}O_{11}$	K^+		
A: 3 B: 3	6	$C_{18}H_{29}N_3O_{10}$	K^+	447.1853	0.28%
A: 4 B: 2	6	$C_{18}H_{28}N_2O_{11}$	H^+		
A: 4 B: 2	6	$C_{18}H_{28}N_2O_{11}$	Na^+	448.1693	2.88%
A: 4 B: 2	6	$C_{18}H_{28}N_2O_{11}$	K^+		
A: 5 B: 1	6	$C_{18}H_{27}NO_{12}$	Na^+	449.1533	1.67%
A: 5 B: 1	6	$C_{18}H_{27}NO_{12}$	H^+		
A: 6 B: 0	6	$C_{18}H_{26}O_{13}$	Na^+	450.1373	0.81%
A: 6 B: 0	6	$C_{18}H_{26}O_{13}$	K^+		
A: 4 B: 3	7	$C_{21}H_{33}N_3O_{12}$	K^+	519.2064	2.74%
A: 4 B: 3	7	$C_{21}H_{33}N_3O_{12}$	Na^+		
A: 5 B: 2	7	$C_{21}H_{32}N_2O_{13}$	Na^+		
A: 5 B: 2	7	$C_{21}H_{32}N_2O_{13}$	H^+	520.1904	2.75%
A: 5 B: 2	7	$C_{21}H_{32}N_2O_{13}$	K^+		
A: 6 B: 1	7	$C_{21}H_{31}NO_{14}$	Na^+		
A: 6 B: 1	7	$C_{21}H_{31}NO_{14}$	H^+	521.1745	1.72%
A: 6 B: 1	7	$C_{21}H_{31}NO_{14}$	K^+		
A: 7 B: 0	7	$C_{21}H_{30}O_{15}$	Na^+		
A: 7 B: 0	7	$C_{21}H_{30}O_{15}$	K^+	522.1585	0.91%
A: 7 B: 0	7	$C_{21}H_{30}O_{15}$	H^+		
A: 5 B: 3	8	$C_{24}H_{37}N_3O_{14}$	K^+		
A: 5 B: 3	8	$C_{24}H_{37}N_3O_{14}$	H^+	591.2276	2.76%
A: 5 B: 3	8	$C_{24}H_{37}N_3O_{14}$	Na^+		

Composition				Monoisotopic mass	Quantity
A: 6 B: 2	8	$C_{24}H_{36}N_2O_{15}$	Na^+	592.2116	2.14%
A: 6 B: 2	8	$C_{24}H_{36}N_2O_{15}$	K^+		
A: 7 B: 1	8	$C_{24}H_{35}NO_{16}$	Na^+	593.1956	1.57%
A: 7 B: 1	8	$C_{24}H_{35}NO_{16}$	K^+		
A: 7 B: 1	8	$C_{24}H_{35}NO_{16}$	H^+		
A: 8 B: 0	8	$C_{24}H_{34}O_{17}$	Na^+	594.1796	0.63%
A: 5 B: 4	9	$C_{27}H_{42}N_4O_{15}$	K^+	662.2647	0.25%
A: 6 B: 3	9	$C_{27}H_{41}N_3O_{16}$	H^+	663.2487	1.96%
A: 6 B: 3	9	$C_{27}H_{41}N_3O_{16}$	Na^+		
A: 7 B: 2	9	$C_{27}H_{40}N_2O_{17}$	Na^+	664.2327	1.83%
A: 7 B: 2	9	$C_{27}H_{40}N_2O_{17}$	K^+		
A: 7 B: 2	9	$C_{27}H_{40}N_2O_{17}$	H^+		
A: 8 B: 1	9	$C_{27}H_{39}NO_{18}$	Na^+	665.2167	1.20%
A: 8 B: 1	9	$C_{27}H_{39}NO_{18}$	K^+		
A: 8 B: 1	9	$C_{27}H_{39}NO_{18}$	H^+		
A: 9 B: 0	9	$C_{27}H_{38}O_{19}$	Na^+	666.2007	0.63%
A: 9 B: 0	9	$C_{27}H_{38}O_{19}$	K^+		
A: 6 B: 4	10	$C_{30}H_{46}N_4O_{17}$	K^+	734.2858	1.42%
A: 6 B: 4	10	$C_{30}H_{46}N_4O_{17}$	Na^+		
A: 7 B: 3	10	$C_{30}H_{45}N_3O_{18}$	K^+	735.2698	1.50%
A: 7 B: 3	10	$C_{30}H_{45}N_3O_{18}$	Na^+		
A: 8 B: 2	10	$C_{30}H_{44}N_2O_{19}$	Na^+	736.2538	1.20%
A: 8 B: 2	10	$C_{30}H_{44}N_2O_{19}$	K^+		
A: 9 B: 1	10	$C_{30}H_{43}NO_{20}$	Na^+	737.2378	0.81%
A: 9 B: 1	10	$C_{30}H_{43}NO_{20}$	K^+		
A: 10 B: 0	10	$C_{30}H_{42}O_{21}$	Na^+	738.2219	0.57%
A: 10 B: 0	10	$C_{30}H_{42}O_{21}$	K^+		
A: 7 B: 4	11	$C_{33}H_{50}N_4O_{19}$	Na^+	806.3069	0.83%
A: 8 B: 3	11	$C_{33}H_{49}N_3O_{20}$	K^+	807.2909	1.04%
A: 8 B: 3	11	$C_{33}H_{49}N_3O_{20}$	Na^+		
A: 9 B: 2	11	$C_{33}H_{48}N_2O_{21}$	Na^+	808.2750	0.67%
A: 10 B: 1	11	$C_{33}H_{47}NO_{22}$	Na^+	809.2590	0.61%
A: 10 B: 1	11	$C_{33}H_{47}NO_{22}$	K^+		
A: 11 B: 0	11	$C_{33}H_{46}O_{23}$	Na^+	810.2430	0.28%
A: 7 B: 5	12	$C_{36}H_{55}N_5O_{20}$	Na^+	877.3440	0.47%
A: 8 B: 4	12	$C_{36}H_{54}N_4O_{21}$	Na^+	878.3281	0.59%

Composition			Monoisotopic mass	Quantity
A: 9 B: 3	12	$C_{36}H_{53}N_3O_{22}$ Na^+	879.3121	0.56%
A: 10 B: 2	12	$C_{36}H_{52}N_2O_{23}$ Na^+	880.2961	0.44%
A: 11 B: 1	12	$C_{36}H_{51}NO_{24}$ Na^+	881.2801	0.29%
A: 8 B: 5	13	$C_{39}H_{59}N_5O_{22}$ Na^+	949.3652	0.34%
A: 9 B: 4	13	$C_{39}H_{58}N_4O_{23}$ Na^+	950.3492	0.39%
A: 10 B: 3	13	$C_{39}H_{57}N_3O_{24}$ Na^+	951.3332	0.37%
A: 11 B: 2	13	$C_{39}H_{56}N_2O_{25}$ Na^+	952.3172	0.28%
A: 12 B: 1	13	$C_{39}H_{55}NO_{26}$ Na^+	953.3012	0.20%
A: 13 B: 0	13	$C_{39}H_{54}O_{27}$ Na^+	954.2852	0.15%
A: 9 B: 5	14	$C_{42}H_{63}N_5O_{24}$ Na^+	1021.3863	0.26%
A: 10 B: 4	14	$C_{42}H_{62}N_4O_{25}$ Na^+	1022.3703	0.28%
A: 11 B: 3	14	$C_{42}H_{61}N_3O_{26}$ Na^+	1023.3543	0.26%
A: 12 B: 2	14	$C_{42}H_{60}N_2O_{27}$ Na^+	1024.3383	0.21%
A: 13 B: 1	14	$C_{42}H_{59}NO_{28}$ Na^+	1025.3224	0.16%
A: 11 B: 4	15	$C_{45}H_{66}N_4O_{27}$ Na^+	1094.3914	0.19%
A: 12 B: 3	15	$C_{45}H_{65}N_3O_{28}$ Na^+	1095.3755	0.18%
A: 12 B: 4	16	$C_{48}H_{70}N_4O_{29}$ Na^+	1166.4126	0.14%

Analytical report

Page under construction...

aA MALDI, 4cyc, no dust ctrl

Analysis parameters

Ionizations

MF	Monoisotopic mass	m/z mass
H^+	1.00783	1.00728
Na^+	22.98977	22.98922
K^+	38.96371	38.96316

End groups

α	ω	Color
H	OH	

Monomers

	Description	mf	Monoisotopic mass	min	max
A	Lactic acid	$C_3H_4O_2$	72.02113	0	100
B	Alanine	C_3H_5NO	71.03711	0	100

Experimental spectrum

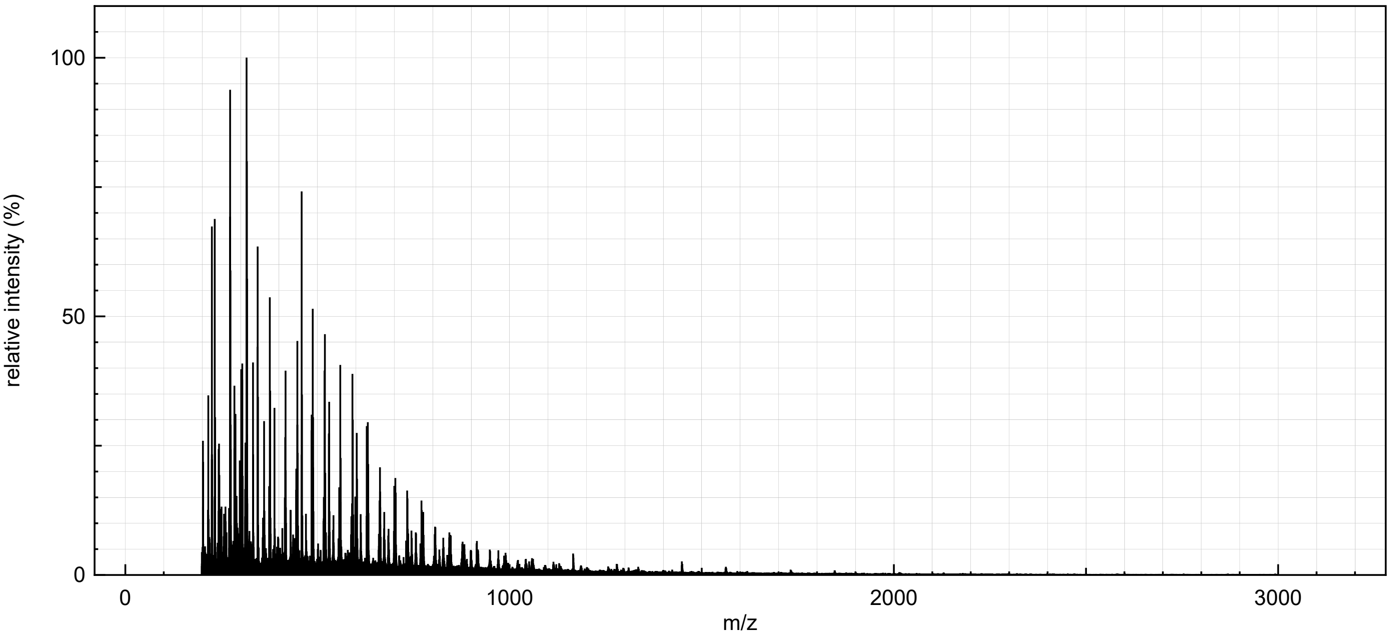

Reconstructed spectrum

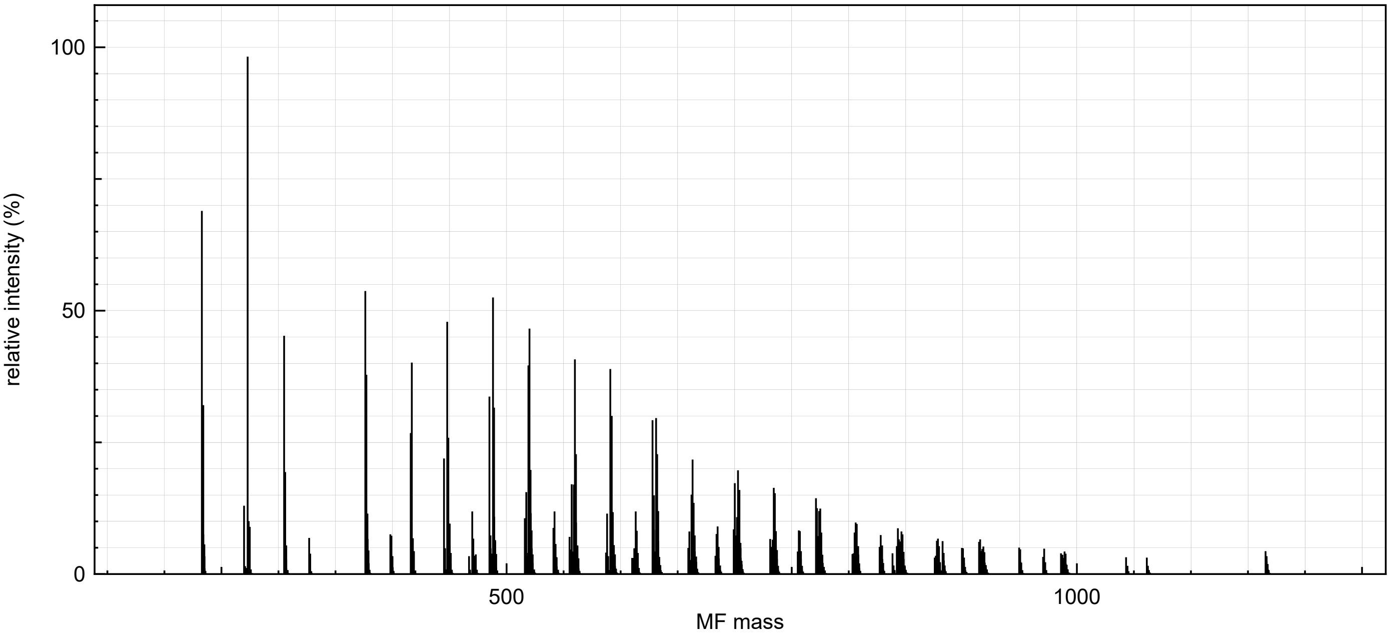

Error (ppm) versus m/z

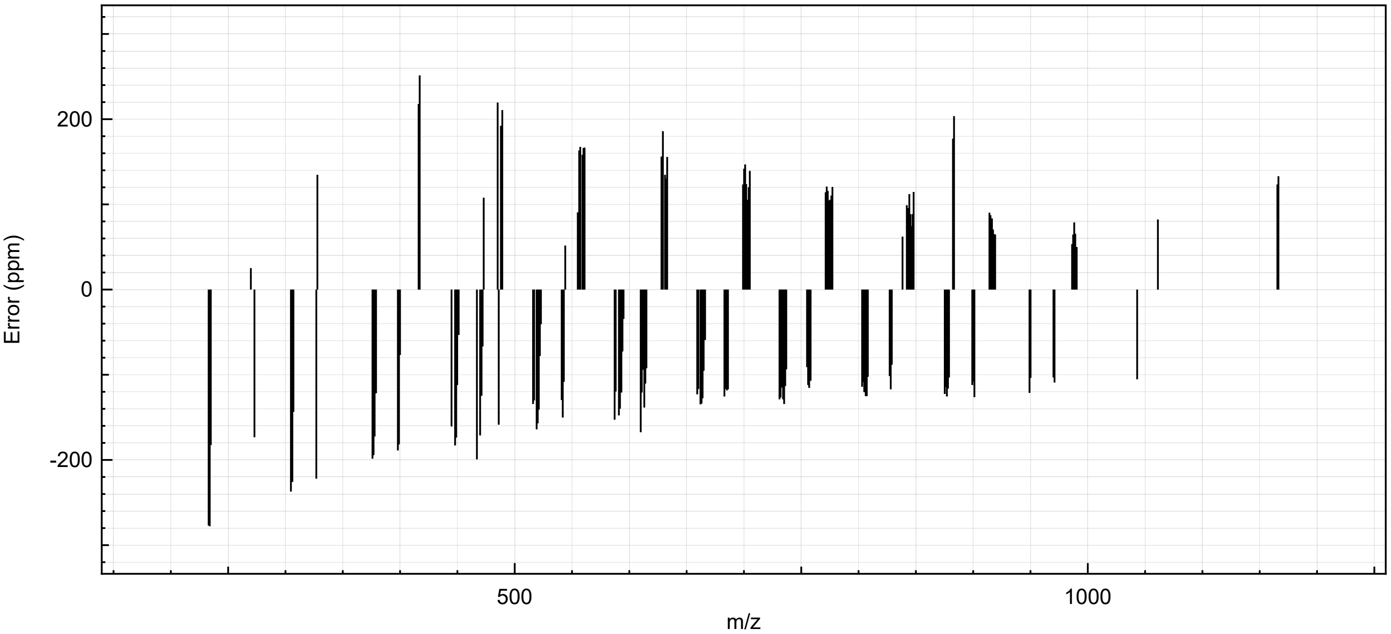

Analysis result

Percent of peaks covered: 48.27%
Number of assigned peaks: 150

Polymers [m/z]

Composition	Nb units	MF	Ioniz.	em [m/z]	Charge	Similarity	Quantity
A: 0 B: 3	3	$C_9H_{17}N_3O_4$	K^+	270.08506	1	50.98%	0.30%
A: 1 B: 2	3	$C_9H_{16}N_2O_5$	H^+	233.11320	1	65.63%	0.76%
A: 2 B: 1	3	$C_9H_{15}NO_6$	H^+	234.09721	1	80.93%	0.39%
A: 3 B: 0	3	$C_9H_{14}O_7$	K^+	273.03711	1	74.55%	1.94%
A: 3 B: 0	3	$C_9H_{14}O_7$	H^+	235.08123	1	57.80%	0.16%
A: 2 B: 2	4	$C_{12}H_{20}N_2O_7$	H^+	305.13433	1	70.38%	0.70%
A: 2 B: 2	4	$C_{12}H_{20}N_2O_7$	Na^+	327.11627	1	61.17%	0.24%
A: 3 B: 1	4	$C_{12}H_{19}NO_8$	H^+	306.11834	1	69.59%	0.39%
A: 3 B: 1	4	$C_{12}H_{19}NO_8$	Na^+	328.10029	1	50.69%	0.19%
A: 4 B: 0	4	$C_{12}H_{18}O_9$	H^+	307.10236	1	60.97%	0.21%
A: 2 B: 3	5	$C_{15}H_{25}N_3O_8$	H^+	376.17144	1	59.15%	0.82%
A: 2 B: 3	5	$C_{15}H_{25}N_3O_8$	Na^+	398.15339	1	52.53%	0.25%
A: 3 B: 2	5	$C_{15}H_{24}N_2O_9$	H^+	377.15546	1	79.09%	0.55%
A: 3 B: 2	5	$C_{15}H_{24}N_2O_9$	Na^+	399.13740	1	62.53%	0.21%
A: 4 B: 1	5	$C_{15}H_{23}NO_{10}$	H^+	378.13947	1	68.01%	0.26%
A: 4 B: 1	5	$C_{15}H_{23}NO_{10}$	Na^+	400.12142	1	51.44%	0.19%
A: 4 B: 1	5	$C_{15}H_{23}NO_{10}$	K^+	416.09535	1	54.65%	0.66%
A: 5 B: 0	5	$C_{15}H_{22}O_{11}$	H^+	379.12349	1	56.69%	0.18%
A: 5 B: 0	5	$C_{15}H_{22}O_{11}$	K^+	417.07937	1	80.83%	0.51%
A: 0 B: 6	6	$C_{18}H_{32}N_6O_7$	H^+	445.24052	1	55.16%	0.58%
A: 0 B: 6	6	$C_{18}H_{32}N_6O_7$	Na^+	467.22247	1	52.44%	0.23%
A: 2 B: 4	6	$C_{18}H_{30}N_4O_9$	K^+	485.16444	1	79.27%	0.62%
A: 3 B: 3	6	$C_{18}H_{29}N_3O_{10}$	H^+	448.19257	1	71.54%	0.72%
A: 3 B: 3	6	$C_{18}H_{29}N_3O_{10}$	Na^+	470.17451	1	59.89%	0.31%
A: 3 B: 3	6	$C_{18}H_{29}N_3O_{10}$	K^+	486.14845	1	51.67%	0.65%
A: 4 B: 2	6	$C_{18}H_{28}N_2O_{11}$	H^+	449.17659	1	75.90%	0.43%
A: 4 B: 2	6	$C_{18}H_{28}N_2O_{11}$	Na^+	471.15853	1	60.55%	0.27%
A: 5 B: 1	6	$C_{18}H_{27}NO_{12}$	H^+	450.16060	1	64.36%	0.26%
A: 5 B: 1	6	$C_{18}H_{27}NO_{12}$	Na^+	472.14255	1	57.56%	0.22%

Composition	Nb units	MF	Ioniz.	em [m/z]	Charge	Similarity	Quantity
A: 5 B: 1	6	$C_{18}H_{27}NO_{12}$	K^+	488.11648	1	72.36%	0.76%
A: 6 B: 0	6	$C_{18}H_{26}O_{13}$	H^+	451.14462	1	52.99%	0.24%
A: 6 B: 0	6	$C_{18}H_{26}O_{13}$	Na^+	473.12656	1	53.20%	0.21%
A: 6 B: 0	6	$C_{18}H_{26}O_{13}$	K^+	489.10050	1	81.64%	0.47%
A: 0 B: 7	7	$C_{21}H_{37}N_7O_8$	H^+	516.27764	1	53.74%	0.44%
A: 1 B: 6	7	$C_{21}H_{36}N_6O_9$	H^+	517.26165	1	55.27%	0.56%
A: 1 B: 6	7	$C_{21}H_{36}N_6O_9$	K^+	555.21753	1	57.31%	0.37%
A: 2 B: 5	7	$C_{21}H_{35}N_5O_{10}$	K^+	556.20155	1	50.73%	0.37%
A: 3 B: 4	7	$C_{21}H_{34}N_4O_{11}$	H^+	519.22968	1	58.63%	0.72%
A: 3 B: 4	7	$C_{21}H_{34}N_4O_{11}$	Na^+	541.21163	1	59.37%	0.32%
A: 3 B: 4	7	$C_{21}H_{34}N_4O_{11}$	K^+	557.18557	1	63.29%	0.47%
A: 4 B: 3	7	$C_{21}H_{33}N_3O_{12}$	H^+	520.21370	1	75.23%	0.58%
A: 4 B: 3	7	$C_{21}H_{33}N_3O_{12}$	Na^+	542.19564	1	67.95%	0.27%
A: 5 B: 2	7	$C_{21}H_{32}N_2O_{13}$	H^+	521.19772	1	71.05%	0.35%
A: 5 B: 2	7	$C_{21}H_{32}N_2O_{13}$	Na^+	543.17966	1	63.53%	0.21%
A: 5 B: 2	7	$C_{21}H_{32}N_2O_{13}$	K^+	559.15360	1	55.95%	0.64%
A: 6 B: 1	7	$C_{21}H_{31}NO_{14}$	H^+	522.18173	1	66.15%	0.25%
A: 6 B: 1	7	$C_{21}H_{31}NO_{14}$	Na^+	544.16368	1	55.40%	0.19%
A: 6 B: 1	7	$C_{21}H_{31}NO_{14}$	K^+	560.13761	1	79.48%	0.53%
A: 7 B: 0	7	$C_{21}H_{30}O_{15}$	H^+	523.16575	1	56.39%	0.21%
A: 7 B: 0	7	$C_{21}H_{30}O_{15}$	K^+	561.12163	1	78.52%	0.32%
A: 0 B: 8	8	$C_{24}H_{42}N_8O_9$	H^+	587.31475	1	52.84%	0.35%
A: 1 B: 7	8	$C_{24}H_{41}N_7O_{10}$	H^+	588.29877	1	63.53%	0.39%
A: 1 B: 7	8	$C_{24}H_{41}N_7O_{10}$	Na^+	610.28071	1	57.74%	0.21%
A: 2 B: 6	8	$C_{24}H_{40}N_6O_{11}$	Na^+	611.26473	1	50.74%	0.27%
A: 3 B: 5	8	$C_{24}H_{39}N_5O_{12}$	Na^+	612.24874	1	51.63%	0.31%
A: 3 B: 5	8	$C_{24}H_{39}N_5O_{12}$	K^+	628.22268	1	73.77%	0.52%
A: 4 B: 4	8	$C_{24}H_{38}N_4O_{13}$	H^+	591.25081	1	70.15%	0.58%
A: 4 B: 4	8	$C_{24}H_{38}N_4O_{13}$	Na^+	613.23276	1	66.25%	0.31%
A: 4 B: 4	8	$C_{24}H_{38}N_4O_{13}$	K^+	629.20670	1	64.20%	0.52%
A: 5 B: 3	8	$C_{24}H_{37}N_3O_{14}$	H^+	592.23483	1	76.83%	0.42%
A: 5 B: 3	8	$C_{24}H_{37}N_3O_{14}$	Na^+	614.21677	1	68.71%	0.25%
A: 6 B: 2	8	$C_{24}H_{36}N_2O_{15}$	H^+	593.21884	1	68.43%	0.28%
A: 6 B: 2	8	$C_{24}H_{36}N_2O_{15}$	Na^+	615.20079	1	64.16%	0.20%

Composition	Nb units	MF	Ioniz.	em [m/z]	Charge	Similarity	Quantity
A: 6 B: 2	8	$C_{24}H_{36}N_2O_{15}$	K^+	631.17473	1	70.59%	0.55%
A: 7 B: 1	8	$C_{24}H_{35}NO_{16}$	H^+	594.20286	1	61.69%	0.24%
A: 7 B: 1	8	$C_{24}H_{35}NO_{16}$	K^+	632.15874	1	79.21%	0.40%
A: 8 B: 0	8	$C_{24}H_{34}O_{17}$	H^+	595.18688	1	53.65%	0.24%
A: 8 B: 0	8	$C_{24}H_{34}O_{17}$	K^+	633.14276	1	71.41%	0.27%
A: 1 B: 8	9	$C_{27}H_{46}N_8O_{11}$	H^+	659.33588	1	60.22%	0.28%
A: 2 B: 7	9	$C_{27}H_{45}N_7O_{12}$	H^+	660.31990	1	58.86%	0.34%
A: 3 B: 6	9	$C_{27}H_{44}N_6O_{13}$	Na^+	683.28586	1	51.21%	0.28%
A: 3 B: 6	9	$C_{27}H_{44}N_6O_{13}$	K^+	699.25979	1	57.82%	0.39%
A: 4 B: 5	9	$C_{27}H_{43}N_5O_{14}$	H^+	662.28793	1	59.07%	0.50%
A: 4 B: 5	9	$C_{27}H_{43}N_5O_{14}$	Na^+	684.26987	1	62.01%	0.29%
A: 4 B: 5	9	$C_{27}H_{43}N_5O_{14}$	K^+	700.24381	1	72.23%	0.43%
A: 5 B: 4	9	$C_{27}H_{42}N_4O_{15}$	H^+	663.27194	1	73.46%	0.44%
A: 5 B: 4	9	$C_{27}H_{42}N_4O_{15}$	Na^+	685.25389	1	72.27%	0.25%
A: 5 B: 4	9	$C_{27}H_{42}N_4O_{15}$	K^+	701.22782	1	59.51%	0.43%
A: 6 B: 3	9	$C_{27}H_{41}N_3O_{16}$	H^+	664.25596	1	74.42%	0.31%
A: 6 B: 3	9	$C_{27}H_{41}N_3O_{16}$	Na^+	686.23790	1	70.58%	0.20%
A: 6 B: 3	9	$C_{27}H_{41}N_3O_{16}$	K^+	702.21184	1	60.11%	0.46%
A: 7 B: 2	9	$C_{27}H_{40}N_2O_{17}$	H^+	665.23997	1	70.17%	0.24%
A: 7 B: 2	9	$C_{27}H_{40}N_2O_{17}$	K^+	703.19586	1	75.85%	0.41%
A: 8 B: 1	9	$C_{27}H_{39}NO_{18}$	H^+	666.22399	1	60.75%	0.21%
A: 8 B: 1	9	$C_{27}H_{39}NO_{18}$	K^+	704.17987	1	78.35%	0.30%
A: 9 B: 0	9	$C_{27}H_{38}O_{19}$	K^+	705.16389	1	75.87%	0.21%
A: 2 B: 8	10	$C_{30}H_{50}N_8O_{13}$	H^+	731.35701	1	66.36%	0.24%
A: 3 B: 7	10	$C_{30}H_{49}N_7O_{14}$	H^+	732.34103	1	56.12%	0.30%
A: 4 B: 6	10	$C_{30}H_{48}N_6O_{15}$	H^+	733.32504	1	53.35%	0.37%
A: 4 B: 6	10	$C_{30}H_{48}N_6O_{15}$	Na^+	755.30699	1	55.99%	0.27%
A: 4 B: 6	10	$C_{30}H_{48}N_6O_{15}$	K^+	771.28092	1	70.55%	0.37%
A: 5 B: 5	10	$C_{30}H_{47}N_5O_{16}$	H^+	734.30906	1	67.97%	0.39%
A: 5 B: 5	10	$C_{30}H_{47}N_5O_{16}$	Na^+	756.29100	1	67.12%	0.27%
A: 5 B: 5	10	$C_{30}H_{47}N_5O_{16}$	K^+	772.26494	1	70.44%	0.37%
A: 6 B: 4	10	$C_{30}H_{46}N_4O_{17}$	H^+	735.29307	1	76.04%	0.32%
A: 6 B: 4	10	$C_{30}H_{46}N_4O_{17}$	Na^+	757.27502	1	74.85%	0.23%
A: 6 B: 4	10	$C_{30}H_{46}N_4O_{17}$	K^+	773.24895	1	62.56%	0.37%

Composition	Nb units	MF	Ioniz.	em [m/z]	Charge	Similarity	Quantity
A: 7 B: 3	10	$C_{30}H_{45}N_3O_{18}$	H^+	736.27709	1	71.46%	0.24%
A: 7 B: 3	10	$C_{30}H_{45}N_3O_{18}$	Na^+	758.25903	1	71.84%	0.18%
A: 7 B: 3	10	$C_{30}H_{45}N_3O_{18}$	K^+	774.23297	1	70.34%	0.36%
A: 8 B: 2	10	$C_{30}H_{44}N_2O_{19}$	H^+	737.26110	1	66.31%	0.21%
A: 8 B: 2	10	$C_{30}H_{44}N_2O_{19}$	K^+	775.21699	1	78.87%	0.29%
A: 9 B: 1	10	$C_{30}H_{43}NO_{20}$	K^+	776.20100	1	79.04%	0.21%
A: 10 B: 0	10	$C_{30}H_{42}O_{21}$	K^+	777.18502	1	73.08%	0.16%
A: 0 B: 11	11	$C_{33}H_{57}N_{11}O_{12}$	K^+	838.38197	1	76.04%	0.18%
A: 3 B: 8	11	$C_{33}H_{54}N_8O_{15}$	H^+	803.37814	1	63.23%	0.21%
A: 4 B: 7	11	$C_{33}H_{53}N_7O_{16}$	H^+	804.36216	1	57.86%	0.25%
A: 4 B: 7	11	$C_{33}H_{53}N_7O_{16}$	K^+	842.31804	1	62.04%	0.27%
A: 5 B: 6	11	$C_{33}H_{52}N_6O_{17}$	H^+	805.34617	1	63.01%	0.29%
A: 5 B: 6	11	$C_{33}H_{52}N_6O_{17}$	Na^+	827.32811	1	64.59%	0.24%
A: 5 B: 6	11	$C_{33}H_{52}N_6O_{17}$	K^+	843.30205	1	74.52%	0.29%
A: 6 B: 5	11	$C_{33}H_{51}N_5O_{18}$	H^+	806.33019	1	71.25%	0.28%
A: 6 B: 5	11	$C_{33}H_{51}N_5O_{18}$	Na^+	828.31213	1	73.48%	0.22%
A: 6 B: 5	11	$C_{33}H_{51}N_5O_{18}$	K^+	844.28607	1	69.44%	0.29%
A: 7 B: 4	11	$C_{33}H_{50}N_4O_{19}$	H^+	807.31420	1	76.18%	0.23%
A: 7 B: 4	11	$C_{33}H_{50}N_4O_{19}$	Na^+	829.29615	1	76.17%	0.18%
A: 7 B: 4	11	$C_{33}H_{50}N_4O_{19}$	K^+	845.27008	1	69.75%	0.28%
A: 8 B: 3	11	$C_{33}H_{49}N_3O_{20}$	H^+	808.29822	1	72.92%	0.19%
A: 8 B: 3	11	$C_{33}H_{49}N_3O_{20}$	K^+	846.25410	1	75.50%	0.26%
A: 9 B: 2	11	$C_{33}H_{48}N_2O_{21}$	K^+	847.23811	1	79.47%	0.21%
A: 10 B: 1	11	$C_{33}H_{47}NO_{22}$	K^+	848.22213	1	77.82%	0.17%
A: 4 B: 8	12	$C_{36}H_{58}N_8O_{17}$	H^+	875.39927	1	63.91%	0.17%
A: 5 B: 7	12	$C_{36}H_{57}N_7O_{18}$	H^+	876.38328	1	61.29%	0.21%
A: 5 B: 7	12	$C_{36}H_{57}N_7O_{18}$	K^+	914.33917	1	73.17%	0.23%
A: 6 B: 6	12	$C_{36}H_{56}N_6O_{19}$	H^+	877.36730	1	68.99%	0.23%
A: 6 B: 6	12	$C_{36}H_{56}N_6O_{19}$	Na^+	899.34924	1	72.98%	0.21%
A: 6 B: 6	12	$C_{36}H_{56}N_6O_{19}$	K^+	915.32318	1	74.52%	0.23%
A: 7 B: 5	12	$C_{36}H_{55}N_5O_{20}$	H^+	878.35132	1	75.69%	0.21%
A: 7 B: 5	12	$C_{36}H_{55}N_5O_{20}$	Na^+	900.33326	1	78.27%	0.18%
A: 7 B: 5	12	$C_{36}H_{55}N_5O_{20}$	K^+	916.30720	1	71.81%	0.22%
A: 8 B: 4	12	$C_{36}H_{54}N_4O_{21}$	H^+	879.33533	1	77.35%	0.17%

Composition	Nb units	MF	Ioniz.	em [m/z]	Charge	Similarity	Quantity
A: 8 B: 4	12	$C_{36}H_{54}N_4O_{21}$	Na^+	901.31728	1	77.60%	0.14%
A: 8 B: 4	12	$C_{36}H_{54}N_4O_{21}$	K^+	917.29121	1	74.72%	0.21%
A: 9 B: 3	12	$C_{36}H_{53}N_3O_{22}$	K^+	918.27523	1	78.66%	0.18%
A: 10 B: 2	12	$C_{36}H_{52}N_2O_{23}$	K^+	919.25924	1	81.79%	0.15%
A: 11 B: 1	12	$C_{36}H_{51}NO_{24}$	H^+	882.28738	1	78.16%	0.21%
A: 12 B: 0	12	$C_{36}H_{50}O_{25}$	H^+	883.27139	1	77.22%	0.16%
A: 6 B: 7	13	$C_{39}H_{61}N_7O_{20}$	Na^+	970.38636	1	71.55%	0.17%
A: 6 B: 7	13	$C_{39}H_{61}N_7O_{20}$	K^+	986.36030	1	77.38%	0.17%
A: 7 B: 6	13	$C_{39}H_{60}N_6O_{21}$	H^+	949.38843	1	74.32%	0.17%
A: 7 B: 6	13	$C_{39}H_{60}N_6O_{21}$	Na^+	971.37037	1	78.16%	0.16%
A: 7 B: 6	13	$C_{39}H_{60}N_6O_{21}$	K^+	987.34431	1	76.73%	0.17%
A: 8 B: 5	13	$C_{39}H_{59}N_5O_{22}$	H^+	950.37245	1	78.87%	0.15%
A: 8 B: 5	13	$C_{39}H_{59}N_5O_{22}$	K^+	988.32833	1	73.89%	0.17%
A: 9 B: 4	13	$C_{39}H_{58}N_4O_{23}$	K^+	989.31234	1	78.38%	0.16%
A: 10 B: 3	13	$C_{39}H_{57}N_3O_{24}$	K^+	990.29636	1	81.57%	0.14%
A: 8 B: 6	14	$C_{42}H_{64}N_6O_{23}$	Na^+	1043.39150	1	80.61%	0.13%
A: 10 B: 4	14	$C_{42}H_{62}N_4O_{25}$	K^+	1061.33347	1	84.76%	0.13%
A: 10 B: 6	16	$C_{48}H_{72}N_6O_{27}$	H^+	1165.45182	1	87.98%	0.15%
A: 11 B: 5	16	$C_{48}H_{71}N_5O_{28}$	H^+	1166.43583	1	88.81%	0.12%

Polymers grouped by monoisotopic mass

Composition				Monoisotopic mass	Quantity
A: 0 B: 3	3	$C_9H_{17}N_3O_4$	K^+	231.1219	0.30%
A: 1 B: 2	3	$C_9H_{16}N_2O_5$	H^+	232.1059	0.76%
A: 2 B: 1	3	$C_9H_{15}NO_6$	H^+	233.0899	0.39%
A: 3 B: 0<br>A: 3 B: 0	3<br>3	$C_9H_{14}O_7$<br>$C_9H_{14}O_7$	K^+<br>H^+	234.0740	2.10%
A: 2 B: 2<br>A: 2 B: 2	4<br>4	$C_{12}H_{20}N_2O_7$<br>$C_{12}H_{20}N_2O_7$	H^+<br>Na^+	304.1271	0.94%
A: 3 B: 1<br>A: 3 B: 1	4<br>4	$C_{12}H_{19}NO_8$<br>$C_{12}H_{19}NO_8$	H^+<br>Na^+	305.1111	0.57%
A: 4 B: 0	4	$C_{12}H_{18}O_9$	H^+	306.0951	0.21%
A: 2 B: 3<br>A: 2 B: 3	5<br>5	$C_{15}H_{25}N_3O_8$<br>$C_{15}H_{25}N_3O_8$	H^+<br>Na^+	375.1642	1.06%
A: 3 B: 2	5	$C_{15}H_{24}N_2O_9$	H^+	376.1482	0.76%

		Composition		Monoisotopic mass	Quantity
A: 3 B: 2	5	$C_{15}H_{24}N_2O_9$	Na^+		
A: 4 B: 1	5	$C_{15}H_{23}NO_{10}$	H^+	377.1322	1.11%
A: 4 B: 1	5	$C_{15}H_{23}NO_{10}$	K^+		
A: 4 B: 1	5	$C_{15}H_{23}NO_{10}$	Na^+		
A: 5 B: 0	5	$C_{15}H_{22}O_{11}$	K^+	378.1162	0.69%
A: 5 B: 0	5	$C_{15}H_{22}O_{11}$	H^+		
A: 0 B: 6	6	$C_{18}H_{32}N_6O_7$	H^+	444.2332	0.81%
A: 0 B: 6	6	$C_{18}H_{32}N_6O_7$	Na^+		
A: 2 B: 4	6	$C_{18}H_{30}N_4O_9$	K^+	446.2013	0.62%
A: 3 B: 3	6	$C_{18}H_{29}N_3O_{10}$	H^+	447.1853	1.68%
A: 3 B: 3	6	$C_{18}H_{29}N_3O_{10}$	Na^+		
A: 3 B: 3	6	$C_{18}H_{29}N_3O_{10}$	K^+		
A: 4 B: 2	6	$C_{18}H_{28}N_2O_{11}$	H^+	448.1693	0.70%
A: 4 B: 2	6	$C_{18}H_{28}N_2O_{11}$	Na^+		
A: 5 B: 1	6	$C_{18}H_{27}NO_{12}$	K^+	449.1533	1.23%
A: 5 B: 1	6	$C_{18}H_{27}NO_{12}$	H^+		
A: 5 B: 1	6	$C_{18}H_{27}NO_{12}$	Na^+		
A: 6 B: 0	6	$C_{18}H_{26}O_{13}$	K^+	450.1373	0.92%
A: 6 B: 0	6	$C_{18}H_{26}O_{13}$	Na^+		
A: 6 B: 0	6	$C_{18}H_{26}O_{13}$	H^+		
A: 0 B: 7	7	$C_{21}H_{37}N_7O_8$	H^+	515.2704	0.44%
A: 1 B: 6	7	$C_{21}H_{36}N_6O_9$	K^+	516.2544	0.94%
A: 1 B: 6	7	$C_{21}H_{36}N_6O_9$	H^+		
A: 2 B: 5	7	$C_{21}H_{35}N_5O_{10}$	K^+	517.2384	0.37%
A: 3 B: 4	7	$C_{21}H_{34}N_4O_{11}$	K^+	518.2224	1.52%
A: 3 B: 4	7	$C_{21}H_{34}N_4O_{11}$	Na^+		
A: 3 B: 4	7	$C_{21}H_{34}N_4O_{11}$	H^+		
A: 4 B: 3	7	$C_{21}H_{33}N_3O_{12}$	H^+	519.2064	0.85%
A: 4 B: 3	7	$C_{21}H_{33}N_3O_{12}$	Na^+		
A: 5 B: 2	7	$C_{21}H_{32}N_2O_{13}$	H^+	520.1904	1.21%
A: 5 B: 2	7	$C_{21}H_{32}N_2O_{13}$	Na^+		
A: 5 B: 2	7	$C_{21}H_{32}N_2O_{13}$	K^+		
A: 6 B: 1	7	$C_{21}H_{31}NO_{14}$	K^+	521.1745	0.98%
A: 6 B: 1	7	$C_{21}H_{31}NO_{14}$	H^+		
A: 6 B: 1	7	$C_{21}H_{31}NO_{14}$	Na^+		
A: 7 B: 0	7	$C_{21}H_{30}O_{15}$	K^+	522.1585	0.53%
A: 7 B: 0	7	$C_{21}H_{30}O_{15}$	H^+		

		Composition		Monoisotopic mass	Quantity
A: 0 B: 8	8	$C_{24}H_{42}N_8O_9$	H^+	586.3075	0.35%
A: 1 B: 7	8	$C_{24}H_{41}N_7O_{10}$	H^+	587.2915	0.60%
A: 1 B: 7	8	$C_{24}H_{41}N_7O_{10}$	Na^+		
A: 2 B: 6	8	$C_{24}H_{40}N_6O_{11}$	Na^+	588.2755	0.27%
A: 3 B: 5	8	$C_{24}H_{39}N_5O_{12}$	K^+	589.2595	0.83%
A: 3 B: 5	8	$C_{24}H_{39}N_5O_{12}$	Na^+		
A: 4 B: 4	8	$C_{24}H_{38}N_4O_{13}$	H^+	590.2435	1.41%
A: 4 B: 4	8	$C_{24}H_{38}N_4O_{13}$	Na^+		
A: 4 B: 4	8	$C_{24}H_{38}N_4O_{13}$	K^+		
A: 5 B: 3	8	$C_{24}H_{37}N_3O_{14}$	H^+	591.2276	0.66%
A: 5 B: 3	8	$C_{24}H_{37}N_3O_{14}$	Na^+		
A: 6 B: 2	8	$C_{24}H_{36}N_2O_{15}$	K^+	592.2116	1.02%
A: 6 B: 2	8	$C_{24}H_{36}N_2O_{15}$	H^+		
A: 6 B: 2	8	$C_{24}H_{36}N_2O_{15}$	Na^+		
A: 7 B: 1	8	$C_{24}H_{35}NO_{16}$	K^+	593.1956	0.64%
A: 7 B: 1	8	$C_{24}H_{35}NO_{16}$	H^+		
A: 8 B: 0	8	$C_{24}H_{34}O_{17}$	K^+	594.1796	0.51%
A: 8 B: 0	8	$C_{24}H_{34}O_{17}$	H^+		
A: 1 B: 8	9	$C_{27}H_{46}N_8O_{11}$	H^+	658.3286	0.28%
A: 2 B: 7	9	$C_{27}H_{45}N_7O_{12}$	H^+	659.3126	0.34%
A: 3 B: 6	9	$C_{27}H_{44}N_6O_{13}$	K^+	660.2966	0.67%
A: 3 B: 6	9	$C_{27}H_{44}N_6O_{13}$	Na^+		
A: 4 B: 5	9	$C_{27}H_{43}N_5O_{14}$	K^+	661.2807	1.22%
A: 4 B: 5	9	$C_{27}H_{43}N_5O_{14}$	Na^+		
A: 4 B: 5	9	$C_{27}H_{43}N_5O_{14}$	H^+		
A: 5 B: 4	9	$C_{27}H_{42}N_4O_{15}$	H^+	662.2647	1.13%
A: 5 B: 4	9	$C_{27}H_{42}N_4O_{15}$	Na^+		
A: 5 B: 4	9	$C_{27}H_{42}N_4O_{15}$	K^+		
A: 6 B: 3	9	$C_{27}H_{41}N_3O_{16}$	H^+	663.2487	0.98%
A: 6 B: 3	9	$C_{27}H_{41}N_3O_{16}$	Na^+		
A: 6 B: 3	9	$C_{27}H_{41}N_3O_{16}$	K^+		
A: 7 B: 2	9	$C_{27}H_{40}N_2O_{17}$	K^+	664.2327	0.65%
A: 7 B: 2	9	$C_{27}H_{40}N_2O_{17}$	H^+		
A: 8 B: 1	9	$C_{27}H_{39}NO_{18}$	K^+	665.2167	0.51%
A: 8 B: 1	9	$C_{27}H_{39}NO_{18}$	H^+		
A: 9 B: 0	9	$C_{27}H_{38}O_{19}$	K^+	666.2007	0.21%

		Composition	Monoisotopic mass	Quantity
A: 2 B: 8	10	$C_{30}H_{50}N_8O_{13}$ H^+	730.3497	0.24%
A: 3 B: 7	10	$C_{30}H_{49}N_7O_{14}$ H^+	731.3337	0.30%
A: 4 B: 6	10	$C_{30}H_{48}N_6O_{15}$ K^+		
A: 4 B: 6	10	$C_{30}H_{48}N_6O_{15}$ Na^+	732.3178	1.01%
A: 4 B: 6	10	$C_{30}H_{48}N_6O_{15}$ H^+		
A: 5 B: 5	10	$C_{30}H_{47}N_5O_{16}$ K^+		
A: 5 B: 5	10	$C_{30}H_{47}N_5O_{16}$ H^+	733.3018	1.03%
A: 5 B: 5	10	$C_{30}H_{47}N_5O_{16}$ Na^+		
A: 6 B: 4	10	$C_{30}H_{46}N_4O_{17}$ H^+		
A: 6 B: 4	10	$C_{30}H_{46}N_4O_{17}$ Na^+	734.2858	0.91%
A: 6 B: 4	10	$C_{30}H_{46}N_4O_{17}$ K^+		
A: 7 B: 3	10	$C_{30}H_{45}N_3O_{18}$ Na^+		
A: 7 B: 3	10	$C_{30}H_{45}N_3O_{18}$ H^+	735.2698	0.79%
A: 7 B: 3	10	$C_{30}H_{45}N_3O_{18}$ K^+		
A: 8 B: 2	10	$C_{30}H_{44}N_2O_{19}$ K^+	736.2538	0.50%
A: 8 B: 2	10	$C_{30}H_{44}N_2O_{19}$ H^+		
A: 9 B: 1	10	$C_{30}H_{43}NO_{20}$ K^+	737.2378	0.21%
A: 10 B: 0	10	$C_{30}H_{42}O_{21}$ K^+	738.2219	0.16%
A: 0 B: 11	11	$C_{33}H_{57}N_{11}O_{12}$ K^+	799.4188	0.18%
A: 3 B: 8	11	$C_{33}H_{54}N_8O_{15}$ H^+	802.3709	0.21%
A: 4 B: 7	11	$C_{33}H_{53}N_7O_{16}$ K^+	803.3549	0.52%
A: 4 B: 7	11	$C_{33}H_{53}N_7O_{16}$ H^+		
A: 5 B: 6	11	$C_{33}H_{52}N_6O_{17}$ K^+		
A: 5 B: 6	11	$C_{33}H_{52}N_6O_{17}$ Na^+	804.3389	0.82%
A: 5 B: 6	11	$C_{33}H_{52}N_6O_{17}$ H^+		
A: 6 B: 5	11	$C_{33}H_{51}N_5O_{18}$ Na^+		
A: 6 B: 5	11	$C_{33}H_{51}N_5O_{18}$ H^+	805.3229	0.79%
A: 6 B: 5	11	$C_{33}H_{51}N_5O_{18}$ K^+		
A: 7 B: 4	11	$C_{33}H_{50}N_4O_{19}$ H^+		
A: 7 B: 4	11	$C_{33}H_{50}N_4O_{19}$ Na^+	806.3069	0.70%
A: 7 B: 4	11	$C_{33}H_{50}N_4O_{19}$ K^+		
A: 8 B: 3	11	$C_{33}H_{49}N_3O_{20}$ K^+	807.2909	0.45%
A: 8 B: 3	11	$C_{33}H_{49}N_3O_{20}$ H^+		
A: 9 B: 2	11	$C_{33}H_{48}N_2O_{21}$ K^+	808.2750	0.21%
A: 10 B: 1	11	$C_{33}H_{47}NO_{22}$ K^+	809.2590	0.17%
A: 4 B: 8	12	$C_{36}H_{58}N_8O_{17}$ H^+	874.3920	0.17%

Composition			Monoisotopic mass	Quantity
A: 5 B: 7	12	$C_{36}H_{57}N_7O_{18}$ K^+	875.3760	0.43%
A: 5 B: 7	12	$C_{36}H_{57}N_7O_{18}$ H^+		
A: 6 B: 6	12	$C_{36}H_{56}N_6O_{19}$ K^+	876.3600	0.66%
A: 6 B: 6	12	$C_{36}H_{56}N_6O_{19}$ Na^+		
A: 6 B: 6	12	$C_{36}H_{56}N_6O_{19}$ H^+		
A: 7 B: 5	12	$C_{36}H_{55}N_5O_{20}$ Na^+	877.3440	0.60%
A: 7 B: 5	12	$C_{36}H_{55}N_5O_{20}$ H^+		
A: 7 B: 5	12	$C_{36}H_{55}N_5O_{20}$ K^+		
A: 8 B: 4	12	$C_{36}H_{54}N_4O_{21}$ Na^+	878.3281	0.53%
A: 8 B: 4	12	$C_{36}H_{54}N_4O_{21}$ H^+		
A: 8 B: 4	12	$C_{36}H_{54}N_4O_{21}$ K^+		
A: 9 B: 3	12	$C_{36}H_{53}N_3O_{22}$ K^+	879.3121	0.18%
A: 10 B: 2	12	$C_{36}H_{52}N_2O_{23}$ K^+	880.2961	0.15%
A: 11 B: 1	12	$C_{36}H_{51}NO_{24}$ H^+	881.2801	0.21%
A: 12 B: 0	12	$C_{36}H_{50}O_{25}$ H^+	882.2641	0.16%
A: 6 B: 7	13	$C_{39}H_{61}N_7O_{20}$ K^+	947.3971	0.35%
A: 6 B: 7	13	$C_{39}H_{61}N_7O_{20}$ Na^+		
A: 7 B: 6	13	$C_{39}H_{60}N_6O_{21}$ Na^+	948.3812	0.50%
A: 7 B: 6	13	$C_{39}H_{60}N_6O_{21}$ K^+		
A: 7 B: 6	13	$C_{39}H_{60}N_6O_{21}$ H^+		
A: 8 B: 5	13	$C_{39}H_{59}N_5O_{22}$ H^+	949.3652	0.32%
A: 8 B: 5	13	$C_{39}H_{59}N_5O_{22}$ K^+		
A: 9 B: 4	13	$C_{39}H_{58}N_4O_{23}$ K^+	950.3492	0.16%
A: 10 B: 3	13	$C_{39}H_{57}N_3O_{24}$ K^+	951.3332	0.14%
A: 8 B: 6	14	$C_{42}H_{64}N_6O_{23}$ Na^+	1020.4023	0.13%
A: 10 B: 4	14	$C_{42}H_{62}N_4O_{25}$ K^+	1022.3703	0.13%
A: 10 B: 6	16	$C_{48}H_{72}N_6O_{27}$ H^+	1164.4445	0.15%
A: 11 B: 5	16	$C_{48}H_{71}N_5O_{28}$ H^+	1165.4286	0.12%

Analytical report

Page under construction...

aA MALDI, 1cyc, 1mg JEZ-1

Analysis parameters

Ionizations

MF	Monoisotopic mass	m/z mass
H^+	1.00783	1.00728
Na^+	22.98977	22.98922
K^+	38.96371	38.96316

End groups

α	ω	Color
H	OH	

Monomers

	Description	mf	Monoisotopic mass	min	max
A	Lactic acid	$C_3H_4O_2$	72.02113	0	100
B	Alanine	C_3H_5NO	71.03711	0	100

Experimental spectrum

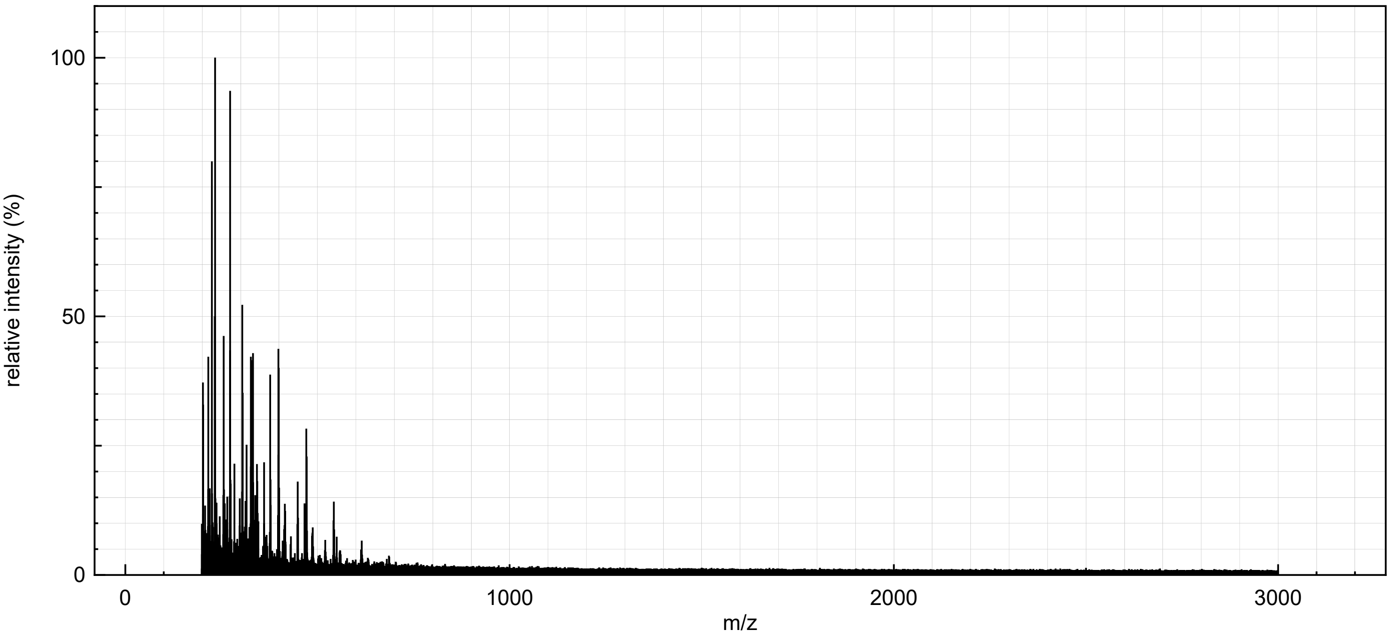

Reconstructed spectrum

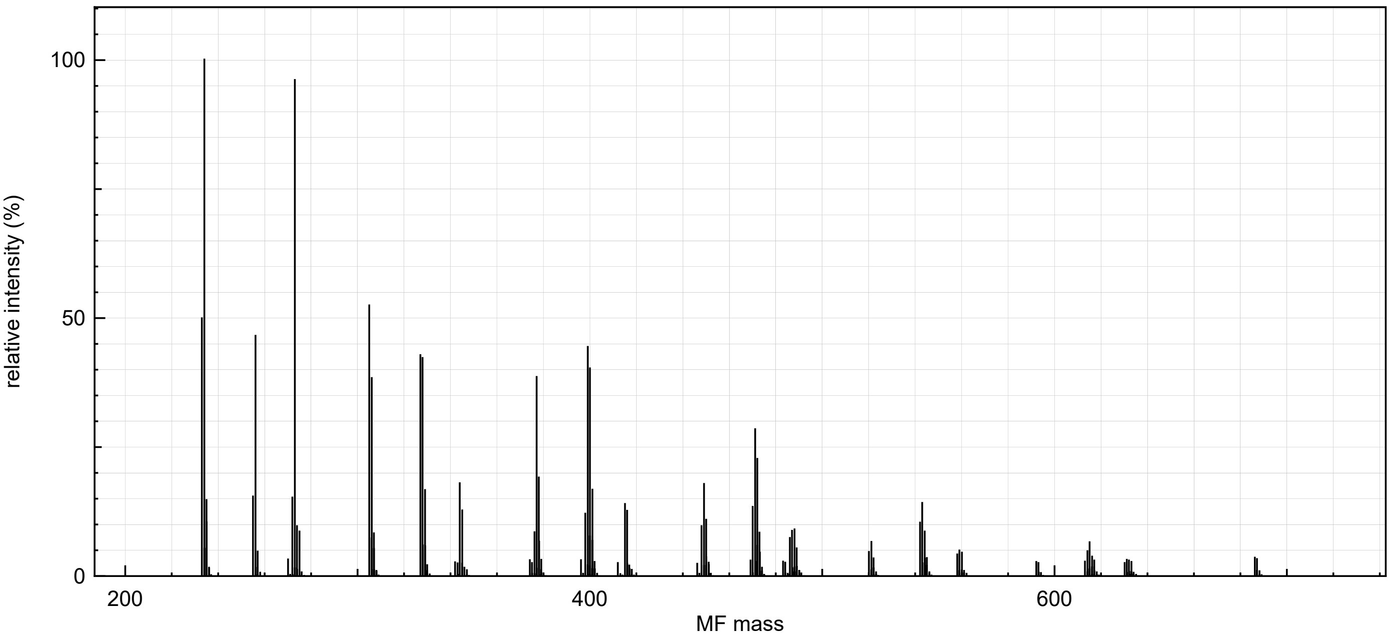

Error (ppm) versus m/z

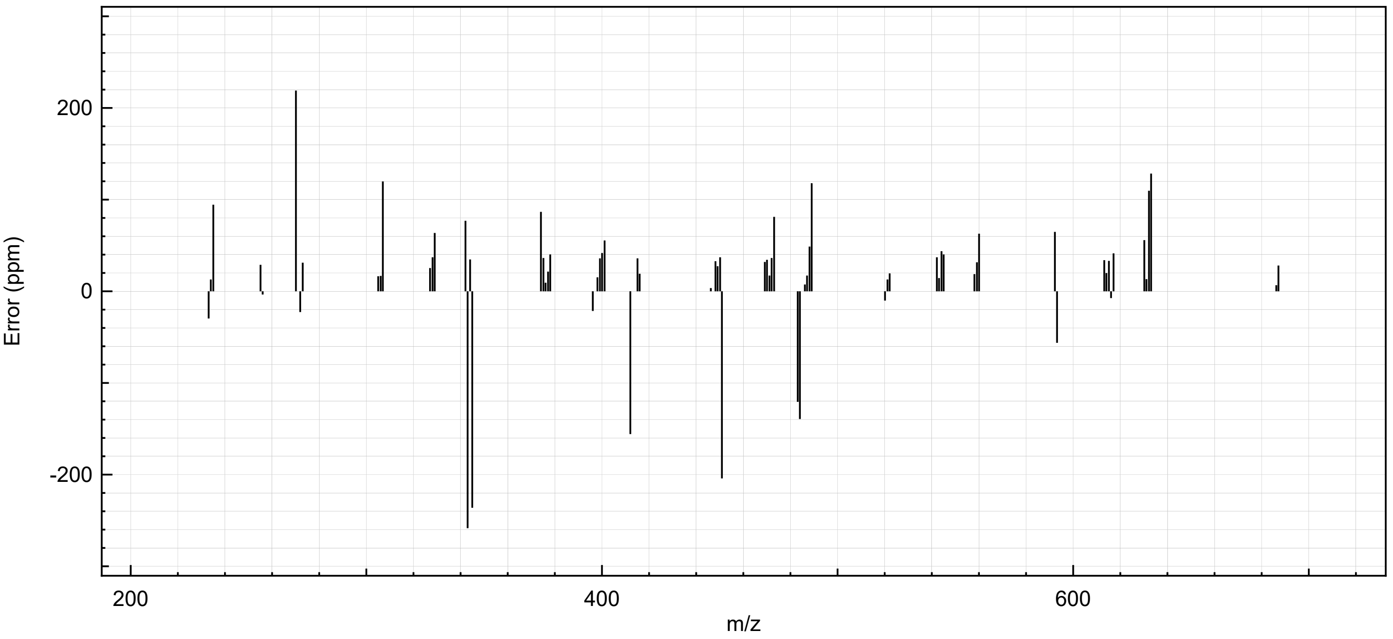

Analysis result

Percent of peaks covered: 21.98%
Number of assigned peaks: 70

Polymers [m/z]

Composition	Nb units	MF	Ioniz.	em [m/z]	Charge	Similarity	Quantity
A: 0 B: 3	3	$C_9H_{17}N_3O_4$	K^+	270.08506	1	36.28%	0.26%
A: 1 B: 2	3	$C_9H_{16}N_2O_5$	H^+	233.11320	1	38.71%	0.85%
A: 1 B: 2	3	$C_9H_{16}N_2O_5$	Na^+	255.09514	1	36.48%	0.47%
A: 2 B: 1	3	$C_9H_{15}NO_6$	H^+	234.09721	1	84.15%	0.68%
A: 2 B: 1	3	$C_9H_{15}NO_6$	Na^+	256.07916	1	67.59%	0.42%
A: 2 B: 1	3	$C_9H_{15}NO_6$	K^+	272.05310	1	30.67%	0.88%
A: 3 B: 0	3	$C_9H_{14}O_7$	K^+	273.03711	1	80.88%	0.82%
A: 3 B: 0	3	$C_9H_{14}O_7$	H^+	235.08123	1	58.74%	0.24%
A: 1 B: 3	4	$C_{12}H_{21}N_3O_6$	K^+	342.10619	1	35.28%	0.37%
A: 2 B: 2	4	$C_{12}H_{20}N_2O_7$	H^+	305.13433	1	56.44%	0.64%
A: 2 B: 2	4	$C_{12}H_{20}N_2O_7$	Na^+	327.11627	1	53.02%	0.67%
A: 2 B: 2	4	$C_{12}H_{20}N_2O_7$	K^+	343.09021	1	47.05%	0.49%
A: 3 B: 1	4	$C_{12}H_{19}NO_8$	H^+	306.11834	1	76.56%	0.43%
A: 3 B: 1	4	$C_{12}H_{19}NO_8$	Na^+	328.10029	1	68.35%	0.50%
A: 3 B: 1	4	$C_{12}H_{19}NO_8$	K^+	344.07422	1	54.42%	0.50%
A: 4 B: 0	4	$C_{12}H_{18}O_9$	H^+	307.10236	1	57.31%	0.22%
A: 4 B: 0	4	$C_{12}H_{18}O_9$	Na^+	329.08430	1	57.89%	0.32%
A: 4 B: 0	4	$C_{12}H_{18}O_9$	K^+	345.05824	1	67.30%	0.39%
A: 0 B: 5	5	$C_{15}H_{27}N_5O_6$	H^+	374.20341	1	46.97%	0.21%
A: 0 B: 5	5	$C_{15}H_{27}N_5O_6$	Na^+	396.18535	1	42.03%	0.22%
A: 0 B: 5	5	$C_{15}H_{27}N_5O_6$	K^+	412.15929	1	49.87%	0.19%
A: 1 B: 4	5	$C_{15}H_{26}N_4O_7$	H^+	375.18743	1	34.32%	0.35%
A: 2 B: 3	5	$C_{15}H_{25}N_3O_8$	H^+	376.17144	1	39.62%	0.43%
A: 2 B: 3	5	$C_{15}H_{25}N_3O_8$	Na^+	398.15339	1	36.47%	0.60%
A: 3 B: 2	5	$C_{15}H_{24}N_2O_9$	H^+	377.15546	1	63.32%	0.41%
A: 3 B: 2	5	$C_{15}H_{24}N_2O_9$	Na^+	399.13740	1	61.42%	0.60%
A: 3 B: 2	5	$C_{15}H_{24}N_2O_9$	K^+	415.11134	1	59.15%	0.33%
A: 4 B: 1	5	$C_{15}H_{23}NO_{10}$	H^+	378.13947	1	72.39%	0.27%
A: 4 B: 1	5	$C_{15}H_{23}NO_{10}$	Na^+	400.12142	1	72.65%	0.41%

Composition	Nb units	MF	Ioniz.	em [m/z]	Charge	Similarity	Quantity
A: 4 B: 1	5	$C_{15}H_{23}NO_{10}$	K^+	416.09535	1	64.64%	0.26%
A: 5 B: 0	5	$C_{15}H_{22}O_{11}$	Na^+	401.10543	1	66.14%	0.23%
A: 0 B: 6	6	$C_{18}H_{32}N_6O_7$	K^+	483.19641	1	55.95%	0.16%
A: 1 B: 5	6	$C_{18}H_{31}N_5O_8$	H^+	446.22454	1	51.37%	0.19%
A: 1 B: 5	6	$C_{18}H_{31}N_5O_8$	K^+	484.18042	1	53.79%	0.18%
A: 2 B: 4	6	$C_{18}H_{30}N_4O_9$	Na^+	469.19050	1	39.01%	0.35%
A: 3 B: 3	6	$C_{18}H_{29}N_3O_{10}$	H^+	448.19257	1	49.61%	0.27%
A: 3 B: 3	6	$C_{18}H_{29}N_3O_{10}$	Na^+	470.17451	1	47.39%	0.42%
A: 3 B: 3	6	$C_{18}H_{29}N_3O_{10}$	K^+	486.14845	1	54.00%	0.25%
A: 4 B: 2	6	$C_{18}H_{28}N_2O_{11}$	H^+	449.17659	1	64.95%	0.25%
A: 4 B: 2	6	$C_{18}H_{28}N_2O_{11}$	Na^+	471.15853	1	63.81%	0.39%
A: 4 B: 2	6	$C_{18}H_{28}N_2O_{11}$	K^+	487.13247	1	60.53%	0.25%
A: 5 B: 1	6	$C_{18}H_{27}NO_{12}$	H^+	450.16060	1	62.31%	0.19%
A: 5 B: 1	6	$C_{18}H_{27}NO_{12}$	Na^+	472.14255	1	68.98%	0.28%
A: 5 B: 1	6	$C_{18}H_{27}NO_{12}$	K^+	488.11648	1	62.96%	0.22%
A: 6 B: 0	6	$C_{18}H_{26}O_{13}$	H^+	451.14462	1	56.35%	0.16%
A: 6 B: 0	6	$C_{18}H_{26}O_{13}$	Na^+	473.12656	1	61.70%	0.20%
A: 6 B: 0	6	$C_{18}H_{26}O_{13}$	K^+	489.10050	1	60.79%	0.20%
A: 4 B: 3	7	$C_{21}H_{33}N_3O_{12}$	H^+	520.21370	1	56.72%	0.21%
A: 4 B: 3	7	$C_{21}H_{33}N_3O_{12}$	Na^+	542.19564	1	55.67%	0.29%
A: 4 B: 3	7	$C_{21}H_{33}N_3O_{12}$	K^+	558.16958	1	61.41%	0.21%
A: 5 B: 2	7	$C_{21}H_{32}N_2O_{13}$	H^+	521.19772	1	64.75%	0.20%
A: 5 B: 2	7	$C_{21}H_{32}N_2O_{13}$	Na^+	543.17966	1	67.56%	0.26%
A: 5 B: 2	7	$C_{21}H_{32}N_2O_{13}$	K^+	559.15360	1	61.73%	0.19%
A: 6 B: 1	7	$C_{21}H_{31}NO_{14}$	H^+	522.18173	1	57.32%	0.17%
A: 6 B: 1	7	$C_{21}H_{31}NO_{14}$	Na^+	544.16368	1	63.34%	0.20%
A: 6 B: 1	7	$C_{21}H_{31}NO_{14}$	K^+	560.13761	1	64.43%	0.18%
A: 7 B: 0	7	$C_{21}H_{30}O_{15}$	Na^+	545.14769	1	60.74%	0.17%
A: 4 B: 4	8	$C_{24}H_{38}N_4O_{13}$	Na^+	613.23276	1	54.20%	0.21%
A: 5 B: 3	8	$C_{24}H_{37}N_3O_{14}$	H^+	592.23483	1	60.80%	0.16%
A: 5 B: 3	8	$C_{24}H_{37}N_3O_{14}$	Na^+	614.21677	1	60.73%	0.21%
A: 5 B: 3	8	$C_{24}H_{37}N_3O_{14}$	K^+	630.19071	1	61.24%	0.17%
A: 6 B: 2	8	$C_{24}H_{36}N_2O_{15}$	H^+	593.21884	1	61.01%	0.17%
A: 6 B: 2	8	$C_{24}H_{36}N_2O_{15}$	Na^+	615.20079	1	64.42%	0.19%

Composition	Nb units	MF	Ioniz.	em [m/z]	Charge	Similarity	Quantity
A: 6 B: 2	8	$C_{24}H_{36}N_2O_{15}$	K^+	631.17473	1	64.03%	0.17%
A: 7 B: 1	8	$C_{24}H_{35}NO_{16}$	Na^+	616.18480	1	63.04%	0.17%
A: 7 B: 1	8	$C_{24}H_{35}NO_{16}$	K^+	632.15874	1	65.36%	0.16%
A: 8 B: 0	8	$C_{24}H_{34}O_{17}$	Na^+	617.16882	1	59.03%	0.16%
A: 8 B: 0	8	$C_{24}H_{34}O_{17}$	K^+	633.14276	1	63.80%	0.16%
A: 6 B: 3	9	$C_{27}H_{41}N_3O_{16}$	Na^+	686.23790	1	63.46%	0.17%
A: 7 B: 2	9	$C_{27}H_{40}N_2O_{17}$	Na^+	687.22192	1	64.00%	0.16%

Polymers grouped by monoisotopic mass

Composition				Monoisotopic mass	Quantity
A: 0 B: 3	3	$C_9H_{17}N_3O_4$	K^+	231.1219	0.26%
A: 1 B: 2<br>A: 1 B: 2	3<br>3	$C_9H_{16}N_2O_5$<br>$C_9H_{16}N_2O_5$	H^+<br>Na^+	232.1059	1.32%
A: 2 B: 1<br>A: 2 B: 1<br>A: 2 B: 1	3<br>3<br>3	$C_9H_{15}NO_6$<br>$C_9H_{15}NO_6$<br>$C_9H_{15}NO_6$	H^+<br>Na^+<br>K^+	233.0899	1.98%
A: 3 B: 0<br>A: 3 B: 0	3<br>3	$C_9H_{14}O_7$<br>$C_9H_{14}O_7$	K^+<br>H^+	234.0740	1.06%
A: 1 B: 3	4	$C_{12}H_{21}N_3O_6$	K^+	303.1430	0.37%
A: 2 B: 2<br>A: 2 B: 2<br>A: 2 B: 2	4<br>4<br>4	$C_{12}H_{20}N_2O_7$<br>$C_{12}H_{20}N_2O_7$<br>$C_{12}H_{20}N_2O_7$	H^+<br>Na^+<br>K^+	304.1271	1.80%
A: 3 B: 1<br>A: 3 B: 1<br>A: 3 B: 1	4<br>4<br>4	$C_{12}H_{19}NO_8$<br>$C_{12}H_{19}NO_8$<br>$C_{12}H_{19}NO_8$	H^+<br>Na^+<br>K^+	305.1111	1.43%
A: 4 B: 0<br>A: 4 B: 0<br>A: 4 B: 0	4<br>4<br>4	$C_{12}H_{18}O_9$<br>$C_{12}H_{18}O_9$<br>$C_{12}H_{18}O_9$	K^+<br>Na^+<br>H^+	306.0951	0.94%
A: 0 B: 5<br>A: 0 B: 5<br>A: 0 B: 5	5<br>5<br>5	$C_{15}H_{27}N_5O_6$<br>$C_{15}H_{27}N_5O_6$<br>$C_{15}H_{27}N_5O_6$	K^+<br>H^+<br>Na^+	373.1961	0.62%
A: 1 B: 4	5	$C_{15}H_{26}N_4O_7$	H^+	374.1801	0.35%
A: 2 B: 3<br>A: 2 B: 3	5<br>5	$C_{15}H_{25}N_3O_8$<br>$C_{15}H_{25}N_3O_8$	H^+<br>Na^+	375.1642	1.03%
A: 3 B: 2<br>A: 3 B: 2<br>A: 3 B: 2	5<br>5<br>5	$C_{15}H_{24}N_2O_9$<br>$C_{15}H_{24}N_2O_9$<br>$C_{15}H_{24}N_2O_9$	H^+<br>Na^+<br>K^+	376.1482	1.34%

Composition					Monoisotopic mass	Quantity
A: 4	B: 1	5	$C_{15}H_{23}NO_{10}$	Na^+	377.1322	0.94%
A: 4	B: 1	5	$C_{15}H_{23}NO_{10}$	H^+		
A: 4	B: 1	5	$C_{15}H_{23}NO_{10}$	K^+		
A: 5	B: 0	5	$C_{15}H_{22}O_{11}$	Na^+	378.1162	0.23%
A: 0	B: 6	6	$C_{18}H_{32}N_6O_7$	K^+	444.2332	0.16%
A: 1	B: 5	6	$C_{18}H_{31}N_5O_8$	K^+	445.2173	0.37%
A: 1	B: 5	6	$C_{18}H_{31}N_5O_8$	H^+		
A: 2	B: 4	6	$C_{18}H_{30}N_4O_9$	Na^+	446.2013	0.35%
A: 3	B: 3	6	$C_{18}H_{29}N_3O_{10}$	K^+	447.1853	0.94%
A: 3	B: 3	6	$C_{18}H_{29}N_3O_{10}$	H^+		
A: 3	B: 3	6	$C_{18}H_{29}N_3O_{10}$	Na^+		
A: 4	B: 2	6	$C_{18}H_{28}N_2O_{11}$	H^+	448.1693	0.89%
A: 4	B: 2	6	$C_{18}H_{28}N_2O_{11}$	Na^+		
A: 4	B: 2	6	$C_{18}H_{28}N_2O_{11}$	K^+		
A: 5	B: 1	6	$C_{18}H_{27}NO_{12}$	Na^+	449.1533	0.69%
A: 5	B: 1	6	$C_{18}H_{27}NO_{12}$	K^+		
A: 5	B: 1	6	$C_{18}H_{27}NO_{12}$	H^+		
A: 6	B: 0	6	$C_{18}H_{26}O_{13}$	Na^+	450.1373	0.56%
A: 6	B: 0	6	$C_{18}H_{26}O_{13}$	K^+		
A: 6	B: 0	6	$C_{18}H_{26}O_{13}$	H^+		
A: 4	B: 3	7	$C_{21}H_{33}N_3O_{12}$	K^+	519.2064	0.71%
A: 4	B: 3	7	$C_{21}H_{33}N_3O_{12}$	H^+		
A: 4	B: 3	7	$C_{21}H_{33}N_3O_{12}$	Na^+		
A: 5	B: 2	7	$C_{21}H_{32}N_2O_{13}$	Na^+	520.1904	0.65%
A: 5	B: 2	7	$C_{21}H_{32}N_2O_{13}$	H^+		
A: 5	B: 2	7	$C_{21}H_{32}N_2O_{13}$	K^+		
A: 6	B: 1	7	$C_{21}H_{31}NO_{14}$	K^+	521.1745	0.55%
A: 6	B: 1	7	$C_{21}H_{31}NO_{14}$	Na^+		
A: 6	B: 1	7	$C_{21}H_{31}NO_{14}$	H^+		
A: 7	B: 0	7	$C_{21}H_{30}O_{15}$	Na^+	522.1585	0.17%
A: 4	B: 4	8	$C_{24}H_{38}N_4O_{13}$	Na^+	590.2435	0.21%
A: 5	B: 3	8	$C_{24}H_{37}N_3O_{14}$	K^+	591.2276	0.55%
A: 5	B: 3	8	$C_{24}H_{37}N_3O_{14}$	H^+		
A: 5	B: 3	8	$C_{24}H_{37}N_3O_{14}$	Na^+		
A: 6	B: 2	8	$C_{24}H_{36}N_2O_{15}$	Na^+	592.2116	0.53%
A: 6	B: 2	8	$C_{24}H_{36}N_2O_{15}$	K^+		
A: 6	B: 2	8	$C_{24}H_{36}N_2O_{15}$	H^+		

Composition					Monoisotopic mass	Quantity
A: 7	B: 1	8	$C_{24}H_{35}NO_{16}$	K^+	593.1956	0.33%
A: 7	B: 1	8	$C_{24}H_{35}NO_{16}$	Na^+		
A: 8	B: 0	8	$C_{24}H_{34}O_{17}$	K^+	594.1796	0.31%
A: 8	B: 0	8	$C_{24}H_{34}O_{17}$	Na^+		
A: 6	B: 3	9	$C_{27}H_{41}N_3O_{16}$	Na^+	663.2487	0.17%
A: 7	B: 2	9	$C_{27}H_{40}N_2O_{17}$	Na^+	664.2327	0.16%

Analytical report

Page under construction...

aA MALDI, 4cyc, 1mg JEZ-1

Analysis parameters

Ionizations

MF	Monoisotopic mass	m/z mass
H^+	1.00783	1.00728
Na^+	22.98977	22.98922
K^+	38.96371	38.96316

End groups

α	ω	Color
H	OH	

Monomers

	Description	mf	Monoisotopic mass	min	max
A	Lactic acid	$C_3H_4O_2$	72.02113	0	100
B	Alanine	C_3H_5NO	71.03711	0	100

Experimental spectrum

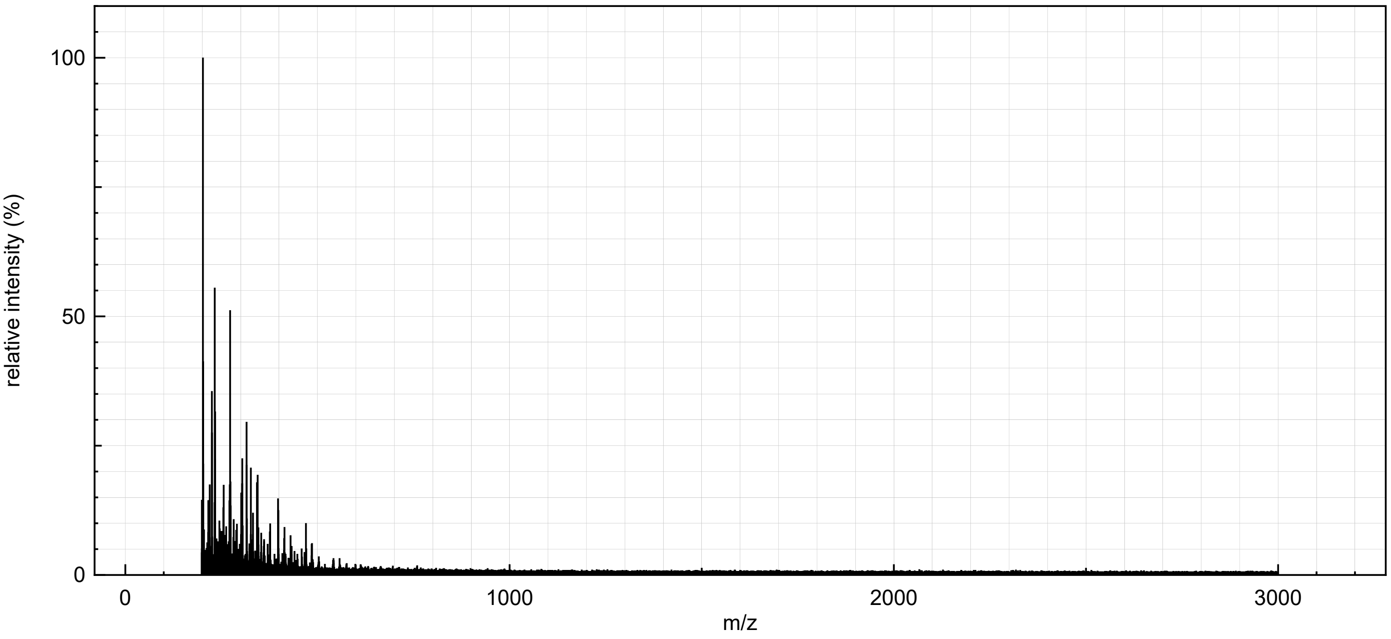

Reconstructed spectrum

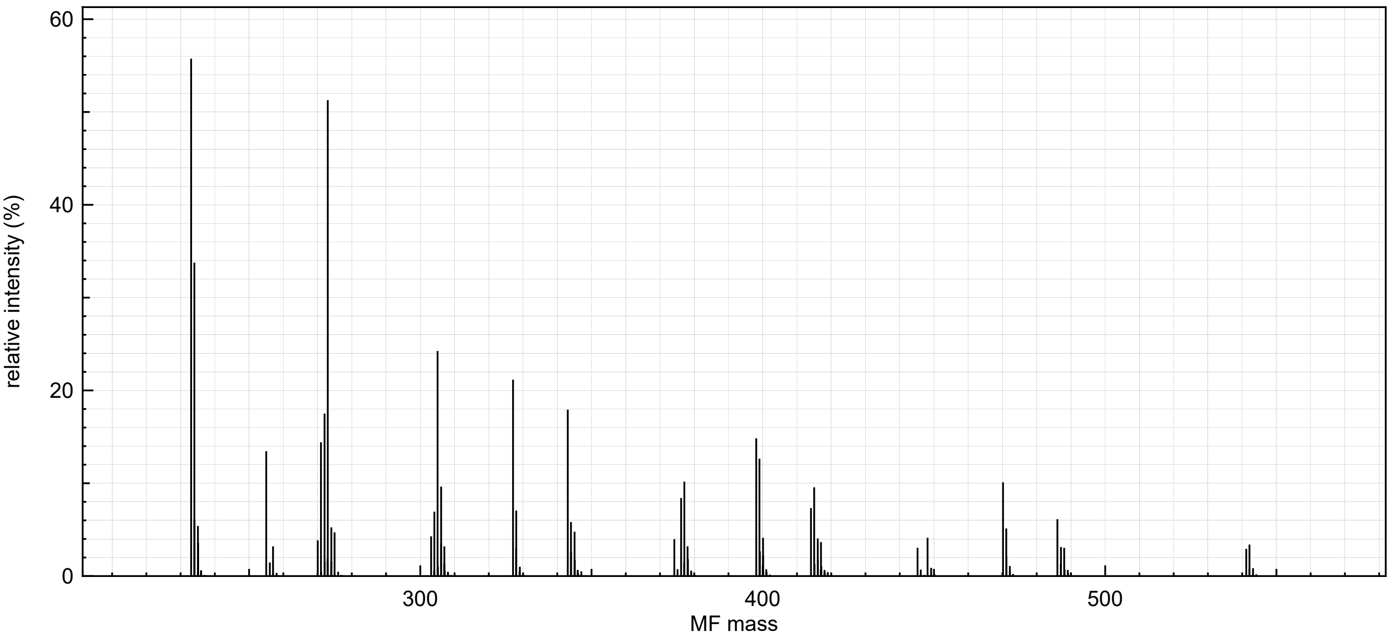

Error (ppm) versus m/z

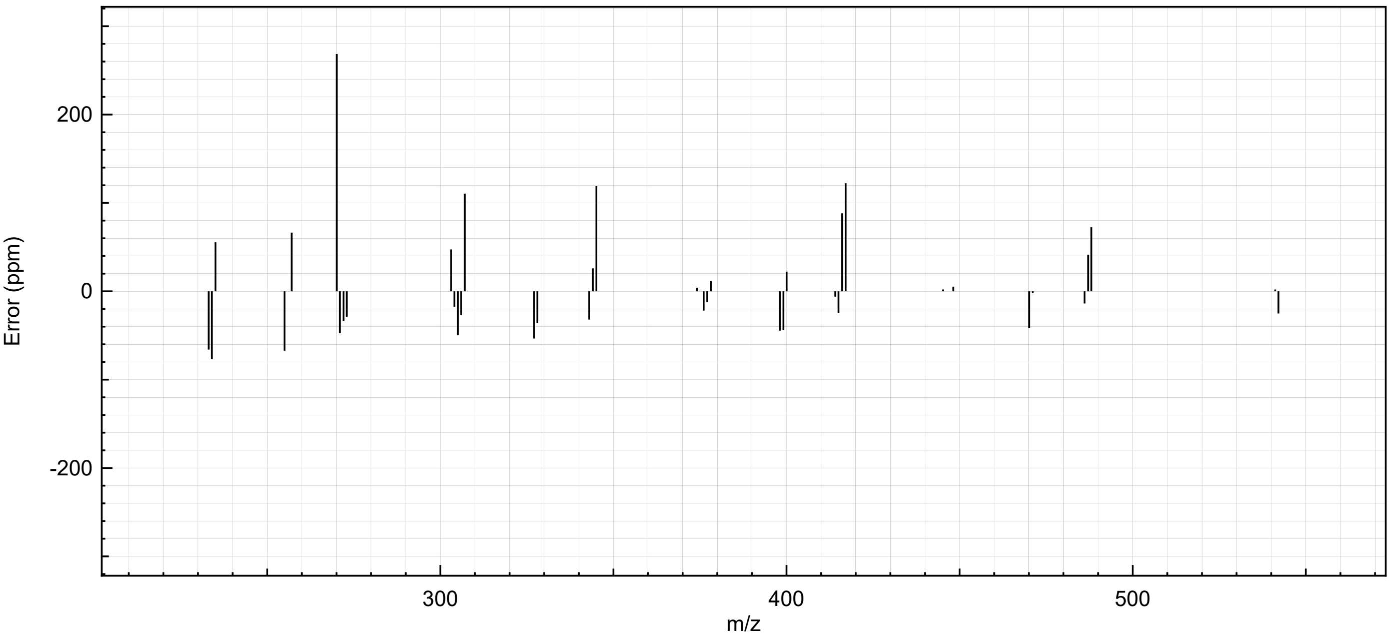

Analysis result

Percent of peaks covered: 14.35%
Number of assigned peaks: 39

Polymers [m/z]

Composition	Nb units	MF	Ioniz.	em [m/z]	Charge	Similarity	Quantity
A: 0 B: 3	3	$C_9H_{17}N_3O_4$	K^+	270.08506	1	31.43%	0.43%
A: 1 B: 2	3	$C_9H_{16}N_2O_5$	H^+	233.11320	1	60.32%	0.89%
A: 1 B: 2	3	$C_9H_{16}N_2O_5$	Na^+	255.09514	1	48.32%	0.40%
A: 1 B: 2	3	$C_9H_{16}N_2O_5$	K^+	271.06908	1	37.14%	0.81%
A: 2 B: 1	3	$C_9H_{15}NO_6$	H^+	234.09721	1	77.76%	0.52%
A: 2 B: 1	3	$C_9H_{15}NO_6$	K^+	272.05310	1	37.66%	0.91%
A: 3 B: 0	3	$C_9H_{14}O_7$	K^+	273.03711	1	71.58%	0.79%
A: 3 B: 0	3	$C_9H_{14}O_7$	H^+	235.08123	1	46.61%	0.26%
A: 3 B: 0	3	$C_9H_{14}O_7$	Na^+	257.06317	1	38.27%	0.27%
A: 0 B: 4	4	$C_{12}H_{22}N_4O_5$	H^+	303.16630	1	30.76%	0.46%
A: 1 B: 3	4	$C_{12}H_{21}N_3O_6$	H^+	304.15031	1	33.22%	0.52%
A: 2 B: 2	4	$C_{12}H_{20}N_2O_7$	H^+	305.13433	1	69.76%	0.50%
A: 2 B: 2	4	$C_{12}H_{20}N_2O_7$	Na^+	327.11627	1	61.21%	0.44%
A: 2 B: 2	4	$C_{12}H_{20}N_2O_7$	K^+	343.09021	1	50.67%	0.53%
A: 3 B: 1	4	$C_{12}H_{19}NO_8$	H^+	306.11834	1	62.97%	0.30%
A: 3 B: 1	4	$C_{12}H_{19}NO_8$	Na^+	328.10029	1	48.17%	0.35%
A: 3 B: 1	4	$C_{12}H_{19}NO_8$	K^+	344.07422	1	41.93%	0.55%
A: 4 B: 0	4	$C_{12}H_{18}O_9$	H^+	307.10236	1	52.33%	0.22%
A: 4 B: 0	4	$C_{12}H_{18}O_9$	K^+	345.05824	1	63.72%	0.53%
A: 0 B: 5	5	$C_{15}H_{27}N_5O_6$	H^+	374.20341	1	47.00%	0.22%
A: 2 B: 3	5	$C_{15}H_{25}N_3O_8$	H^+	376.17144	1	51.27%	0.31%
A: 2 B: 3	5	$C_{15}H_{25}N_3O_8$	Na^+	398.15339	1	55.56%	0.36%
A: 2 B: 3	5	$C_{15}H_{25}N_3O_8$	K^+	414.12732	1	54.70%	0.32%
A: 3 B: 2	5	$C_{15}H_{24}N_2O_9$	H^+	377.15546	1	67.38%	0.27%
A: 3 B: 2	5	$C_{15}H_{24}N_2O_9$	Na^+	399.13740	1	67.71%	0.29%
A: 3 B: 2	5	$C_{15}H_{24}N_2O_9$	K^+	415.11134	1	61.92%	0.31%
A: 4 B: 1	5	$C_{15}H_{23}NO_{10}$	H^+	378.13947	1	57.44%	0.18%
A: 4 B: 1	5	$C_{15}H_{23}NO_{10}$	Na^+	400.12142	1	58.82%	0.20%
A: 4 B: 1	5	$C_{15}H_{23}NO_{10}$	K^+	416.09535	1	57.95%	0.25%

Composition	Nb units	MF	Ioniz.	em [m/z]	Charge	Similarity	Quantity
A: 5 B: 0	5	$C_{15}H_{22}O_{11}$	K^+	417.07937	1	64.87%	0.21%
A: 0 B: 6	6	$C_{18}H_{32}N_6O_7$	H^+	445.24052	1	58.02%	0.16%
A: 3 B: 3	6	$C_{18}H_{29}N_3O_{10}$	H^+	448.19257	1	60.52%	0.19%
A: 3 B: 3	6	$C_{18}H_{29}N_3O_{10}$	Na^+	470.17451	1	66.34%	0.24%
A: 3 B: 3	6	$C_{18}H_{29}N_3O_{10}$	K^+	486.14845	1	65.55%	0.24%
A: 4 B: 2	6	$C_{18}H_{28}N_2O_{11}$	Na^+	471.15853	1	61.63%	0.18%
A: 4 B: 2	6	$C_{18}H_{28}N_2O_{11}$	K^+	487.13247	1	55.73%	0.20%
A: 5 B: 1	6	$C_{18}H_{27}NO_{12}$	K^+	488.11648	1	66.40%	0.19%
A: 3 B: 4	7	$C_{21}H_{34}N_4O_{11}$	Na^+	541.21163	1	57.68%	0.19%
A: 4 B: 3	7	$C_{21}H_{33}N_3O_{12}$	Na^+	542.19564	1	62.12%	0.18%

Polymers grouped by monoisotopic mass

Composition				Monoisotopic mass	Quantity
A: 0 B: 3	3	$C_9H_{17}N_3O_4$	K^+	231.1219	0.43%
A: 1 B: 2<br>A: 1 B: 2<br>A: 1 B: 2	3<br>3<br>3	$C_9H_{16}N_2O_5$<br>$C_9H_{16}N_2O_5$<br>$C_9H_{16}N_2O_5$	H^+<br>Na^+<br>K^+	232.1059	2.10%
A: 2 B: 1<br>A: 2 B: 1	3<br>3	$C_9H_{15}NO_6$<br>$C_9H_{15}NO_6$	H^+<br>K^+	233.0899	1.44%
A: 3 B: 0<br>A: 3 B: 0<br>A: 3 B: 0	3<br>3<br>3	$C_9H_{14}O_7$<br>$C_9H_{14}O_7$<br>$C_9H_{14}O_7$	K^+<br>H^+<br>Na^+	234.0740	1.33%
A: 0 B: 4	4	$C_{12}H_{22}N_4O_5$	H^+	302.1590	0.46%
A: 1 B: 3	4	$C_{12}H_{21}N_3O_6$	H^+	303.1430	0.52%
A: 2 B: 2<br>A: 2 B: 2<br>A: 2 B: 2	4<br>4<br>4	$C_{12}H_{20}N_2O_7$<br>$C_{12}H_{20}N_2O_7$<br>$C_{12}H_{20}N_2O_7$	H^+<br>Na^+<br>K^+	304.1271	1.47%
A: 3 B: 1<br>A: 3 B: 1<br>A: 3 B: 1	4<br>4<br>4	$C_{12}H_{19}NO_8$<br>$C_{12}H_{19}NO_8$<br>$C_{12}H_{19}NO_8$	H^+<br>Na^+<br>K^+	305.1111	1.19%
A: 4 B: 0<br>A: 4 B: 0	4<br>4	$C_{12}H_{18}O_9$<br>$C_{12}H_{18}O_9$	K^+<br>H^+	306.0951	0.75%
A: 0 B: 5	5	$C_{15}H_{27}N_5O_6$	H^+	373.1961	0.22%
A: 2 B: 3<br>A: 2 B: 3<br>A: 2 B: 3	5<br>5<br>5	$C_{15}H_{25}N_3O_8$<br>$C_{15}H_{25}N_3O_8$<br>$C_{15}H_{25}N_3O_8$	Na^+<br>K^+<br>H^+	375.1642	0.99%

Composition				Monoisotopic mass	Quantity
A: 3 B: 2	5	$C_{15}H_{24}N_2O_9$	Na^+	376.1482	0.86%
A: 3 B: 2	5	$C_{15}H_{24}N_2O_9$	H^+		
A: 3 B: 2	5	$C_{15}H_{24}N_2O_9$	K^+		
A: 4 B: 1	5	$C_{15}H_{23}NO_{10}$	Na^+	377.1322	0.63%
A: 4 B: 1	5	$C_{15}H_{23}NO_{10}$	K^+		
A: 4 B: 1	5	$C_{15}H_{23}NO_{10}$	H^+		
A: 5 B: 0	5	$C_{15}H_{22}O_{11}$	K^+	378.1162	0.21%
A: 0 B: 6	6	$C_{18}H_{32}N_6O_7$	H^+	444.2332	0.16%
A: 3 B: 3	6	$C_{18}H_{29}N_3O_{10}$	Na^+	447.1853	0.67%
A: 3 B: 3	6	$C_{18}H_{29}N_3O_{10}$	K^+		
A: 3 B: 3	6	$C_{18}H_{29}N_3O_{10}$	H^+		
A: 4 B: 2	6	$C_{18}H_{28}N_2O_{11}$	Na^+	448.1693	0.38%
A: 4 B: 2	6	$C_{18}H_{28}N_2O_{11}$	K^+		
A: 5 B: 1	6	$C_{18}H_{27}NO_{12}$	K^+	449.1533	0.19%
A: 3 B: 4	7	$C_{21}H_{34}N_4O_{11}$	Na^+	518.2224	0.19%
A: 4 B: 3	7	$C_{21}H_{33}N_3O_{12}$	Na^+	519.2064	0.18%

Analytical report

Page under construction...

Analysis parameters

aA MALDI, 1cyc, 1mg LHS-1D

Ionizations

MF	Monoisotopic mass	m/z mass
H^+	1.00783	1.00728
Na^+	22.98977	22.98922
K^+	38.96371	38.96316

End groups

α	ω	Color
H	OH	

Monomers

	Description	mf	Monoisotopic mass	min	max
A	Lactic acid	$C_3H_4O_2$	72.02113	0	100
B	Alanine	C_3H_5NO	71.03711	0	100

Experimental spectrum

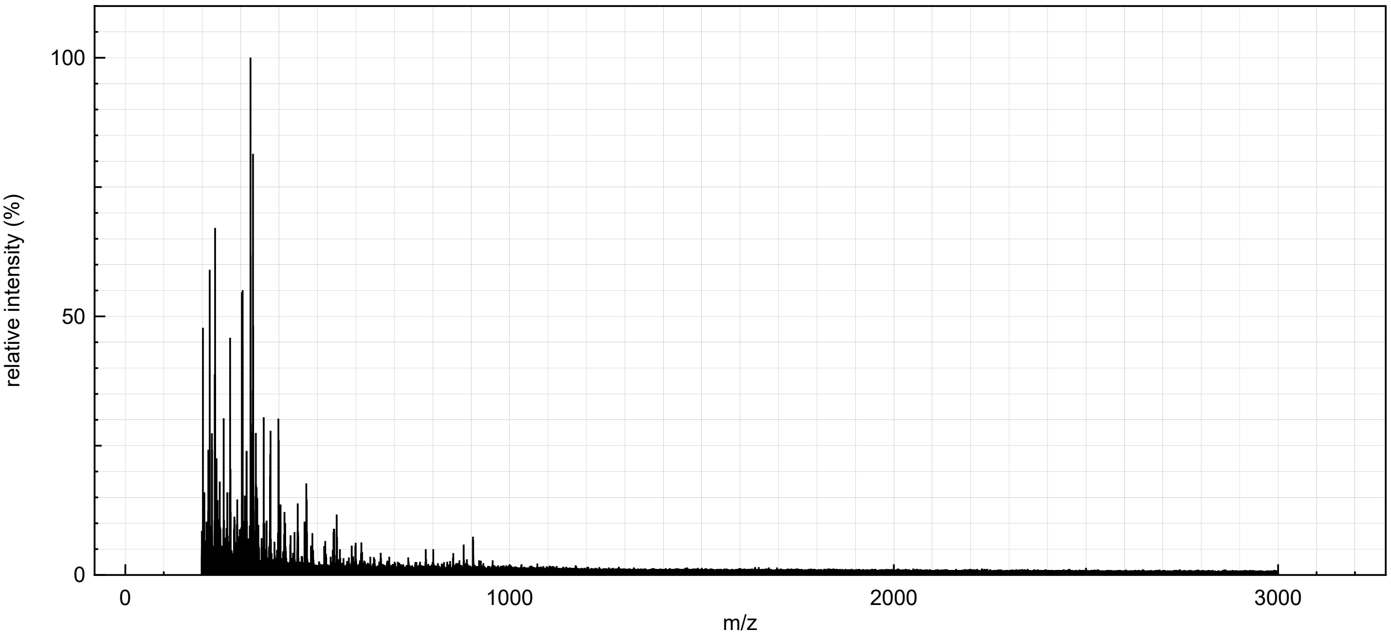

Reconstructed spectrum

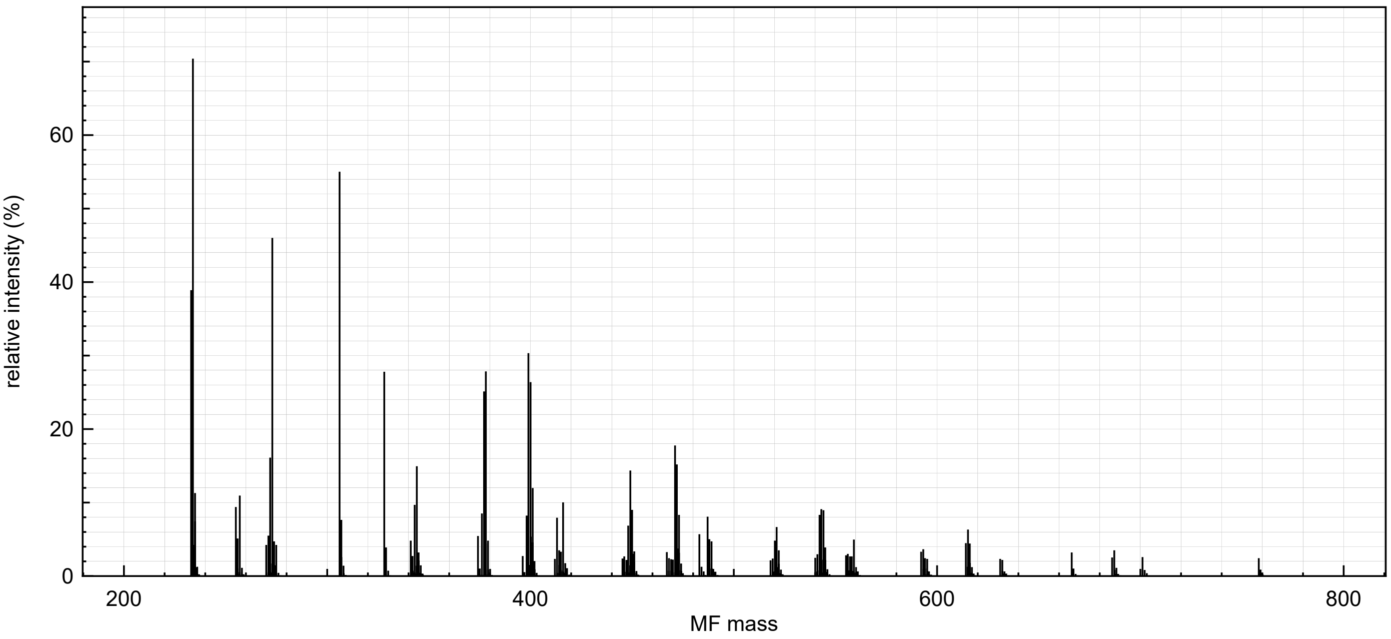

Error (ppm) versus m/z

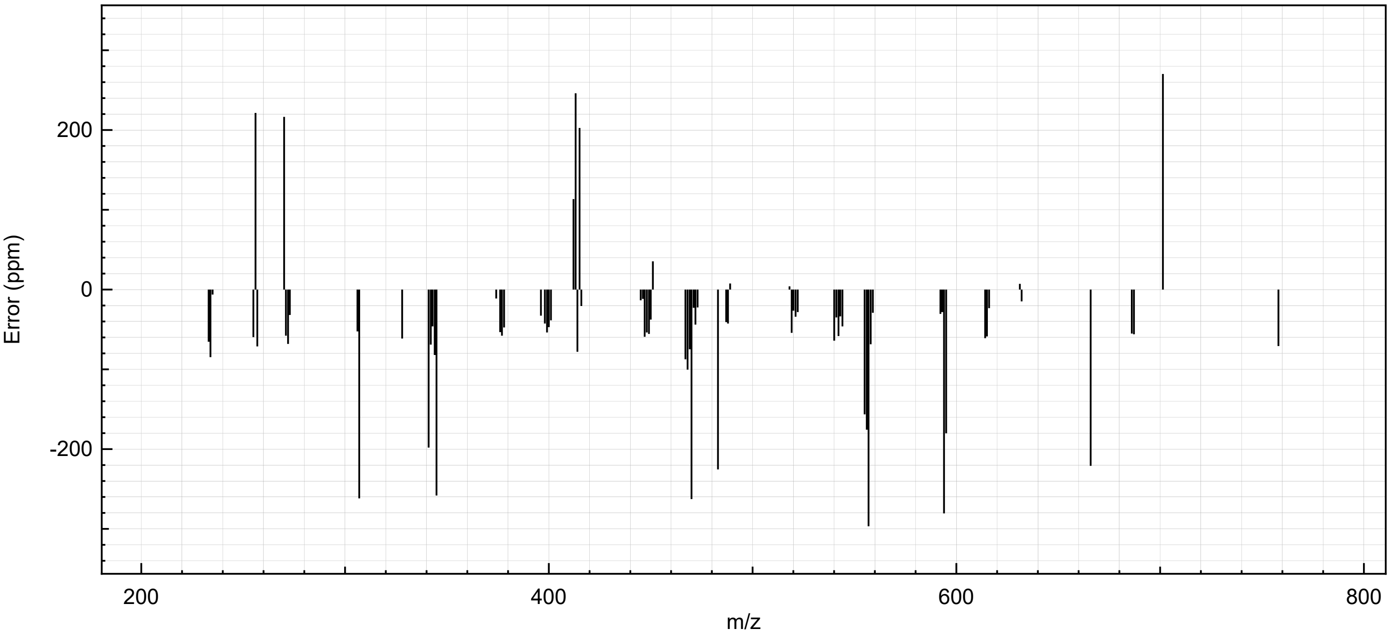

Analysis result

Percent of peaks covered: 21.17%
Number of assigned peaks: 80

Polymers [m/z]

Composition	Nb units	MF	Ioniz.	em [m/z]	Charge	Similarity	Quantity
A: 0 B: 3	3	$C_9H_{17}N_3O_4$	K^+	270.08506	1	41.01%	0.28%
A: 1 B: 2	3	$C_9H_{16}N_2O_5$	H^+	233.11320	1	39.41%	0.75%
A: 1 B: 2	3	$C_9H_{16}N_2O_5$	Na^+	255.09514	1	34.56%	0.40%
A: 1 B: 2	3	$C_9H_{16}N_2O_5$	K^+	271.06908	1	30.70%	0.51%
A: 2 B: 1	3	$C_9H_{15}NO_6$	H^+	234.09721	1	82.95%	0.61%
A: 2 B: 1	3	$C_9H_{15}NO_6$	Na^+	256.07916	1	65.58%	0.36%
A: 2 B: 1	3	$C_9H_{15}NO_6$	K^+	272.05310	1	38.41%	0.62%
A: 3 B: 0	3	$C_9H_{14}O_7$	H^+	235.08123	1	55.22%	0.23%
A: 3 B: 0	3	$C_9H_{14}O_7$	Na^+	257.06317	1	55.21%	0.23%
A: 3 B: 0	3	$C_9H_{14}O_7$	K^+	273.03711	1	69.92%	0.55%
A: 0 B: 4	4	$C_{12}H_{22}N_4O_5$	K^+	341.12218	1	47.37%	0.37%
A: 1 B: 3	4	$C_{12}H_{21}N_3O_6$	K^+	342.10619	1	53.31%	0.40%
A: 2 B: 2	4	$C_{12}H_{20}N_2O_7$	K^+	343.09021	1	50.53%	0.36%
A: 3 B: 1	4	$C_{12}H_{19}NO_8$	H^+	306.11834	1	75.30%	0.47%
A: 3 B: 1	4	$C_{12}H_{19}NO_8$	Na^+	328.10029	1	63.45%	0.48%
A: 3 B: 1	4	$C_{12}H_{19}NO_8$	K^+	344.07422	1	51.65%	0.40%
A: 4 B: 0	4	$C_{12}H_{18}O_9$	H^+	307.10236	1	57.10%	0.24%
A: 4 B: 0	4	$C_{12}H_{18}O_9$	K^+	345.05824	1	50.34%	0.36%
A: 0 B: 5	5	$C_{15}H_{27}N_5O_6$	H^+	374.20341	1	51.94%	0.21%
A: 0 B: 5	5	$C_{15}H_{27}N_5O_6$	Na^+	396.18535	1	52.78%	0.18%
A: 0 B: 5	5	$C_{15}H_{27}N_5O_6$	K^+	412.15929	1	55.41%	0.20%
A: 1 B: 4	5	$C_{15}H_{26}N_4O_7$	K^+	413.14331	1	52.75%	0.22%
A: 2 B: 3	5	$C_{15}H_{25}N_3O_8$	H^+	376.17144	1	37.83%	0.46%
A: 2 B: 3	5	$C_{15}H_{25}N_3O_8$	Na^+	398.15339	1	35.02%	0.40%
A: 2 B: 3	5	$C_{15}H_{25}N_3O_8$	K^+	414.12732	1	46.18%	0.26%
A: 3 B: 2	5	$C_{15}H_{24}N_2O_9$	H^+	377.15546	1	62.73%	0.44%
A: 3 B: 2	5	$C_{15}H_{24}N_2O_9$	Na^+	399.13740	1	55.85%	0.44%
A: 3 B: 2	5	$C_{15}H_{24}N_2O_9$	K^+	415.11134	1	54.34%	0.29%
A: 4 B: 1	5	$C_{15}H_{23}NO_{10}$	H^+	378.13947	1	73.38%	0.29%

Composition	Nb units	MF	Ioniz.	em [m/z]	Charge	Similarity	Quantity
A: 4 B: 1	5	$C_{15}H_{23}NO_{10}$	Na^+	400.12142	1	66.97%	0.33%
A: 4 B: 1	5	$C_{15}H_{23}NO_{10}$	K^+	416.09535	1	60.69%	0.26%
A: 5 B: 0	5	$C_{15}H_{22}O_{11}$	Na^+	401.10543	1	65.99%	0.21%
A: 0 B: 6	6	$C_{18}H_{32}N_6O_7$	H^+	445.24052	1	54.24%	0.13%
A: 0 B: 6	6	$C_{18}H_{32}N_6O_7$	Na^+	467.22247	1	58.21%	0.22%
A: 0 B: 6	6	$C_{18}H_{32}N_6O_7$	K^+	483.19641	1	58.78%	0.19%
A: 1 B: 5	6	$C_{18}H_{31}N_5O_8$	H^+	446.22454	1	48.97%	0.16%
A: 1 B: 5	6	$C_{18}H_{31}N_5O_8$	Na^+	468.20648	1	54.54%	0.22%
A: 2 B: 4	6	$C_{18}H_{30}N_4O_9$	H^+	447.20856	1	39.04%	0.23%
A: 2 B: 4	6	$C_{18}H_{30}N_4O_9$	Na^+	469.19050	1	43.62%	0.27%
A: 3 B: 3	6	$C_{18}H_{29}N_3O_{10}$	H^+	448.19257	1	46.17%	0.28%
A: 3 B: 3	6	$C_{18}H_{29}N_3O_{10}$	Na^+	470.17451	1	43.99%	0.33%
A: 4 B: 2	6	$C_{18}H_{28}N_2O_{11}$	H^+	449.17659	1	63.55%	0.26%
A: 4 B: 2	6	$C_{18}H_{28}N_2O_{11}$	Na^+	471.15853	1	59.37%	0.34%
A: 4 B: 2	6	$C_{18}H_{28}N_2O_{11}$	K^+	487.13247	1	59.64%	0.21%
A: 5 B: 1	6	$C_{18}H_{27}NO_{12}$	H^+	450.16060	1	67.49%	0.20%
A: 5 B: 1	6	$C_{18}H_{27}NO_{12}$	Na^+	472.14255	1	67.98%	0.25%
A: 5 B: 1	6	$C_{18}H_{27}NO_{12}$	K^+	488.11648	1	61.28%	0.19%
A: 6 B: 0	6	$C_{18}H_{26}O_{13}$	H^+	451.14462	1	54.51%	0.16%
A: 6 B: 0	6	$C_{18}H_{26}O_{13}$	Na^+	473.12656	1	62.48%	0.18%
A: 6 B: 0	6	$C_{18}H_{26}O_{13}$	K^+	489.10050	1	60.95%	0.18%
A: 1 B: 6	7	$C_{21}H_{36}N_6O_9$	K^+	555.21753	1	58.67%	0.18%
A: 2 B: 5	7	$C_{21}H_{35}N_5O_{10}$	H^+	518.24567	1	60.69%	0.17%
A: 2 B: 5	7	$C_{21}H_{35}N_5O_{10}$	Na^+	540.22761	1	54.68%	0.22%
A: 2 B: 5	7	$C_{21}H_{35}N_5O_{10}$	K^+	556.20155	1	59.94%	0.18%
A: 3 B: 4	7	$C_{21}H_{34}N_4O_{11}$	H^+	519.22968	1	49.77%	0.18%
A: 3 B: 4	7	$C_{21}H_{34}N_4O_{11}$	Na^+	541.21163	1	53.33%	0.24%
A: 3 B: 4	7	$C_{21}H_{34}N_4O_{11}$	K^+	557.18557	1	61.25%	0.18%
A: 4 B: 3	7	$C_{21}H_{33}N_3O_{12}$	H^+	520.21370	1	51.62%	0.20%
A: 4 B: 3	7	$C_{21}H_{33}N_3O_{12}$	Na^+	542.19564	1	54.37%	0.25%
A: 4 B: 3	7	$C_{21}H_{33}N_3O_{12}$	K^+	558.16958	1	60.89%	0.18%
A: 5 B: 2	7	$C_{21}H_{32}N_2O_{13}$	H^+	521.19772	1	59.44%	0.21%
A: 5 B: 2	7	$C_{21}H_{32}N_2O_{13}$	Na^+	543.17966	1	61.74%	0.23%
A: 5 B: 2	7	$C_{21}H_{32}N_2O_{13}$	K^+	559.15360	1	60.24%	0.17%

Composition	Nb units	MF	Ioniz.	em [m/z]	Charge	Similarity	Quantity
A: 6 B: 1	7	$C_{21}H_{31}NO_{14}$	H^+	522.18173	1	55.18%	0.19%
A: 6 B: 1	7	$C_{21}H_{31}NO_{14}$	Na^+	544.16368	1	65.16%	0.19%
A: 7 B: 0	7	$C_{21}H_{30}O_{15}$	Na^+	545.14769	1	64.09%	0.15%
A: 5 B: 3	8	$C_{24}H_{37}N_3O_{14}$	H^+	592.23483	1	57.28%	0.17%
A: 5 B: 3	8	$C_{24}H_{37}N_3O_{14}$	Na^+	614.21677	1	59.60%	0.20%
A: 6 B: 2	8	$C_{24}H_{36}N_2O_{15}$	H^+	593.21884	1	59.70%	0.18%
A: 6 B: 2	8	$C_{24}H_{36}N_2O_{15}$	Na^+	615.20079	1	64.76%	0.17%
A: 6 B: 2	8	$C_{24}H_{36}N_2O_{15}$	K^+	631.17473	1	66.11%	0.13%
A: 7 B: 1	8	$C_{24}H_{35}NO_{16}$	H^+	594.20286	1	62.29%	0.17%
A: 7 B: 1	8	$C_{24}H_{35}NO_{16}$	Na^+	616.18480	1	64.14%	0.15%
A: 7 B: 1	8	$C_{24}H_{35}NO_{16}$	K^+	632.15874	1	63.20%	0.13%
A: 8 B: 0	8	$C_{24}H_{34}O_{17}$	H^+	595.18688	1	59.63%	0.15%
A: 5 B: 4	9	$C_{27}H_{42}N_4O_{15}$	K^+	701.22782	1	66.71%	0.16%
A: 6 B: 3	9	$C_{27}H_{41}N_3O_{16}$	Na^+	686.23790	1	61.12%	0.16%
A: 7 B: 2	9	$C_{27}H_{40}N_2O_{17}$	Na^+	687.22192	1	65.67%	0.16%
A: 8 B: 1	9	$C_{27}H_{39}NO_{18}$	H^+	666.22399	1	66.51%	0.17%
A: 7 B: 3	10	$C_{30}H_{45}N_3O_{18}$	Na^+	758.25903	1	63.95%	0.14%

Polymers grouped by monoisotopic mass

Composition				Monoisotopic mass	Quantity
A: 0 B: 3	3	$C_9H_{17}N_3O_4$	K^+	231.1219	0.28%
A: 1 B: 2	3	$C_9H_{16}N_2O_5$	H^+	232.1059	1.65%
A: 1 B: 2	3	$C_9H_{16}N_2O_5$	Na^+		
A: 1 B: 2	3	$C_9H_{16}N_2O_5$	K^+		
A: 2 B: 1	3	$C_9H_{15}NO_6$	H^+	233.0899	1.58%
A: 2 B: 1	3	$C_9H_{15}NO_6$	Na^+		
A: 2 B: 1	3	$C_9H_{15}NO_6$	K^+		
A: 3 B: 0	3	$C_9H_{14}O_7$	K^+	234.0740	1.01%
A: 3 B: 0	3	$C_9H_{14}O_7$	H^+		
A: 3 B: 0	3	$C_9H_{14}O_7$	Na^+		
A: 0 B: 4	4	$C_{12}H_{22}N_4O_5$	K^+	302.1590	0.37%
A: 1 B: 3	4	$C_{12}H_{21}N_3O_6$	K^+	303.1430	0.40%
A: 2 B: 2	4	$C_{12}H_{20}N_2O_7$	K^+	304.1271	0.36%
A: 3 B: 1	4	$C_{12}H_{19}NO_8$	H^+	305.1111	1.35%
A: 3 B: 1	4	$C_{12}H_{19}NO_8$	Na^+		

		Composition		Monoisotopic mass	Quantity
A: 3 B: 1	4	$C_{12}H_{19}NO_8$	K^+		
A: 4 B: 0	4	$C_{12}H_{18}O_9$	H^+	306.0951	0.60%
A: 4 B: 0	4	$C_{12}H_{18}O_9$	K^+		
A: 0 B: 5	5	$C_{15}H_{27}N_5O_6$	K^+		
A: 0 B: 5	5	$C_{15}H_{27}N_5O_6$	Na^+	373.1961	0.59%
A: 0 B: 5	5	$C_{15}H_{27}N_5O_6$	H^+		
A: 1 B: 4	5	$C_{15}H_{26}N_4O_7$	K^+	374.1801	0.22%
A: 2 B: 3	5	$C_{15}H_{25}N_3O_8$	K^+		
A: 2 B: 3	5	$C_{15}H_{25}N_3O_8$	H^+	375.1642	1.12%
A: 2 B: 3	5	$C_{15}H_{25}N_3O_8$	Na^+		
A: 3 B: 2	5	$C_{15}H_{24}N_2O_9$	H^+		
A: 3 B: 2	5	$C_{15}H_{24}N_2O_9$	Na^+	376.1482	1.16%
A: 3 B: 2	5	$C_{15}H_{24}N_2O_9$	K^+		
A: 4 B: 1	5	$C_{15}H_{23}NO_{10}$	H^+		
A: 4 B: 1	5	$C_{15}H_{23}NO_{10}$	Na^+	377.1322	0.88%
A: 4 B: 1	5	$C_{15}H_{23}NO_{10}$	K^+		
A: 5 B: 0	5	$C_{15}H_{22}O_{11}$	Na^+	378.1162	0.21%
A: 0 B: 6	6	$C_{18}H_{32}N_6O_7$	K^+		
A: 0 B: 6	6	$C_{18}H_{32}N_6O_7$	Na^+	444.2332	0.54%
A: 0 B: 6	6	$C_{18}H_{32}N_6O_7$	H^+		
A: 1 B: 5	6	$C_{18}H_{31}N_5O_8$	Na^+	445.2173	0.38%
A: 1 B: 5	6	$C_{18}H_{31}N_5O_8$	H^+		
A: 2 B: 4	6	$C_{18}H_{30}N_4O_9$	Na^+	446.2013	0.51%
A: 2 B: 4	6	$C_{18}H_{30}N_4O_9$	H^+		
A: 3 B: 3	6	$C_{18}H_{29}N_3O_{10}$	H^+	447.1853	0.62%
A: 3 B: 3	6	$C_{18}H_{29}N_3O_{10}$	Na^+		
A: 4 B: 2	6	$C_{18}H_{28}N_2O_{11}$	H^+		
A: 4 B: 2	6	$C_{18}H_{28}N_2O_{11}$	K^+	448.1693	0.81%
A: 4 B: 2	6	$C_{18}H_{28}N_2O_{11}$	Na^+		
A: 5 B: 1	6	$C_{18}H_{27}NO_{12}$	Na^+		
A: 5 B: 1	6	$C_{18}H_{27}NO_{12}$	H^+	449.1533	0.64%
A: 5 B: 1	6	$C_{18}H_{27}NO_{12}$	K^+		
A: 6 B: 0	6	$C_{18}H_{26}O_{13}$	Na^+		
A: 6 B: 0	6	$C_{18}H_{26}O_{13}$	K^+	450.1373	0.52%
A: 6 B: 0	6	$C_{18}H_{26}O_{13}$	H^+		
A: 1 B: 6	7	$C_{21}H_{36}N_6O_9$	K^+	516.2544	0.18%
A: 2 B: 5	7	$C_{21}H_{35}N_5O_{10}$	H^+	517.2384	0.57%
A: 2 B: 5	7	$C_{21}H_{35}N_5O_{10}$	K^+		

	Composition		Monoisotopic mass	Quantity
A: 2 B: 5	7	$C_{21}H_{35}N_5O_{10}$ Na^+		
A: 3 B: 4	7	$C_{21}H_{34}N_4O_{11}$ K^+	518.2224	0.60%
A: 3 B: 4	7	$C_{21}H_{34}N_4O_{11}$ Na^+		
A: 3 B: 4	7	$C_{21}H_{34}N_4O_{11}$ H^+		
A: 4 B: 3	7	$C_{21}H_{33}N_3O_{12}$ K^+	519.2064	0.62%
A: 4 B: 3	7	$C_{21}H_{33}N_3O_{12}$ Na^+		
A: 4 B: 3	7	$C_{21}H_{33}N_3O_{12}$ H^+		
A: 5 B: 2	7	$C_{21}H_{32}N_2O_{13}$ Na^+	520.1904	0.61%
A: 5 B: 2	7	$C_{21}H_{32}N_2O_{13}$ K^+		
A: 5 B: 2	7	$C_{21}H_{32}N_2O_{13}$ H^+		
A: 6 B: 1	7	$C_{21}H_{31}NO_{14}$ Na^+	521.1745	0.38%
A: 6 B: 1	7	$C_{21}H_{31}NO_{14}$ H^+		
A: 7 B: 0	7	$C_{21}H_{30}O_{15}$ Na^+	522.1585	0.15%
A: 5 B: 3	8	$C_{24}H_{37}N_3O_{14}$ Na^+	591.2276	0.37%
A: 5 B: 3	8	$C_{24}H_{37}N_3O_{14}$ H^+		
A: 6 B: 2	8	$C_{24}H_{36}N_2O_{15}$ K^+	592.2116	0.49%
A: 6 B: 2	8	$C_{24}H_{36}N_2O_{15}$ Na^+		
A: 6 B: 2	8	$C_{24}H_{36}N_2O_{15}$ H^+		
A: 7 B: 1	8	$C_{24}H_{35}NO_{16}$ Na^+	593.1956	0.46%
A: 7 B: 1	8	$C_{24}H_{35}NO_{16}$ K^+		
A: 7 B: 1	8	$C_{24}H_{35}NO_{16}$ H^+		
A: 8 B: 0	8	$C_{24}H_{34}O_{17}$ H^+	594.1796	0.15%
A: 5 B: 4	9	$C_{27}H_{42}N_4O_{15}$ K^+	662.2647	0.16%
A: 6 B: 3	9	$C_{27}H_{41}N_3O_{16}$ Na^+	663.2487	0.16%
A: 7 B: 2	9	$C_{27}H_{40}N_2O_{17}$ Na^+	664.2327	0.16%
A: 8 B: 1	9	$C_{27}H_{39}NO_{18}$ H^+	665.2167	0.17%
A: 7 B: 3	10	$C_{30}H_{45}N_3O_{18}$ Na^+	735.2698	0.14%

Analytical report

Page under construction...

aA MALDI, 4cyc, 1mg LHS-1D

Analysis parameters

Ionizations

MF	Monoisotopic mass	m/z mass
H^+	1.00783	1.00728
Na^+	22.98977	22.98922
K^+	38.96371	38.96316

End groups

α	ω	Color
H	OH	

Monomers

	Description	mf	Monoisotopic mass	min	max
A	Lactic acid	$C_3H_4O_2$	72.02113	0	100
B	Alanine	C_3H_5NO	71.03711	0	100

Experimental spectrum

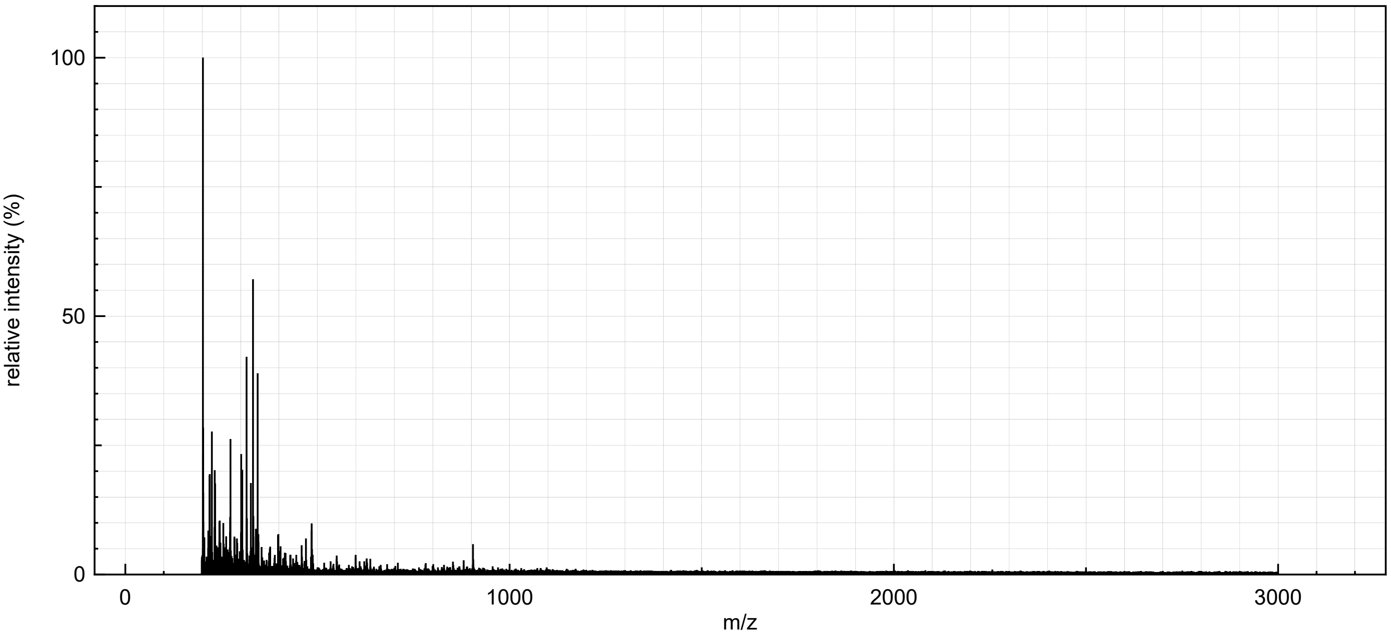

Reconstructed spectrum

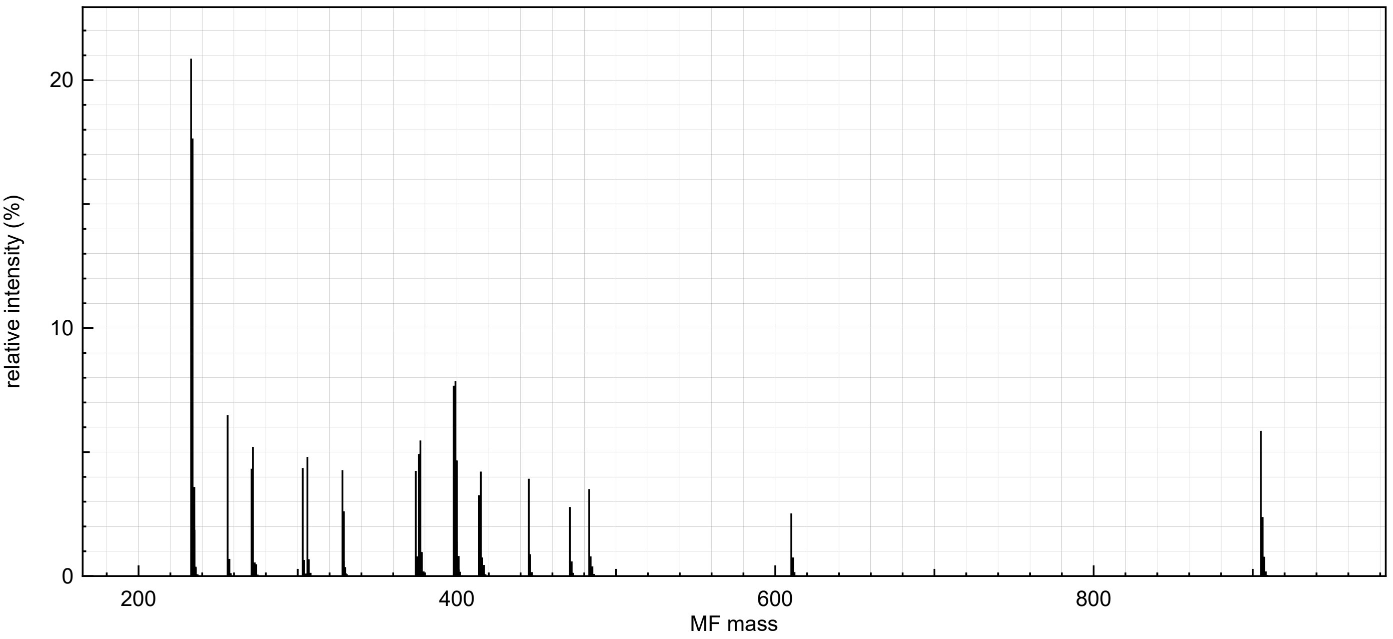

Error (ppm) versus m/z

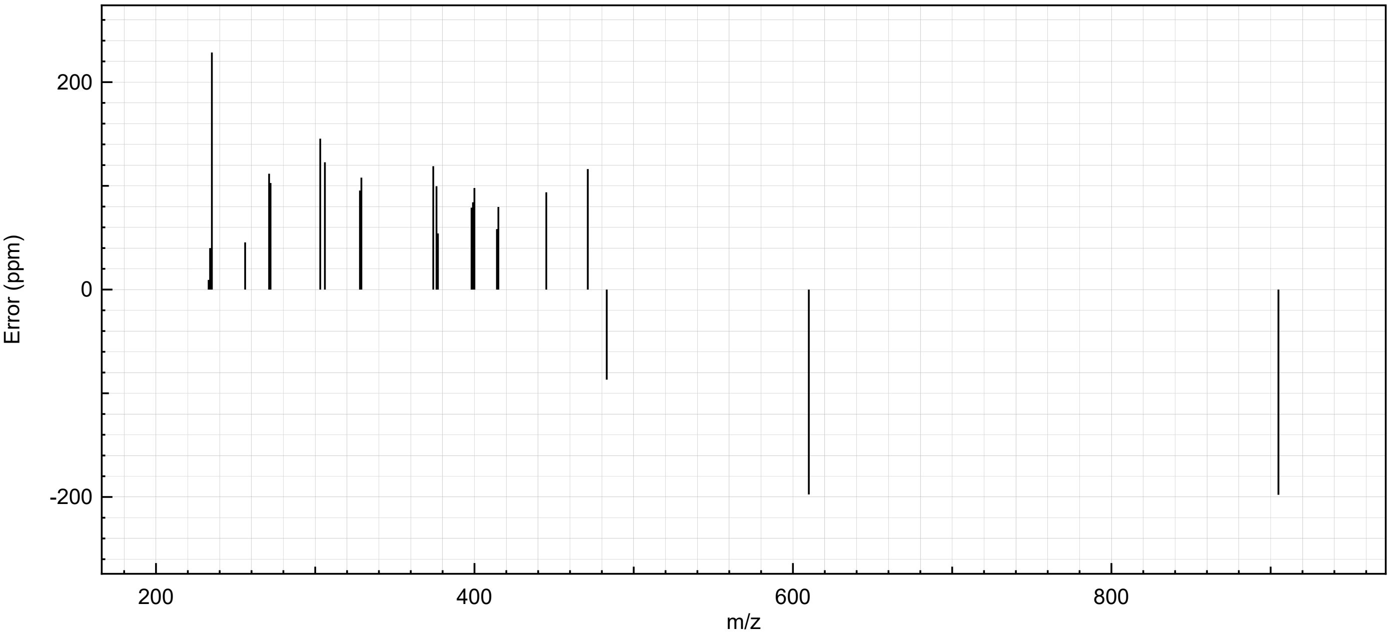

Analysis result

Percent of peaks covered: 6.30%
Number of assigned peaks: 23

Polymers [m/z]

Composition	Nb units	MF	Ioniz.	em [m/z]	Charge	Similarity	Quantity
A: 1 B: 2	3	$C_9H_{16}N_2O_5$	H^+	233.11320	1	60.93%	0.51%
A: 1 B: 2	3	$C_9H_{16}N_2O_5$	K^+	271.06908	1	36.11%	0.47%
A: 2 B: 1	3	$C_9H_{15}NO_6$	Na^+	256.07916	1	69.42%	0.26%
A: 2 B: 1	3	$C_9H_{15}NO_6$	H^+	234.09721	1	68.56%	0.32%
A: 2 B: 1	3	$C_9H_{15}NO_6$	K^+	272.05310	1	32.92%	0.65%
A: 3 B: 0	3	$C_9H_{14}O_7$	H^+	235.08123	1	41.45%	0.22%
A: 0 B: 4	4	$C_{12}H_{22}N_4O_5$	H^+	303.16630	1	38.27%	0.38%
A: 3 B: 1	4	$C_{12}H_{19}NO_8$	H^+	306.11834	1	57.19%	0.21%
A: 3 B: 1	4	$C_{12}H_{19}NO_8$	Na^+	328.10029	1	55.87%	0.22%
A: 4 B: 0	4	$C_{12}H_{18}O_9$	Na^+	329.08430	1	51.17%	0.18%
A: 0 B: 5	5	$C_{15}H_{27}N_5O_6$	H^+	374.20341	1	51.87%	0.20%
A: 2 B: 3	5	$C_{15}H_{25}N_3O_8$	H^+	376.17144	1	54.30%	0.24%
A: 2 B: 3	5	$C_{15}H_{25}N_3O_8$	Na^+	398.15339	1	52.81%	0.30%
A: 2 B: 3	5	$C_{15}H_{25}N_3O_8$	K^+	414.12732	1	52.21%	0.23%
A: 3 B: 2	5	$C_{15}H_{24}N_2O_9$	Na^+	399.13740	1	61.72%	0.25%
A: 3 B: 2	5	$C_{15}H_{24}N_2O_9$	H^+	377.15546	1	60.80%	0.21%
A: 3 B: 2	5	$C_{15}H_{24}N_2O_9$	K^+	415.11134	1	51.78%	0.25%
A: 4 B: 1	5	$C_{15}H_{23}NO_{10}$	Na^+	400.12142	1	58.88%	0.19%
A: 0 B: 6	6	$C_{18}H_{32}N_6O_7$	H^+	445.24052	1	64.65%	0.17%
A: 0 B: 6	6	$C_{18}H_{32}N_6O_7$	K^+	483.19641	1	47.35%	0.28%
A: 4 B: 2	6	$C_{18}H_{28}N_2O_{11}$	Na^+	471.15853	1	60.91%	0.15%
A: 1 B: 7	8	$C_{24}H_{41}N_7O_{10}$	Na^+	610.28071	1	63.75%	0.16%
A: 12 B: 0	12	$C_{36}H_{50}O_{25}$	Na^+	905.25334	1	81.46%	0.22%

Polymers grouped by monoisotopic mass

Composition				Monoisotopic mass	Quantity
A: 1 B: 2<br>A: 1 B: 2	3<br>3	$C_9H_{16}N_2O_5$<br>$C_9H_{16}N_2O_5$	H^+<br>K^+	232.1059	0.98%
A: 2 B: 1<br>A: 2 B: 1	3<br>3	$C_9H_{15}NO_6$<br>$C_9H_{15}NO_6$	Na^+<br>H^+	233.0899	1.24%

Composition				Monoisotopic mass	Quantity
A: 2 B: 1	3	$C_9H_{15}NO_6$	K^+		
A: 3 B: 0	3	$C_9H_{14}O_7$	H^+	234.0740	0.22%
A: 0 B: 4	4	$C_{12}H_{22}N_4O_5$	H^+	302.1590	0.38%
A: 3 B: 1<br>A: 3 B: 1	4<br>4	$C_{12}H_{19}NO_8$<br>$C_{12}H_{19}NO_8$	H^+<br>Na^+	305.1111	0.43%
A: 4 B: 0	4	$C_{12}H_{18}O_9$	Na^+	306.0951	0.18%
A: 0 B: 5	5	$C_{15}H_{27}N_5O_6$	H^+	373.1961	0.20%
A: 2 B: 3<br>A: 2 B: 3<br>A: 2 B: 3	5<br>5<br>5	$C_{15}H_{25}N_3O_8$<br>$C_{15}H_{25}N_3O_8$<br>$C_{15}H_{25}N_3O_8$	H^+<br>Na^+<br>K^+	375.1642	0.77%
A: 3 B: 2<br>A: 3 B: 2<br>A: 3 B: 2	5<br>5<br>5	$C_{15}H_{24}N_2O_9$<br>$C_{15}H_{24}N_2O_9$<br>$C_{15}H_{24}N_2O_9$	Na^+<br>H^+<br>K^+	376.1482	0.70%
A: 4 B: 1	5	$C_{15}H_{23}NO_{10}$	Na^+	377.1322	0.19%
A: 0 B: 6<br>A: 0 B: 6	6<br>6	$C_{18}H_{32}N_6O_7$<br>$C_{18}H_{32}N_6O_7$	H^+<br>K^+	444.2332	0.46%
A: 4 B: 2	6	$C_{18}H_{28}N_2O_{11}$	Na^+	448.1693	0.15%
A: 1 B: 7	8	$C_{24}H_{41}N_7O_{10}$	Na^+	587.2915	0.16%
A: 12 B: 0	12	$C_{36}H_{50}O_{25}$	Na^+	882.2641	0.22%
